# High-resolution map of chromatin accessibility - insights into the focused binding of a large number of transcription factors

**DOI:** 10.1186/s13072-026-00665-2

**Published:** 2026-02-22

**Authors:** Iris Zhu, David Landsman

**Affiliations:** Division of Intramural Research, National Library of Medicine, National Institutes of Health, Bethesda, MD USA

**Keywords:** Chromatin accessibility, Focused binding of a large number of TFs, Nucleosome depletion

## Abstract

**Background:**

Emerging evidence has shown the common occupancy of dozens to hundreds of transcription factors (TFs) on cis-regulatory elements (CREs), yet the underlying details are largely unknown.

**Results:**

In this study, leveraging extensive collections of TF ChIP-seq data of more than 1000 TFs in human HepG2 and K562 cells, we located highly focused TF binding sites (FBSs) within CREs as single-nucleosome depleted regions, which accommodate the majority of the total TF binding events. Approximately 25,000 strong FBSs were identified in each cell type. For more than 90% of TFs, including some pioneer factors such as GATA1 and JUN, their binding sites out of FBSs barely show nucleosome depletion. Essential cellular function related motifs and phenotypically causal variants are strongly enriched in the FBSs, but not in their immediate flanking regions within CREs. Most TFs bind to FBSs not containing their canonical motifs.

**Conclusion:**

Our study revealed the critical connection between highly focused TF binding and the nucleosome depleted status of DNA in vivo. Meanwhile, we constructed high-resolution maps of chromatin accessibility at distal CREs in the two human cells. We propose a model of TF co-binding in vivo and suggest that a short DNA residence time of most TFs underlies the requirement of a large number of TFs for sustained nucleosome depletion at CREs.

**Supplementary Information:**

The online version contains supplementary material available at 10.1186/s13072-026-00665-2.

## Introduction

Collective binding of transcription factors (TFs) to DNA is essential for gene regulatory function in multicellular organisms [1–5]. In the traditional view of collective TF binding, a cis regulatory element (CRE) like an enhancer, is bound by a small set of TFs that recognize their preferable sequence motifs. The combinatorial recognition of DNA defines unique genomic locations resulting in specific gene regulatory events. With the expanding catalog of TF ChIP-seq data, it was found that some CREs are bound by large number of TFs. The term high-occupancy target (HOT) was created for such genomic sites. In recent years, thanks to the collaborative ENCODE project, ChIP-seq experiments have been done for hundreds of TFs in a few cell lines. Depending on the number of available TF ChIP-seq data in a studied cell type and the criteria for HOT loci set by individual research groups, the number of HOT loci ranges from about 3,000 to 26,000 in different cell types and the number of TFs associated with each locus ranges from dozens to more than four hundreds [6–8]. The mechanisms underlying HOT loci formation, and the function and sequence features of HOT loci have been a research interest for years. It is now quite clear that, except for a small number of HOT loci that are the results of ChIP artifacts, the vast majority of HOT loci are either promoter or enhancer elements that play important functional roles in gene regulation. There are nearly 1000 TFs expressed in each cell type in human. To date, for the majority of cell types, less than 20 TF ChIP-seq data are available and that is why HOT loci have only been identified and studied in a few cell types. Studies regarding the cumulative TF binding showed that, with the number of TF binding data continue to expand, we would expect to discover more CREs as HOT loci [6, 9].

Regarding CREs ranging from hundreds to thousands base pairs (bps) in length bound by dozens to hundreds of TF/cofactors, many critical details remain elusive. A basic question is: How do hundreds of TFs associate with a long stretch of DNA physically, in other words, how does a HOT locus form? DNA in cell nucleus is occupied either by nucleosomes or DNA-binding proteins. CREs typically overlap Dnase I-seq or ATAC-seq signals, which indicates nucleosome depletion [10, 11]. On the other hand, studies have shown that most TF binding sites are nucleosome free region flanked by well positioned nucleosome [11, 12]. Combined, these results pose a puzzling question: Using an example of a 1 kb-long CRE bound by 100 TFs -- 1 kb DNA would normally accommodate 5–6 nucleosomes -- How do the nucleosomes and the 100 TFs distribute along the DNA and how do their competitions play out so that we observe well phased nucleosomes flanking most TFs while the whole site is nucleosome depleted?

Cooperative binding of TFs to DNA has been revealed with great details at molecular level. For instance, TFs form complex as dimer, trimer or tetramer, homomeric or heteromeric in nature, which enables their DNA binding or greatly increases the DNA binding affinity of the monomer TFs [4, 13, 14]. With a different mechanism, TF cooperativity can be mediated through local DNA structure change, in the absence of direct TF-TF interactions [15]. TF cooperativity also occurs through passive competition of TFs with nucleosomes [11, 16]. The technologies adapted in these research, e.g. structural studies, CAP-SELEX applied to TF bound DNAs [15] or single molecule printing methods [16], are currently limited to investigate the co-binding of 2–5 TFs, and often in in vitro system. In a living human cell more than one thousand TFs and cofactors are expressed, and TF binding is highly dynamic. How do TFs of this large number cooperate with each other in cell nucleus remains an intriguing question. In this study, we attempt to understand the nature and mechanism of collective binding of large number of TFs on CREs. Leveraging the deep collection of ChIP-seq data of more than 1000 TF/cofactors in two human cells HepG2 and K562, we found that, within CREs hundreds to thousands bases pairs long, highly focused TF binding can be pinpointed to narrow regions mostly less than 200 bp, which account for more than half of the binding events. Incorporating analysis of MNase-seq data, motif composition and functional genomics data like eQTLs, we revealed high resolution maps of both chromatin accessibility and functional sequence features at the distal CREs in the two human cells. Our results established the critical connections between the focused binding of a large number of TFs and sustained nucleosome depletion at CREs. We propose a general mode of how the co-bindings of large number of TFs occur in cell nucleus, which also explains the formation of the observed HOT loci in human genome.

## Methods

### ChIP-seq data collection and filtering

We downloaded the data from the ENCODE portal [17] (https://www.encodeproject.org/) with the following identifiers: ChIP-seq, Transcription factor, HepG2, K562, bed narrowpeak and hg38. We used data from two laboratories, Richard Meyer and Michael Snyder’s group, since the data from the other eight labs account for less than 10% of the total and are mostly repeated experiments of the former two groups. For HepG2 cell, there are 634 TFs from Meyer group and 90 TFs from Snyder group. For K562 cells, there are 344 TFs from Snyder group and 135 from Meyer group. Data sets with less than 2000 peaks were discarded, because these very likely represent failed ChIP-seq experiments. Data sets with replicates (ChIP-seq of the same TF from two research groups also considered replicates) were further processed based on the following rules: [1] For the TFs with two replicates, if the peak number of one replicate is more than 3 times as the other replicate and the lesser one have less than 3000 peaks, the latter is considered a failed experiment. The replicate with higher peak number is used [2]. For the TFs with two replicates, if the number of the overlapping peaks between the two replicates is more than 3000 or greater than 40% of either of the replicate, the overlapping set was used for the TF. If a TF with two replicates does not meet either of the two conditions, the data was not used [3]. If there are more than 2 replicates for a TF, select two out of all replicates with the highest percentage or the highest number of overlapping peaks and apply rule 1 and 2. After the processing steps, the combined data include 598 TFs for HepG2 cell and 409 TFs from K562 cells. The ChIP-seq peaks have a median width of 501 bp from HepG2 cell, and 381 bp from K562 cell. We selected the center 300 bp for each peak to better represent the strongest signal and used the center 300 bp windows to obtain the MP, using the merge function of Bedtools program [18].

### ChIP-seq, ATAC-seq, MNase-seq analysis and RNA-seq analysis

Raw sequences are downloaded from SRA (accession numbers in Supplementary Table 1). Unfiltered sequencing reads were aligned to the human reference genome hg38 using Bowtie2 [19] with default parameters. For ChIP-seq and ATAC-seq, peak regions were identified using MACS3 [20] with cutoff q-value of 0.01. Calculations of coverage and identification of overlapping binding regions were performed with the “chipseq” and “GenomicRanges” packages in BioConductor [21]. In analyzing MNase-seq data, since the reads are from the ends of the mononucleosome particle, to best visualize the nucleosome positions, all the reads were shifted 50 bps to the middle of the corresponding nucleosome core. RNA-seq analysis was performed with htseq-count [22] and “edgeR” package in BioConductor.

Active promoters are defined as 2 kb upstream to 1 kb downstream of transcription start sites (TSSs) of the expressed genes (RPKM > 1). Enhancers MPs are defined as those overlapping H3K27ac/H3k4me1 signals but not any active promoters. For the enhancer MPs, we assigned the active promoter(s) within 50 kb, as their associated promoter(s). If no active promoter was found within 50 kb, the nearest active promoter was assigned.

### Overlap of the merged peaks with chromatin States annotation from chromhmm

We computed overlap of the merged peaks (MPs) with chromatin states in HepG2 and K562 cells downloaded from ENCODE (accession # in Table S1). The annotation include 18 chromHMM states: promoter (1_TssA, 2_TssFlnk, 3_TssFlnkU, 4_TssFlnkD, 14_TssBiv), transcribed (5_Tx, 6_TxWk), enhancer (7_EnhG1, 8_EnhG2, 9_EnhA1, 10_EnhA2, 11_EnhWk, 15_EnhBiv), polycomb (16_ReprPC, 17_ReprPCWk), heterochromatin (13_Het), ZNF repeats (12_ZNF/Rpts), and quiescent (18_Quies). For each MP we computed the fraction of bases that overlapped each chromatin state using Bedtools coverage. We assigned each peak to the chromatin state with which it shared the most bases, except for the quiescent state; we only assigned a peak to a quiescent state if all bases of a peak were found within a quiescent state.

### Identification of highly focused TF binding sites (FBSs) within merged peaks

The merged peaks (MPs) were ranked based on the number of the overlapping individual TF ChIP-seq peaks in each MP. The top 30,000 most TF-enriched MPs in HepG2 cell and the top 50,000 MPs in K562 cell were used for identification of FBSs. Each individual TF ChIP-seq peak was represented by the coordinate of its center point, denoted as TF points for the rest of this section. The FBSs were identified as following: Step1: The MPs were scanned for the count of TF points at consecutive non-overlapping 10-bp bins. First, we made a TF point density probability table (Fig S2A). The counts at approximately 90% level (90% of bins have smaller counts), which are 4 per 10 bp for HepG2 and 2 per10 bp for K562, were used as the threshold count C_T_. Bins with counts equal or above C_T_ were identified and consecutive bins were combined into one window. Windows below 40 bp were discarded and the rest were saved as candidate FBSs (*n* = 49567 for HepG2 and *n* = 76421 for K562).

**Fig. 1:**
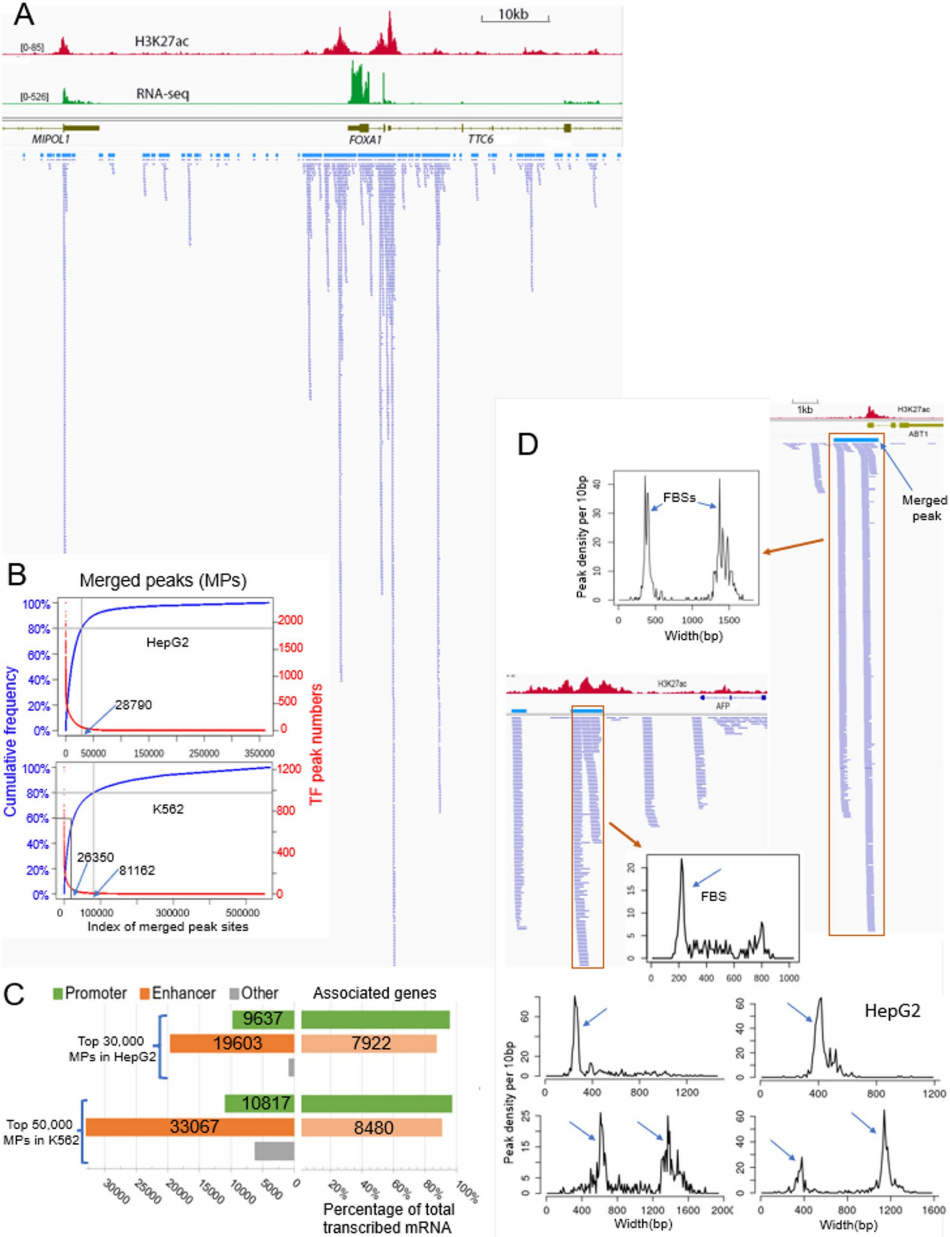
Identification of highly focused TF binding sites from deep collection of TF ChIP-seq data. **A** A snapshot of genome view at FOXA1 gene region in HepG2 cell. RNA-seq and the H3K27ac signals on top and bed files of all TF ChIP-seq peaks at the bottom. **B** The merged peaks (MPs) are ranked based on the number of overlapping individual TF peaks of each site (red lines) and plotted with cumulative curves (blue lines). The arrows point to the numbers of MPs at the indicated cumulative levels. **C** The number of promoters and enhancers from the top 30,000 and 50,000 most TF-enriched MPs in HepG2 and K562 cells, respectively (left), and the percentage of transcribed mRNA of their associated genes out of the total mRNA (right). **D** Scanning for the density of center points of individual TF peaks within each MP to identify highly focus binding sites. Shown are examples from HepG2 cell data

Step 2: To profile TF point counts distribution per unit length (ranging from 80 to 200 bp) in the MPs. Since 95% of candidate FBSs have a width below 200 bps, we first did an exhaustive sampling of TF point counts for 200 bp window within the MPs: We scanned a window of 200 bps along all the MPs at a step of 20 bp and counted the total TF points in the window. We call it the TF point distribution for 200 bp, namely D_200bp. The distribution has a right skewed long tail shape (Fig S2B), which indicates that the majority area of the MPs has close to zero TF point counts. A threshold percentile at the right tail region, e.g. the count number at 95% level, can be used to select FBSs. Next, since candidate FBSs differ in their width, height and shape of the TF point plot (Fig. 1D), we repeated the distribution profiling for different window sizes of 180, 160, 140, 120, 100 and 80 bp. For all the window sizes the distribution has the similar right skewed long-tail shape, but different values of counts (Fig. S2B).

Step 3: To test the threshold percentile levels from the above profiled distributions for FBS selection. We divided the candidate FBSs into groups of different widths: below 100 bp, 100–120 bp, 120–140 bp, 140–160 bp, 160–180 bp, 180–200 bp and above 200 bp. The distribution D_100bp will be used to select FBSs of 100–120 bp, D_120bp for FBSs of 120–140 bp, and so on. D_200bp will be used for FBSs above 200 bp and D_80bp for FBSs below 100 bp. FBSs are selected based on the count at a given threshold percentile of the corresponding distribution. For example, the count at the right tail 95% point of the D_100bp is 58. There are 3980 FBSs of width 100–120 bp with the total counts above 57, which were marked as FBS_P95 at 100 bp. Different threshold percentile levels result in different numbers of selected FBSs. A sensitivity plot shows the total number of selected FBSs versus the threshold percentile of 85%, 90%, 95% and 98% (Figure S2C).

### Comparison of nucleosome depletion

The average MNase-seq signal of the binding sites from three groups were calculated for each TF: (1) sites inside the strong FBSs (FBSs_P95), (2) sites outside of any FBSs (FBSs_P90) and (3) the sites with less than 10 other binding partners (lonely sites). The group 3 is a subset of group2. FBSs located at promoter regions are not included in the calculation. The relative nucleosome depletion of group3 compared to group1 is calculated as F = (G3_***high***_ - G3_***low***_)/(G1_***high***_ - G1_***low***_), in which G1 and G3 represent the MNase-seq signal plots of the above group 1 and group3, high and low represent the value at the edge and the valley of the center nucleosome depleted region in the MNase-seq signal plot. The value of (G_***high***_ - G_***low***_) indicates the degree of nucleosome depletion. F value of 50% or above is defined as strong nucleosome depletion.

### Motif analysis

For de novo motif discovery, we used meme program [23] on the strong FBSs (FBS_P95) at promoters and distal regions in HepG2 or K562 cell with the following parameters: -text -dna -revcomp -mod anr -nmotifs 50 -evt 0.001 -maxsize 30,000,000. Those identified motifs with low sequence complexity were discarded. TFBS enrichment analysis is run with the JASPER tools https://bitbucket.org/CBGR/jaspar_enrichment/src/master/, using the “twoSets” subcommand. The TFBS analysis were done for three different groups: (1) The FBSs sites, extended to 180 bp if shorter, based on the consideration that a single nucleosome depleted region has the length of about 180 bp. (2) The flanking 200 bp windows on both side of the FBSs in group1. (3) The middle 500 bp window of the top 30,000 most TF-enriched MPs. Peaks at promoter regions and distal regions were analyzed separately. For each group, randomly selected genomic regions of the same length (180 bp, 200 bp and 500 bp for the three groups respectively) with matching GC content, and the sample size at least 3 times bigger were used as control. The result files include 842 TF matrices, their enrichment scores (p-value) and the TFBS family they belong to. For TF motifs enriched (p-value < 1e-5) in at each one of the about three groups, the comparison of the enrichment p-value among the three groups were shown in Fig. 4C. Motif search for the results in Fig. 5C is performed with searchSeq() function in Bioconductor with TFBSTools package [24] and JASPAR 2022 motif database [25]. The p-value of the motif PWM match was calculated with the method TFMPvalue and a threshold p-value of 0.0001 was applied for a JASPAR PWM identification.

### Analysis of QTL and GWAS SNP enrichment

The enrichment fold of QTLs and GWAS SNPs was calculated as the density of the variants in the sample sites divided by the density expected by chance: (N_**var**__S/length_S)/(N_**var**__T/length_T), in which N_**var**__S is the number of the variants located within the sample of interest, e.g. distal FBSs, length_S is the total lengths of the sample of interest (in base pairs), N_**var**__T is the total number of variants and length_T is the total length of the genome. The enrichment fold of variants in FBSs compared to MPs was calculated similarly: (N_**var**__FBS/length_FBS)/(N_**var**__MP/length_MP). The p-value is calculated with binomial test in R. For raQTLs and caQTLs, the coordinates of the downloaded QTLs were mapped for hg19. They were converted to hg38 coordinates with UCSC liftover.

### Pair-wise co-occurrence of individual TFs and TF families at FBSs

A TF is defined as bound to a FBS if the center 10 bp of the TF ChIP-seq peak overlap at least 1 bp with the FBS (extended to 180 bp if shorter). When the ChIP-seq peaks of two different TFs are associated with the same FBS, it is considered as a co-occurrence of the two TFs. The co-occurrence rate of TF A and TF B was calculated as N_***FBS_both***_/max(N_***FBS_A***_, N_***FBS_B***_), in which N_***FBS_both***_ is the number of the FBSs that both TF A and B are bound to and N_***FBS_A***_, N_***FBS_B***_ are the numbers of FBSs bound by TF A and B, respectively. For instance, 9550 and 12,876 out of the total 16,480 distal FBSs in HepG2 are bound by ADNP and FOXP4, respectively, among which 8623 FBSs are bound by both TFs. The co-occurrence rate of ADNP and FOXP4 is 8623/12,876 = 0.67. In this way, a square matrix with size *N***x***N* is obtained, in which *N* is the number of total TFs (*N* = 598 for HepG2 and *N* = 409 for K562). TF groups with high level of co-binding among each other was identified with hierarchical clustering and presented with heatmaps using the function heatmap.2 from the “gplots” package in Bioconductor. FBSs located at promoter regions and distal regions were analyzed separately. Co-occupancy rates among any two TF families were calculated as the percentage of FBSs at which both TF families are present. Since cofactors do not belong to any of the TF families, each cofactor was incorporated in the analysis on its own. Thus, the size of the co-occupancy matrix for HepG2 cells is *100**** × ****100* (47 TF families + 53 cofactors). The size of the matrix for K562 cells is *114**** × ****114* (42 TF families + 72 cofactors).

## Results

ChIP-seq experiments are used to map global binding sites of a given protein. A single ChIP-seq experiment is limited in resolution by the size of the generated DNA fragments typically several hundred base pairs long, thus defying the precise identification of the protein binding site. In this study we present analysis that identify collective TF binding sites in high resolution by utilizing the deep collection of more than 1000 TF ChIP-seq data in HepG2 and K562 cell, which helps to understand the mechanism underlying CREs associated with large number of TFs. The detailed data collection process is described in the Methods part. In brief, bed files of called peaks from the TF ChIP-seq experiments in HepG2 and K562 cells were downloaded from ENCODE portal [17]. After data filtering (to discard the data sets with < 2000 peaks, and those with high inconsistency between replicates. See Methods), we have 598 DNA binding proteins for HepG2 cell (545 TFs and 53 cofactors) and 409 DNA binding proteins from K562 cells (337 TFs and 72 cofactors). MNase-seq, RNA-seq, H3K4me3, H3k27ac and ATAC data for the two cell lines were downloaded from ENCODE and SRA. The complete information of data resources can be found in Supplementary Table S1. For convenience, we use an umbrella definition TF to refer to all DNA binding proteins.

### TF bindings collectively occur at relatively small number of genomic sites

We examined the distribution of the binding sites of all TFs, together with the gene expression data and epigenetic marker signals. Figure 1A shows a genome view at FOXA1 gene site in HepG2 cell with RNA-seq, H3K27ac signals and all the individual TF peaks lined up together. The vast majority of peaks overlap with other peaks. We plotted a histogram of the distance from each peak center to the nearest peak center (Supplementary Fig. S1A). 93.6% of peaks in HepG2 and 84.4% in K562 cells are less than 100 bp away from their nearest peak (center to center distance).

We selected the center 300 bp window from each peak and merged overlapping ones to obtain the merged peaks (MPs), using the merge function of Bedtools program [18]. We ranked the MPs based on the number of their overlapping individual TF peaks. Figure 1B shows the cumulative level of individual TF peaks along with the ranked MPs. In HepG2 cell, the ChIP-seq of 598 TFs summed up to a total of 8,154,853 peaks, resulting in 366,598 MPs. 80% of individual peaks are located within the top 28,790 most TF enriched MPs, which accounts for 7.86% of total MPs. In K562 cell, the ChIP-seq of 409 TFs summed up to a total of 4,671,922 peaks, resulting in 565,777 MPs. 80% of individual peaks are located within the top 81,162 most TF enriched sites, which is 14.3% of the total MPs. At cumulative level of 60% of total TF peaks, it is the top 26,350 most TF enriched ones that comprise 4.65% of the total MP in K562 cell. These results demonstrate that different TFs in cell nucleus tend to bind together, resulting in the observation that the vast majority of TFs are associated with only a relatively small number of genomic sites. The number of associated TFs at the top 30,000 most TF enriched MPs ranges from 44 to 591 in HepG2 cell and from 22 to 361 in K562 cell, and the median is 167 and 73 TFs, respectively (Supplementary Fig. S1B). Since some MPs contain multiple peaks from the same TF, the overlapping peak numbers at the MPs are even bigger (Supplementary Fig. S1C). Interestingly, TF binding is more focused in HepG2 cells compared to K562 cells, possibly due to the fact that liver cell (HepG2) is terminally differentiated but K562 is a multipotential cell line [26] that are potentially poised toward different gene expression patterns, thus has more regulatory sites.

We choose the top 30,000 most TF-enriched MPs in HepG2 cell (accounting for 81% of total TF binding events) and the top 50,000 most TF-enriched MPs in K562 cells (accounting for 73% of total TF binding events) for analysis of TF co-binding. These MPs are mostly promoter and enhancer elements due to their overlap with H3K27ac/H3K4me3/H3K4me1 epigenetic markers, and they are involved in the transcription of nearly all the active genes (Fig. 1C). We further mapped the MPs to the 18-state chromHMM annotations of HepG2 and K562 cells (downloaded from the ENCODE site) and the results are similar: about 10,000 MPs overlap with the TSS related annotations and the majority of the rest overlap with enhancer related annotations. A small number of them overlap with other annotations such as repressive polycomb markers (Supplemental Fig. 1D).

### Highly focused TF binding within TF-enriched MPs pinpointed to narrow regions mostly less than 200 bp

**Fig. 2:**
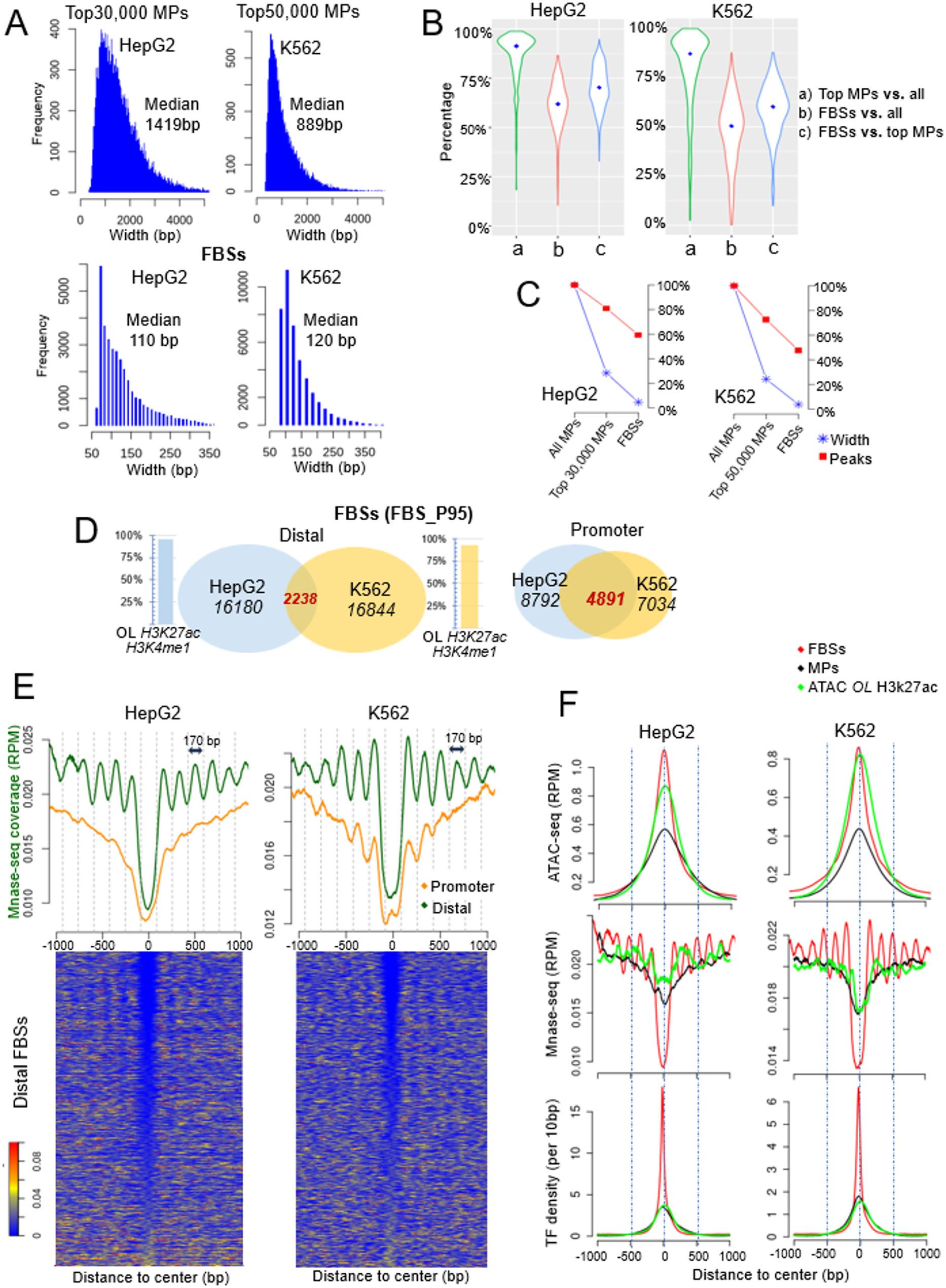
MNase-seq signals centered at FBSs reveal chromatin accessibility of distal CREs at single nucleosome resolution. **A** Top: Distribution of the width of the most TF-rich MPs (data is from the top 30,000 MPs in HepG2 and top 50,000 MPs in K562). Bottom: Distribution of widths of the FBSs. **B** For each TF, we calculated the ratio of, (a) the peaks located within the MPs to all its peaks; (b) the peaks located within the FBSs to all its peaks and (c) the peaks located within the FBSs to these within the MPs. **C** Plots of the decrease of the total occupied base pairs and total TF binding events, from all the MPs to the top TF-enriched MPs and to the FBSs (see text for detail). **D** The numbers of FBSs_P95 at promoter and distal regions, the overlap between HepG2 and K562 cells and the percentage of distal FBSs overlapping H3K27ac/H3K4me1 signals. **E** Top: Average nucleosome occupancy of the FBSs located at promoter (yellow) and distal regions (green). Bottom: heatmaps of all the distal FBSs. **F** Comparison of MNase-seq signals, TF density and ATAC-seq signals among three groups: FBSs (red), MPs with width below 2000 bp (black) and ATAC-seq peaks overlapping H3k27ac signals (green)

The MP sites are defined based on overlap of TF peaks and their widths range from hundreds to above three thousands of base pairs (Fig. 2A). We noticed that, for these MPs with deep coverage of individual TF peaks, the distribution of TF peaks is far from even, rather, they often concentrate in very narrow regions. Previous works showed that, although the position of the exact binding sites of a TF in a ChIP-seq peak might vary, the sites matching the TF binding motif tends to be well centered and have the maximal occurrence rate in the precise center of the ChIP-seq peaks [27, 28]. Therefore, we plotted the distribution of TF peaks within the MPs in this way: With each TF ChIP-seq peak represented by its center point, we scanned an MP in small bins (e.g.10 bp) for the number of the center points and plotted the numbers against the length of the MP. This generated very narrow peak(s), and the remaining part of the MPs have the value (number of the center points) close to zero, as shown in Fig. 1D. These narrow peaks indicate highly focused TF binding area.

We developed a pipeline to identify the focused TF binding sites (FBSs) from the top 30,000 most TF-enriched MPs in HepG2 and top 50,000 MPs in K562 cells (details in Methods part). In brief, the MPs were scanned for TF peak center points at non-overlapping 10 bp bins and consecutive bins with high TF counts were identified and combined, saved as candidate sites. The distribution of TF center point counts per unit length (lengths of 10–200 bp tested) in the MPs has a severely right-skewed long-tail shape (Fig S2B), with the right tail part representing highly focused TF binding regions. Based on the TF counts distributions profiled at unit lengths from 80 to 200 bps, we selected FBSs (of the corresponding length) whose counts fall into the right tail X percentile level. We made a sensitivity plot of the number of selected FBSs versus the threshold percentile (Fig S2C). Based on the observation that approximately each highly TF- bound CRE has one FBS and long CREs (e.g. above 1500 bps) can have two, we used the threshold percentile of 90% to define FBS (FBS_P90), which results in 33,200 FBSs in HepG2 and 42,235 in K562 cell.

The median widths of the FBSs are 110 bp and 120 bp in HepG2 and K562 cells, respectively, decreased from the median widths of 1419 bp and 889 bp for the MPs in the two cells (Fig. 2A). For about 90% of TFs in HepG2 and 70% of TFs in K562, more than half of their binding events within the MPs fall within the FBSs (Fig. 2B). If we take the sum of the base pairs of all the MPs as 100% and the total TF peaks also as 100%, The FBSs comprise 3.2% and 3.7% of total occupied base pairs but 56% and 48% of total binding events, in HepG2 and K562 cell respectively (Fig. 2C). The FBSs in both cells occupy about 0.2% of human genome.

For characterization of FBS features, we selected strong FBSs at a threshold percentile of 95% (termed as FBS_P95) for all subsequent analysis (*n* = 24972 in HepG2 and *n* = 23878 in K562), unless noted otherwise (coordinates of FBSs_P95 in Supplementary Table S2). Among these FBSs, 8792 and 7034 are located at promoter regions in HepG2 and K562 cells, respectively. For the FBSs at distal regions, 96% and 93% overlap with H3K27ac/H3K4me1 in HepG2 and K562 cells, respectively, indicating enhancer functions. The number of overlapping FBSs at promoter regions between HepG2 and K562 cells is 4891(~ 60%) while the that in distal regions between the two cells is only 2238 (~ 15%) (Fig. 2D), reflecting the cell type specificity of enhancers.

We further examined the spatial interactions between the distal FBSs and their contacts. From ENCODE site we downloaded the paired loop contacts results processed from Hi-C sequencing data of HepG2 and K562 cells (accession # in Table S1) and removed the contacts of a width above 2 kb. The distal FBSs were mapped to the looping contacts to identify their 3D contacts. The FBSs are highly enriched in 3D contacts compared to random genomic sites. The enrichment fold is 63 in HepG2 and 59 in K562 cells, respectively (p-value < 2.2e-16, binomial test) (Supplemental Fig. S2D). 5583 and 3603 unique promoter-distal FBS contacts were identified in HepG2 and K562 cells, respectively (Supplementary Table S3). These promoters are potential targets of the distal FBSs.

### The vast majority of the FBSs are nucleosome depleted

More than 90% of the FBSs have a width below 200 bp, which would correspond to the depletion of a single nucleosome. This prompted us to examine the nucleosome occupancy around these sites. We separated the FBSs into those within promoter regions, and those outside of promoter regions, i.e. distal gene regulatory sites, and plotted the average MNase-seq signals. The nucleosome occupancy of the two groups is distinct (top panel of Fig. 2E). At the distal FBSs, the center nucleosome free region is flanked by relatively well phased nucleosomes on both sides. By comparison, at promoter regions, the nucleosome occupancy at the FBSs is a deep valley but shows a gradual increase going towards the flanking regions, without well positioned nucleosome. Previous studies have shown that nucleosomes at promoters are positioned around TSS and are directional [29, 30], but the FBSs identified here are not always exactly at TSS and the plotting does not consider TSS orientation (Supplementary Fig. S2E). That is why we don’t observe well phased nucleosomes around the FBSs at promoter regions.

At the distal FBSs, the width of the deep valley of MNase-seq signal at the FBSs indicates single nucleosome depletion. To rule out the possibility that the results are caused by a small number of sites that do not represent the majority, we examined the MNase-seq signals at all the individual distal FBSs. As shown in the heatmaps (bottom panel of Fig. 2E), for most of the sites, the MNase-seq signal at the center is zero. Since MNase-seq experiments measure across a cell population and represent an ensemble average of millions of cells, the value of zero at these regions imply a constitutive single nucleosome depleted region, or a region with a very high nucleosome turnover rate where the nucleosome provides no protection from micrococcal nuclease digestion in the MNase-seq experiments.

The distal FBSs are almost all located at enhancers (Fig. 2D). The resolution of chromatin accessibility at enhancer sites is usually at the scale of ~ 500–1000 bp, determined by the peak width of ATAC-seq or Dnase-seq experiments (Fig. 2F). An ATAC-seq/DNase-seq peak denotes an overall nucleosome depleted region but does not identify precise nucleosome occupancy, which is a direct indicator of chromatin accessibility. Previous studies on HOT loci [6–8], which used nearly the same collection of TF ChIP-seq data from HepG2 and K562 cells as in this study, have never explored chromatin accessibility or TF binding within these loci. Figure 2F shows the MNase-seq signal and TF peak density based on the coordinates of three groups: (1) distal FBSs; (2) top 30,000 TF-enriched MPs non-overlapping promoters; (3) distal ATAC peak sites overlapping H3K27ac signal, which are commonly identified as enhancers. Only the MNase-seq plots centered at FBSs reveal nucleosome occupancy at single nucleosome resolution, corresponding with the peak of TF binding density. In other words, by identifying the FBSs we constructed high-resolution chromatin accessibility maps at distal CREs in HepG2 and K562 cells.

### Critical connection between focused binding of large number of TFs and nucleosome depleted status of DNA in vivo

**Fig. 3:**
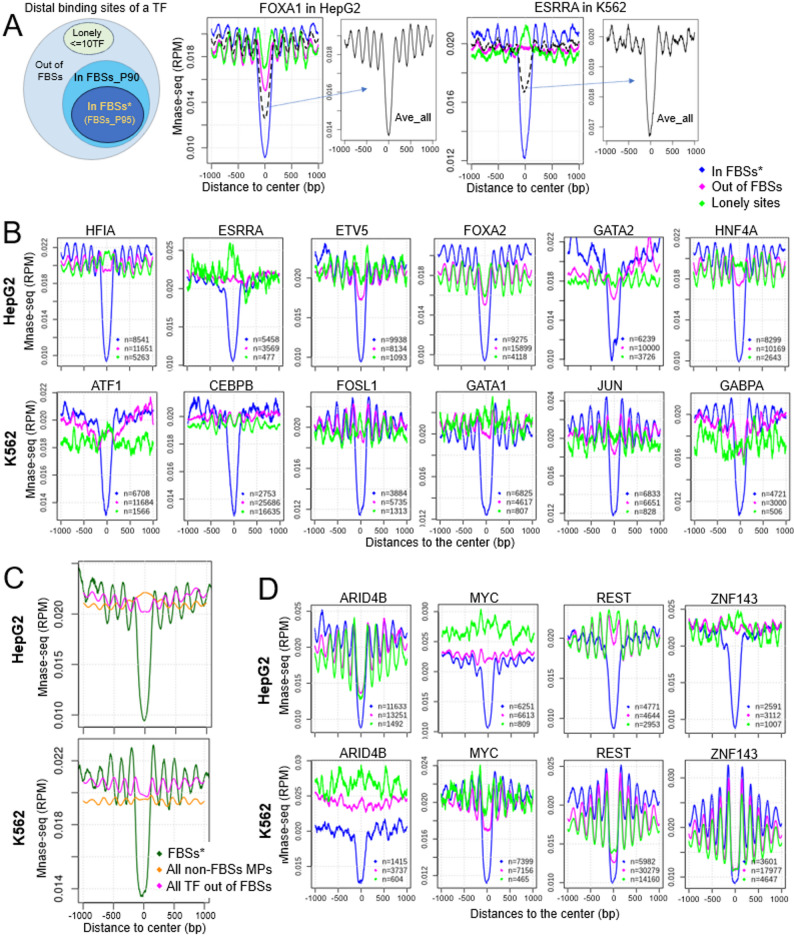
Nucleosome depleted status of DNA overwhelmingly occurs within FBSs. **A** Average MNase-seq signals of the binding sites of a TF were calculated for different groups: sites within the strong FBSs (FBS_P95), sites out of any FBSs (magenta), sites with less than 10 other TFs bound together (green) and the average of all distal binding sites (black). Shown are the results of FOXA1 in HepG2 cells and ESRRA in K562 cells. **B** Similar to panel A, without the black line, for more TFs. “n” indicates the number of peaks in each group. **C** Average MNase-seq signals at FBSs (dark green), all MPs not containing any FBSs (orange) and all TF binding sites out of FBSs (magenta). **D** Examples of TFs whose binding sites out of FBSs showing distinct nucleosome depletion patterns in HepG2 and K562 cells

Nucleosome depletion at TF binding sites is not a new concept. Previous studies have shown that TF binding sites are generally nucleosome depleted and flanked by well positioned nucleosomes [12, 31]. However, the results of these studies are the average of all the binding sites of individual TFs, without consideration of TF co-binding. Here, we showed that over half of TF binding events occur within FBSs (Fig. 2B). To examine how focused TF binding influences nucleosome depletion, we calculated the average MNase-seq signals at TF binding sites among different groups: (1) binding sites within the strong FBSs (FBSs_P95); (2) binding site outside FBSs; (3) those with less than 10 other TFs bound together (lonely sites). Sites at promoter regions are excluded from the analysis due to the special nucleosome orientation around TSSs. Figure 3A shows the MNase-seq signal of binding sites of FOXA1 in HepG2 cells and ESRRA in K562 cells from the above groups. Although the average signals of all distal binding sites of both TFs show a center valley with flanking phased peaks, the nucleosome depletion at the sites from different groups vary remarkably. This tells that the nucleosome depletion observed from the average MNase-seq signals of a TF are mainly contributed by its binding sites within the FBSs.

Next, for all the TFs we compared the MNase-seq signals at their binding sites within the strong FBSs to those out of any FBSs and to the lonely sites. There are 528 TFs in HepG2 and 276 TFs in K562 (804 TFs together) for which the plot of at least one of the groups shows a discernable pattern of nucleosome occupancy. The complete set of plots is in Supplementary Fig. S3, with a few examples shown in Fig. 3B&D. For almost all of these TFs, their binding sites outside of FBSs shows no nucleosome depletion (e.g. ESRRA in HepG2 and CEBPB in K562 cell) with a small number of TFs showing some depletion but remarkably less than that within the FBSs (e.g. ETV5 in HepG2; JUN in K562) (Fig. 3B & Supplementary Fig. S3). Only 23 TFs in HepG2 and 13 TFs in K562 (4.5% of the above 804 TFs) show strong nucleosome depletion outside of FBSs, such as ARID4B in HepG2 and JUND in K562 cells. Strong nucleosome depletion is defined as at least 50% of depletion compared to that at the FBSs (see Methods). Among these 36 TFs, ARID3A, CDC5L, DEAF1 and KLF16 in K562 cells show above 80% of nucleosome depletion compared to that at the FBSs (Supplementary Table S4).

For the vast majority of TFs, their binding sites with less than 10 binding partners (lonely sites) barely show any nucleosome depletion (Supplemental Fig. S3). The lonely sites of 21 TFs in HepG2 and 6 TFs in K562 (3.4% of the above 804 TFs) show strong nucleosome depletion, such as ARID4B in HepG2 and ZNF143 in K562 cells. Among these 29 TFs, GATAD1, MIXL1, SMAD3, TFDP2 and ZBTZ25 in HepG2 and ARID3A, KLF16 in K562 show above 80% of nucleosome depletion compared to that at the FBSs (Supplementary Table S4).

The above is the analysis of MNase-seq signal for each individual TF separately. We also plotted the average MNase-seq signal of all the TF binding sites outside of FBSs (*n* = 2500532 for HepG2; *n* = 2099979 for K562) including these within the top MPs, as well as all the MPs not containing an FBS (*n* = 328937 for HepG2 and *n* = 519636 for K562). As shown in Fig. 3C, TF binding sites out of FBSs overall show negligible nucleosome depletion, compared to that within FBSs. These results suggest that TF caused nucleosome depleted state of DNA in vivo typically depends on the highly focused binding of a large number of TFs. Lonely bound TFs, i.e. those bound with few other TF partners, even the TFs considered as pioneer factors such as GATA1 and FOXA1 [32, 33], are rarely capable of maintaining nucleosome free state of DNA in vivo.

We also observed that, for many TFs, the nucleosome occupancy patterns at their binding sites out of FBSs are distinct between HepG2 and K562 cells, i.e. cell type specific (Fig. 3D & Supplementary Fig. S3). Particularly, a few TFs appear to be able to keep nucleosomes displaced in one cell type but not in the other one. For example, the lonely binding sites of ARID4B and HMGXB4 show strong nucleosome depletion in HepG2 but not in K562 cells, while those of REST and ZNF143 are nucleosome depleted in K562 but not in HepG2 cells. The underlying mechanism is a potential future research topic.

### Strong sequence features in the focused TF binding sites

**Fig. 4:**
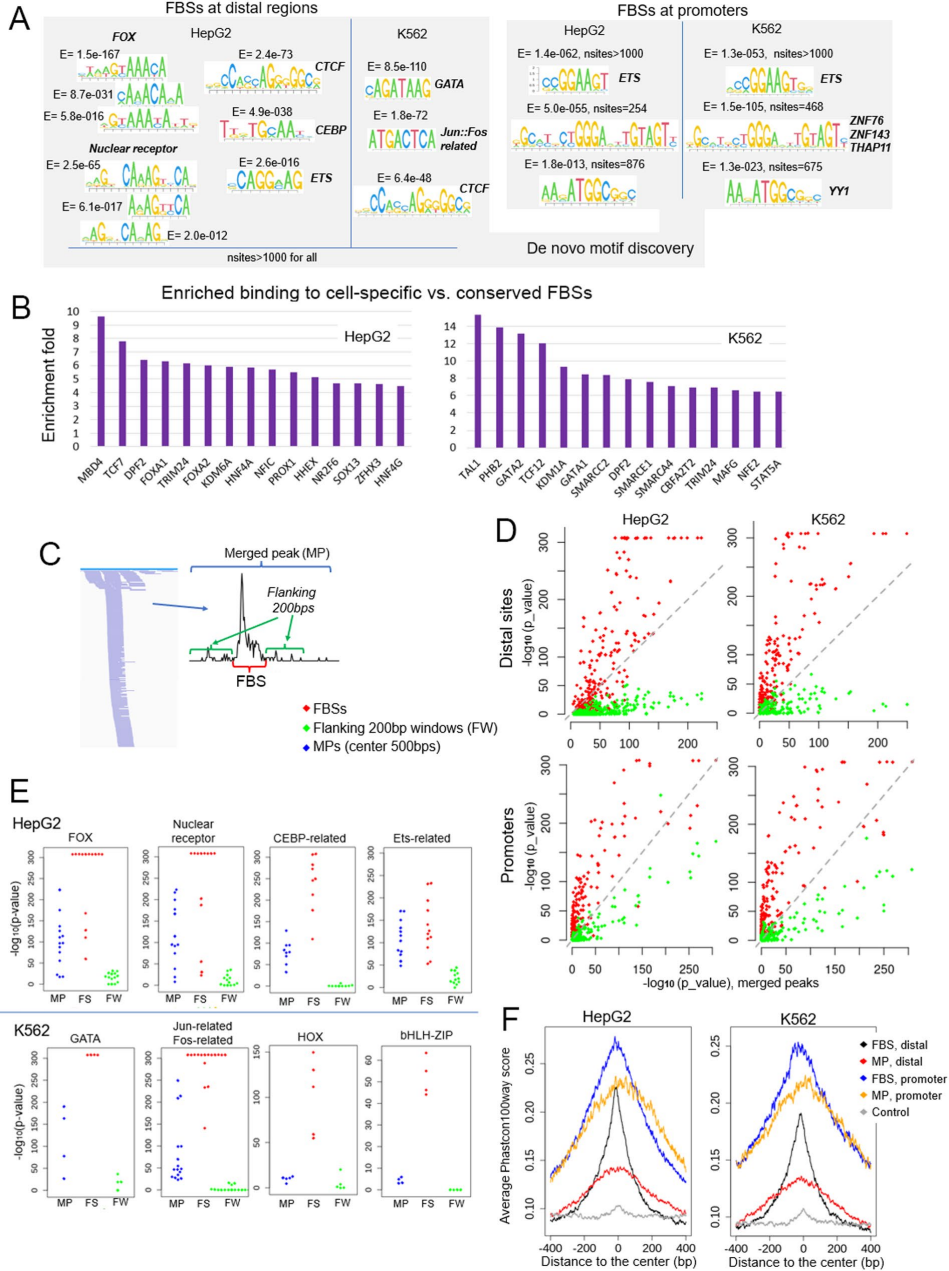
Strong sequence features of the FBSs. **A** Motifs identified in de novo motif discovery using MEME on the FBSs at distal and promoter regions. **B** The 15 TFs that have the strongest cell-specific vs. conserved FBS-associated enrichment. **C** Motif enrichment analysis searching JASPER database was performed for three groups of sites: MP sites (center 500 bp), FBSs (extended to 180 bp if shorter) and their immediate 200 bp flanking windows (FWs). **D** Comparison of p-values of the enriched TF motifs among the three groups. Each dot represents one motif. Red: FBSs vs. MPs. Green: FWs vs. MP. **E** A few example motif families with the corresponding enrichment significance in the 3 groups. **F** Average phastcon100 way score of the FBSs and top 30,000 most TF-enriched MPs at distal and promoter regions. The control group is randomly sampled 20,000 genomic sites that are bound by only one TF

We performed de novo motif discovery on the FBSs using MEME [23] with a threshold E-value of 0.001. For the FBSs at distal regulatory regions in HepG2 cell, the most significant ones identified by MEME are motifs of Forkhead (FOX) and Nuclear Receptor TF families. For the K562 distal FBSs, the most significant are the motifs of GATA and Jun/Fos related TF families (Fig. 4A, left panel). CTCF motif is among the most significant ones in both cells. The critical roles of Fork head and nuclear receptor TF families in liver cell functions and development have been investigated and proven in many previous studies [34–37]. GATA TF family, and Jun/Fos TF family, which belong to the complex AP-1, have also been shown to be critical regulators in hematopoietic process [38–41]. In HepG2 cell, motifs of the CEBP, and the ETS TF families were also identified, while for K562 no other significant motifs were found except for long and low-complexity motifs. Interestingly, the CTCF motif are present at 4621 distal FBSs in HepG2 and 3781 distal FBSs in K562 cells. Among these sites only ~ 55% of them are bound by CTCF protein, indicated by ChIP-seq data.

For the FBSs at promoter regions, besides long and low complexity motifs, the same three motifs were identified in both HepG2 and K562 cells. The first is a consensus motif of the ETS family. The other two are the motif of ZNF76, ZNF143 and THAP11 (the three TFs have highly similar binding motif) and that of YY1 (Fig. 4A, right panel). TFs from ETS families play important roles in many different biological processes and cellular functions [42–44]. ZNF143 and YY1 have fundamental functions in gene activation, cellular proliferation [45–47] and ZNF143 is also critically involved in chromatin looping [48, 49].

We next examined which TFs show enriched binding at cell-specific FBSs compared to conserved FBSs. Conserved FBSs are the ones overlapping between HepG2 and K562 cells and cell-specific FBSs are the non-overlapping ones. 33 TFs from HepG2 and 51 TFs in K562 show more than 3 folds enrichment in conserved versus cell-specific FBSs (*p* < 2.2e-16, binomial test). The 15 TFs with the highest enrichment are shown in Fig. 4B. These include TFs whose canonical binding motifs are the most enriched motifs identified from distal FBSs ( the vast majority of distal FBSs are cell-specific), such as FOXA1, FOXA2, HNF4A and NR2F6 in HepG2 and GATA1, GATA2 and MAFG in K562 cell.

### Motifs of essential cellular function related TFs are enriched at the distal FBSs, but not at the immediate flanking regions

De novo motif discovery might not identify short motifs that occur at low frequency. We also did the motif enrichment analysis searching the JASPAR2022 database [25] which include 841 human motifs. The analysis was performed with the JASPAR TFBS enrichment package (https://jaspar.genereg.net/enrichment/) that is based on the LOLA tool [50], for three groups of sites. The first group are the FBSs, extended to 180 bp if shorter, based on the reason that single nucleosome displaced region is about the length of 180 bp; the second group are the immediate 200 bp windows on both sides of the FBSs; the third are the center 500 bp windows of the top 30,000 most TF-enriched MP sites in each cell type (Fig. 4C). The analysis was performed separately for sites at the promoter regions and distal regulatory regions. Random genomic sites of sample size three times bigger with matching lengths and GC contents were used as control set for each group.

The FBSs at the distal regions are highly enriched with motifs of cell type specific and essential cellular function related TFs, among which are FOX, nuclear receptor, CEBP, POU domain families in HepG2 cell, and GATA, Fos/Jun/Maf related, CREB and ETS TF families in K562 cell (Supplementary Fig. S4). However, most of these motifs are not enriched at the immediate flanking 200 bp windows of the FBSs. When the analysis is performed for the center 500 bp regions of the merge peak sites, the identified enriched TF motifs are a subset of these obtained from the FBSs, but with larger p-values, i.e. lower statistical significance. Comparisons of p-values of all the enriched TF motifs among the three groups are shown in Fig. 4D&E.

As shown earlier in the de novo motif discovery, promoters and distal regulatory sites have distinct sequence features. For the FBSs at promoter regions, the most enriched motif families belong to ETS, Zinc finger, E2F related TF families, etc. (Supplementary Fig. S4). However, the highly enriched motifs are not as concentrated at the FBSs as at the distal regions – the flanking 200 bp windows also show significant enrichment (Fig. 4D, lower panel). This agrees with the results in Fig. 2E that the nucleosome free regions at promoters are wider and have lower nucleosome occupancy, which indicate longer DNA fragments for TFs to bind. The complete lists of the motif enrichment results are in Supplementary Table S5.

### Evolutionary conservation of the sequences at the FBSs

We examined the evolutionary conservation of the FBSs using phastCons100way scores, which are generated from alignment of 100 vertebrate species. For comparison, we calculated the average phastCons score of an 800 bp window flanking the center points of the FBSs, the MPs (those with width below 2000 bp), and a control group of randomly selected 20,000 genomic sites bound by only one TF. Since the FBSs at distal regions and promoters exhibit distinct features on enriched motifs and chromatin accessibility, we calculated their conservation scores separately. As shown in Fig. 4F, the sequence conservation is peaked at the center of the FBSs and remarkably highly than the middle area of the corresponding MPs in both distal and promoter groups. The sequence conservation of the regulatory sites at promoter regions are significantly higher than at distal regions, which agrees with a previous study that reported rapid evolution of enhancers compared to promoters [51].

### Strong enrichment of phenotypically causal variants at the FBSs within the MPs

Having established that the TF binding, chromatin accessibility and essential cellular function related motifs within MPs are focused on FBSs, we next examined whether phenotypically causal variants within MPs also tend to locate at the FBSs. We analyzed the variants enrichment in four groups of sites: (1) the MPs from which the FBSs are identified (the top 30,000 and the top 50,000 most TF-enriched MPs in HepG2 and K562, respectively); (2) the strong FBSs (FBSs_P95); (3) all FBSs (FBSs_P90); (4) regions within the MPs but outside of any FBSs. The enrichment fold was calculated against the probabilities (variant density) expected by chance (see Methods). Sites at promoter and distal regions were analyzed separately.

**Fig. 5:**
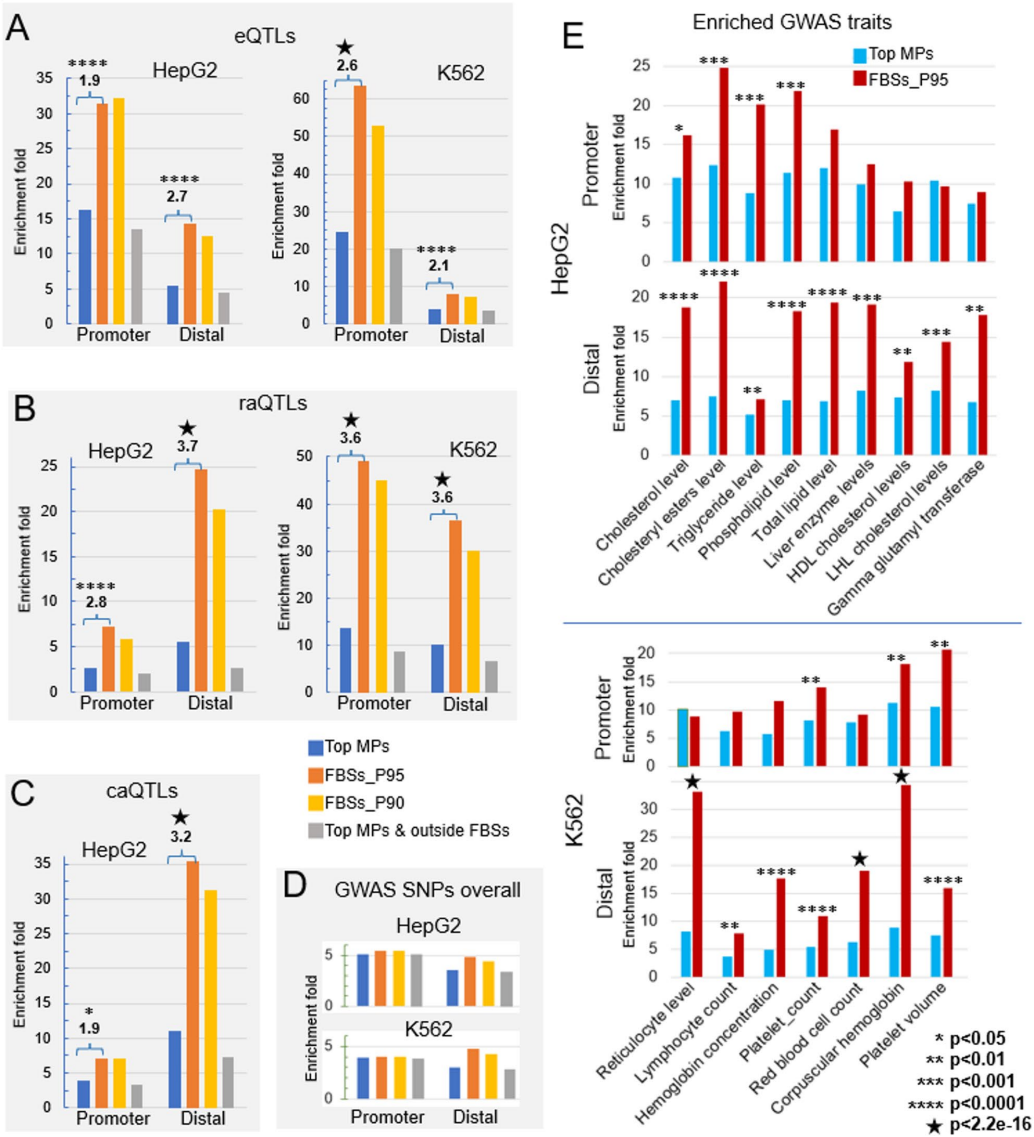
Enrichment of functional genetic variants at the FBSs. eQTLs (panel **A**), raQTLs (panel **B**), caQTLs (panel **C**) and GWAS SNPs overall (panel **D**) enrichment folds were computed for four groups of sites: The topmost TF-enriched MPs (30,000 in HepG2 and 50,000 in K562 cells) (blue), FBSs_P95 (orange), FBSs_P90 (yellow) and regions within the MPs but outside of FBSs (gray), compared to probabilities expected by chance. All groups are significantly enriched with the causal variants. The differences in the fold enrichment between the MPs and the FBSs are specified within each panel. **E**) The SNPs of GWAS traits that have the biggest enrichment fold in the FBSs compared with the MPs. The indicators of statistical significance level for the differences between FBSs and MPs are explained at the right bottom corner of the figure

We first analyzed the enrichment of eQTLs, which are the quantitative trait loci associated with expression level changes in mRNA. We download the most updated eQTL lists from GTExPortal (https://www.gtexportal.org/home/downloads/adult-gtex/qtl). We selected the “egenes” data sets in which each affected gene is linked to its most significant eQTL and filtered the eQTLs with a q value threshold of 0.05. The eQTL data set in liver was used for HepG2 cell and eQTLs of whole blood were used for K562 cell. Because there are different cell types in liver and eQTL in liver tissue might not function in HepG2 cell, we selected the eQTLs that the affected gene is expressed in HepG2 cell (with RPKM > 1), to get potentially the most relevant eQTLs. We filtered the eQTLs in whole blood for K562 cell with similar approach. As shown in Fig. 5A, the sites of all the four groups are enriched in eQTLs compared to probabilities expected by chance (p-value < 2.2e-16 for all, binomial test). Sites at promoter regions are more enriched with eQTLs, reflecting that sequences at promoter regions are more directly involved in gene expression regulation than at distal regulatory sites. Nevertheless, for both promoter and distal MPs the eQTLs are more concentrated at FBSs with the enrichment fold ranging from 1.9 to 2.7.

We next analyzed the QTLs identified through massively parallel reporter assays (raQTLs), which measured the direct effect of a genetic variant on reporter gene expression. We used the raQTLs reported in the study by Arensbergen et al., in HepG2 and K562 cells [52]. The sites of all the four groups are enriched in caQTLs compared to probabilities expected by chance (p-value < 2.2e-16 for all, binomial test). Compared to the results of eQTLs, the enrichment of raQTLs in FBSs within MPs are more pronounced, with the enrichment fold ranging from 2.8 to 3.7 (Fig. 5B).

We also analyzed chromatin accessibility QTLs (caQTLs), which are the variants that alter chromatin structure. A study by Currin et al. reported 3123 significant caQTLs in liver tissue [53]. We mapped these caQTLs to the MPs and FBSs in HepG2 cells and observed a similar enrichment pattern to that of raQTLs. The sites of all the four groups are enriched in caQTLs compared to probabilities by chance (p-value < 1e-5 for all the promoter sites and p-value < 2.2e-16 for all the distal sites, binomial test). FBSs are remarkably more enriched with caQTLs compared to MPs, especially in distal regions with an enrichment fold of 3.2 (Fig. 5C). Promoters are overall less enriched with caQTLs compared to distal regions. This might be because that promoter regions have more pronounced nucleosome depletion and so single variants play less a role in chromatin accessibility.

Finally, we analyzed the GWAS SNP enrichment. The enrichment of GWAS SNP overall in the above four groups of sites compared to probabilities by chance ranges from ~ 3–5 folds (p-value < 2.2e-16 for all, binomial test) (Fig. 5D), much lower than the previous 3 types of SNPs (mostly 10–50 folds more enriched). Moreover, the difference in enrichment fold between the MPs and the FBSs is not significant. We then examined SNPs of different GWAS traits separately. We found that the SNPs associated with some traits are greatly enriched in the regulatory sites of the two cells while most traits are not. Shown in Fig. 5E are the traits that have the greatest enrichment fold (ranging from ~ 1.5 to 4 times more enriched) in the FBSs compared with the MPs, at promoters, or distal regions or both. All of them are significantly enriched in the MPs/FBSs compared to probabilities by chance (p-value < 2.2e-16 for all, binomial test). These traits in HepG2 cells are all liver function related, such as triglyceride level and those in K562 cells are all immune system function related, such as hemoglobin concentration (Fig. 5E). These results imply that compared to rest of the regions in the merged peaks, the FBSs are more likely to be functionally relevant to the biology or genetic architecture underlying these traits, e.g. regulation of cholesterol level by hepatocytes. FBSs may harbor variants to affect TF binding, chromatin state or long-range regulatory interactions.

Combined, the enrichment of these various types of variants demonstrate that the functionally important residues in CREs tend to locate at FBSs. We further examine the conservation of the genetic variants-associated FBSs. We calculated the phastCons100way scores of the cell type specific GWAS traits-associated FBSs, eQTL-associated FBSs and raQTL-associated FBSs. The results showed that the GWAS traits-associated FBSs are significantly more conserved than the average FBSs, but eQTL-associated FBSs or raQTL-associated FBSs do not show remarkable increased conservation (Supplemental Fig. S5).

### High promiscuity of co-binding among all TFs and TF families at FBSs

TF co-binding can be defined in different ways. A strict definition is based on structural evidence of the direct or indirect interaction between TFs, which usually refers to pairwise TF co-binding such as TFs forming a complex before binding to DNA [13], or cooperative binding of two TFs facilitated through DNA without direct TF-TF interactions [15]. Another commonly used definition of TF co-binding or co-occupancy is based on the distance between the ChIP-seq peaks of the TFs: two peaks overlap or less than a defined distance (e.g. 100 bp or 2 kb) away from each other or, two peaks overlap with a sliding window of a defined length (e.g. 500 bp or 2 kb), or two peaks belong to a common MP [6–8, 54]. On this type of definition, the distance between the center of the two peaks can be anywhere between 0 to above 2 kb. In this study, we want to characterize the TF co-binding at FBSs, which are at single nucleosome length scale.

**Fig. 6:**
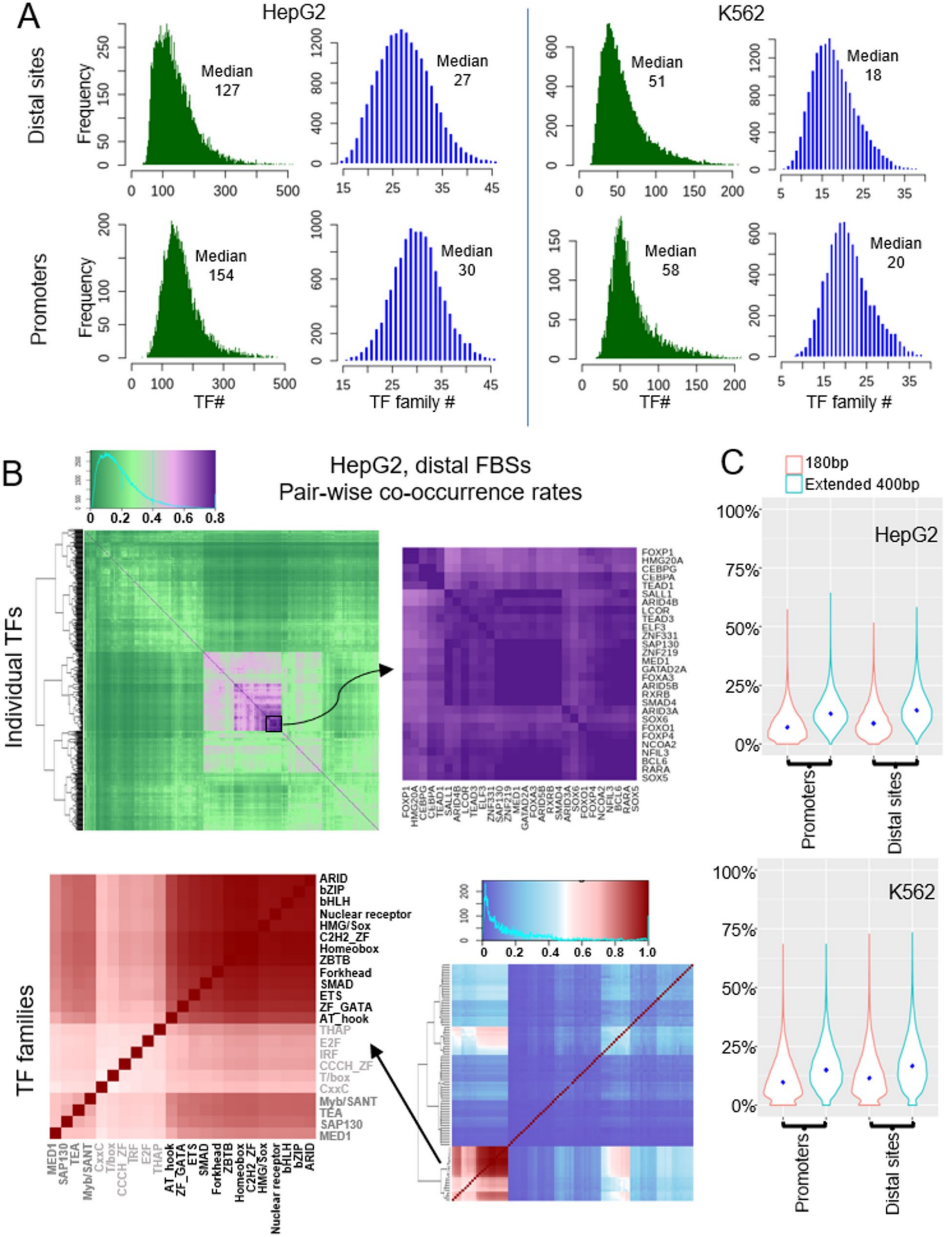
High promiscuity of co-binding among the majority of TFs and TF families. **A** Distribution of the number of TFs and TF families at the FBSs at distal and promoter regions in HepG2 and K562 cells. **B** Top: Co-occurrence rates among individual TFs at distal FBSs and a close-up graph of a group of 28 TFs with high co-bound rates. Bottom: Co-occupancy rates among all TF families and a close-up graph of 11 TF families that are present at nearly 100% of all distal FBSs in HepG2 cell. **C** The percentage of TFs that have their canomical motifs present on their associated FBSs

In contrast to the short lengths of the FBSs and the small number of highly enriched TF motif families identified above, ChIP-seq data shows that the number of TFs associated with individual FBS is large. We define a TF bound to a FBS as the center 10 bp of the TF peak overlapping at least 1 bp with the focused site (extended to 180 bp if shorter). The TFs analyzed in this study in HepG2 cell belong to 47 TF families and those in K562 cell belong to 42 families (Supplementary Fig. S6). There are on average more than 120 TFs from more than 25 TF families bound to an FBS in HepG2, and more than 50 TFs from more than 18 families bound to an FBS in K562 cell (Fig. 6A). Moreover, since the TFs included in this study are far from the complete list that is expressed in the cells, the actual number of co-bound TFs at these sites would be larger.

We examined the co-occurrence rates between any two TFs at the FBSs. The co-occurrence rate of TF A and B was calculated as N_***OL***_/Maximum (N_***A***_, N_***B***_), in which N_***OL***_ is the number of the overlapping sites between A and B and N_***A***_, N_***B***_ are the numbers of ChIP-seq peaks of TF A and B within the FBSs (see Methods). Sites at promoter and at distal regions were analyzed separately due to the preferences of some TFs for promoters or distal sites. TF group(s) with high level of co-binding among each other was identified with hierarchical clustering and presented with heatmaps (Fig. 6B & Supplementary Fig. S7). The co-occurrence rates among individual TFs are generally low, especially at promoter regions, where the majority of TFs have co-occurrence rates below 40%. At distal FBSs, a small number of TFs have high co-binding rates of ~ 70–90% among each other. In HepG2 cell, there are 28 TFs forming a densely co-bound group, including FOXP1, HMG20A, CEBPG, CEBPA, etc. (Fig. 6B upper panel) In K562 cell distal sites, our clustering identified two highly co-bound TF groups with the co-bound rates ~ 70–90%(Supplementary Fig. S7B).

We also examined co-occupancy rates among any two TF families, which was calculated as the percentage of FBSs at which both TF families are present. We found that, a few families are present at all the FBSs (Fig. 6B lower panel & Supplementary Fig. S7). In HepG2 cell, TFs from C2R2_ZF, Homeobox, bHLH, ZBTB and bZIP families are present at 100% of all promoter and distal FBSs. Moreover, at distal sites, TFs from ARID, Fork head, nuclear receptor, ETS, GATA families also co-occur at high rates (> 80%) with each other. In K562 cell, TFs from C2H2_ZF, bZIP and bHLH families co-occur at nearly 100% rate at all FBSs. In addition, TFs from ARID, Myb/SANT and HMG/Sox families co-occur at high rates (> 70%) with each other at distal sites.

These results demonstrate that for the majority of TFs, there are no specific co-binding patterns. However, since there are typically large number of individual TFs from dozen(s) of TF families co-localized within the same focused site (Fig. 6A), these results combined demonstrate the high promiscuity of co-binding among all the TF and TF families. We next examined how many bound TFs have their motifs present at the binding sites. We first selected all the TFs in this study that have motif(s) in the JASPA2022 database (some TFs do not have a motif in the database), which are 211 TFs in HepG2 and 176 TFs in K562 cells. Among these TF pools, we searched each FBS (extended to 180 bp if shorter) for the motifs present and compared to the bound TFs (based on ChIP-seq data). Motif search is performed in Bioconductor with in TFBSTools package against JASPAR 2022 motif database [25] with a threshold p-value 0.0001. For the majority of FBSs, less than 25% of associated TFs have their motifs present at the associated FBSs (Fig. 6C). We extended the TF search window at both sides to 400 bp and only see a slight increase in the number of the motifs present.

### Short DNA resident time of TFs might underlie the requirement of a large number of TFs for sustained nucleosome depletion in vivo

**Fig. 7:**
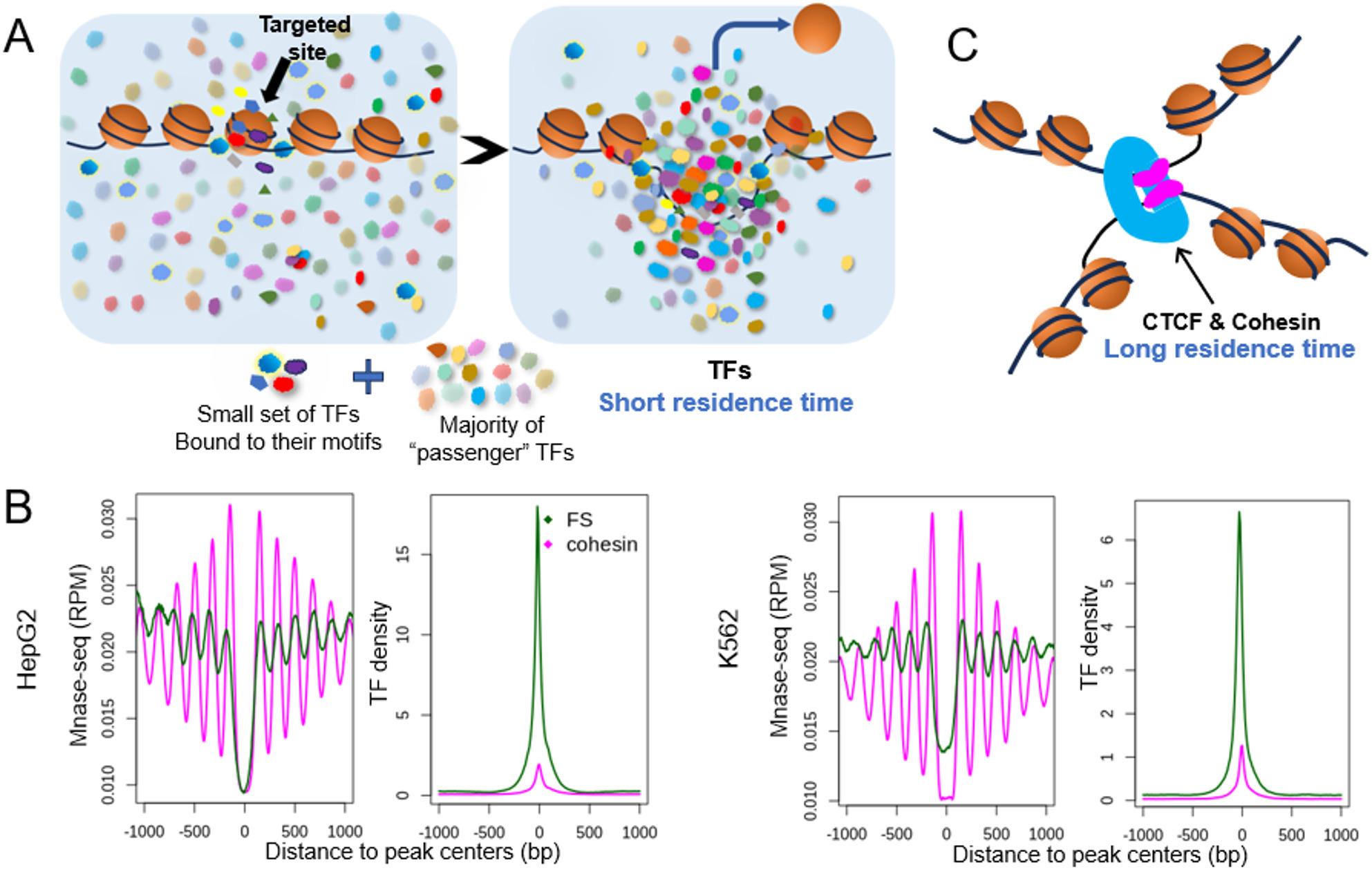
Short DNA residence time of most TFs might underlie the requirement of a large number of TFs for sustained nucleosome depletion. **A** Diagram showing a general mode of cooperative binding of large number of TFs at distal regulatory sites. Highly clustered TF binding is both the result and necessary condition of nucleosome depletion at CREs. **B** MNase-seq signals and TF binding centered at enhancer FBSs and insulators (Cohesin/CTCF binding sties). **C** Diagram showing the stable binding of Cohesin/CTCF complex, in contrast to the transient binding of most TFs

There are approximately 1000 TFs expressed in a human cell. The mechanism of collective binding of this large number of TFs in their natural cellular environment has rarely been explored. Based on the results in this study, we propose a general model of coordinated co-binding of a large number of TFs in vivo as depicted in Fig. 7A: TF binding in cell nucleus is highly concentrated on single nucleosome depleted regions within CREs. The focused binding features both specificity and non-selectivity. Specificity is the motif-directed binding of a small set of TFs, which plays essential role in initiating the nucleosome eviction. Non-selectivity or promiscuity is demonstrated by the large number of TFs associated with each FBS that do not contain their canonical motifs, as well as the diversity of families of these TFs. These TFs might bind to sub-optimal motifs, or bind with no sequence selectivity, or could be recruited through protein-protein interactions. Indeed, recent studies have demonstrated multiple types of non-canonical binding [55], including the binding based on DNA shape recognition [56], G-quadruplex secondary structures [57] and even direct interaction between DNA and intrinsically disordered regions of TFs [58]. These non-canonically associated TFs can be considered as “passenger” TFs.

In this model, the absence of a nucleosome creates a microenvironment for the non-canonical binding of passenger TFs and vice versa, dynamic binding of large number of TFs prevents nucleosome binding. In Fig. 3 we showed that, nucleosome depleted state of DNA typically depends on the focused TF binding – because for more than 90% of the TFs, their binding sites outside of the FBSs shows little or no nucleosome depletion (Fig. 3 & S3). The key factor for this dependence, we reason, could lie in the several magnitudes of difference in DNA residence time between TFs and nucleosomes.

Chromatin accessibility is dynamically regulated through competitive interplay between DNA binding proteins and nucleosomes. Nucleosome turnover rate at active chromatin is estimated to be about 30 min to 2 h [11, 59], whereas the residence time of the majority of TFs is in the range of seconds [11, 60]. A few TFs (e.g. pioneer factors) might be able to displace a nucleosome, however, due to the TFs’ short DNA residence time the nucleosome is very likely to retake the place again. Therefore, the dynamic binding of a large number of TFs are required to keep the DNA nucleosome free, as the situation of the FBSs.

For proteins with long DNA residence time, like cohesin and CTCF bound at insulators, once they bind to DNA they prevent the binding of other proteins. CTCF has an average residence time of 29 min [61] and cohesin’s residence time at interphase of the cell cycle can be as long as a few hours [62, 63]. As shown in Fig. 7B, the extent of nucleosome depletion is comparable at the center of enhancer FBSs and cohesin/CTCF sites, but the latter are not accessible to TFs (indicated by low TF density) due to the long residence time of cohesin/CTCF. Moreover, in contrast to FBSs, cohesin/CTCF create highly stable nucleosomes in the flanking region, due to their stable binding (Fig. 7C).

## Discussion

Previous studies by Ramaker et al., Moyer et al. and Hudaiberdiev & Ovcharenko [6–8] used TF ChIP-seq data sets that mostly overlap with the data used in this study. These studies have reported that HOT loci mainly overlap promoters and enhancers and, they have the features of these regulatory elements, such as predictive of nearby gene expression, driven by sequence features, enriched in 3D chromatin contact points, etc. These studies treated HOT sites as whole merged TF peaks, mostly 1–2 kb long (equivalent the DNA lengths for 5–12 nucleosomes) bound by a large number of TFs. However, the formation of HOT loci remains an unanswered question from the perspective of chromatin accessibility, as elaborated in the introduction part. In the current study, the FBSs in HepG2 and K562 cells overlap with 85–92% of the identified HOT loci from these three studies, while the top 30,000 most TF-enriched MPs in this study include 100% of these HOT loci. Yet FBS is conceptually new, in that FBS is the single nucleosome depleted region within a HOT site, as the focal point of chromatin accessibility, TF binding, essential motif and functional genetic variants enrichment. It reveals the critical connection between sustained nucleosome depletion and the focused binding of a large number of TFs in vivo, which helps resolve the paradox of HOT loci versus nucleosome occupancy. The new findings in this study extend our knowledge in two different yet related research areas: The first is how nucleosome depleted regions (NDRs) are maintained at CREs (how TFs keep a nucleosome displaced) and the second is how TF binding sites are specified in vivo.

It is generally considered that NDRs are created by TFs through a multi-step process. First, TFs gain access to histone-​bound DNA and bind to their preferable motifs by exploiting short periods of DNA accessibility during nucleosome turnovers [11]. Alternatively, pioneer TFs bind to nucleosomal DNA and initiate nucleosome depletion. The next step is the recruitment of DNA-remodeling proteins to establish the nucleosome depleted or open chromatin state. We showed in this study that nucleosome depleted states almost exclusively exist at the FBSs where highly focused binding of a large number of TFs occur. TF binding sites outside of FBSs barely show nucleosome depletion, including many TFs considered as pioneer factors. Our results tell that a small number of DNA-binding proteins are rarely able to maintain a nucleosome depleted state of DNA (even if they can displace a nucleosome) and the focused binding of large numbers of TFs is a necessary requirement to sustain an NDR. This expands our current understanding about the mechanism of how TF mediated chromatin remodeling is achieved in vivo. The correlation between NDR and focused TF binding can be explained by the differences of DNA residence times between TFs and nucleosomes as shown in Fig. 7.

On the other side, FBSs attract most TFs without having their canonical binding motifs, which strongly argues for the critical role of nucleosome depleted DNA in TF binding. It has been challenging to determine how TF binding locations are specified in vivo. Here our results suggest that, besides a small number of TFs that bind through motif recognition, the majority of TFs are associated with their binding sites with no or low selectivity primarily due to the absence of nucleosome barrier. This type of “passenger” binding by a large number of TFs is in contrast to the motif-direct binding of a few TFs that primarily determines sequence specificity of the binding sites. Motif-direct binding of multiple TFs can be independent of each other like in the well accepted billboard model [3] or, mediated through DNA and frequently have a composite co-binding motif markedly different from the individual TF’s motifs, demonstrated by the in vitro CAP-SELEX analysis [15]. An in vivo example is the heterotypic interdependence of TBX5 and NKX2-5 in cardiogenesis shown in the work by Luna-Zurita et al. [14].

The current work is focused on the physical nature of co-binding of a large number of TFs in vivo. Many questions remain regarding the coordinated actions among different TFs in chromatin accessibility remodeling and gene regulation. For example, the nucleosome occupancy patterns around many TFs are cell type specific. One of these is ZNF143 that shows great nucleosome depletion around all its binding sites in K562. A recent study reported that ZNF143 has long DNA residence time of more than 20 min [64], much longer than most TFs. We thought this could be the reason that the lonely binding site of ZNF143 exhibit strong nucleosome depletion in K562. Yet, in HepG2 cells the binding sites of ZNF143 outside of FBSs show no nucleosome depletion at all. This could be related to the cell type specific TF co-binding. Different binding partners for the same TF could lead to different conformation of the protein complex, or the recruitment of different set of cofactors, which lead to distinct consequences of chromatin accessibility remodeling.

The FBS identification is based on ChIP-seq data from the ENCODE database. Though generated under standardized guidelines, data from ENCODE can still be affected by biases such as antibody quality and lab-specific artifacts. Antibody-dependent biases may lead to off-target binding or failure to detect true protein–DNA interactions. Lab-specific biases resulting from different experimental conditions like crosslinking, chromatin fragmentation or library preparation also introduce data discrepancies. As we examined the data from different labs, we did find remarkable differences in called ChIP-seq peaks for some TFs. Though we removed data sets or data points whose reproducibility is questionable, the TF binding sites and peak intensity are still subject to systematic biases. We believe that the basic pattern of TF binding we observed in this study, i.e. FBSs, reflects true biological mechanisms. Yet, some specific quantifications such as extent and intensity of TF enrichment of each FBS call for further validation as more data, perhaps from more advanced techniques, become available in the future.

## Conclusions

In this study, we pinpointed the essential functional and sequence features to the single nucleosome depleted regions, within CREs hundreds to thousands of bps long. We also found that the nucleosome depleted status of DNA in vivo usually requires focused binding of a large number of TFs. We propose that the several magnitudes of difference in DNA residence time between most TFs and nucleosomes might underlie this requirement. The study reveals a detailed map of chromatin accessibility at distal CREs in the two human cells and improves our understanding of how chromatin accessibility is regulated through the interplay between TFs and nucleosomes in vivo.

## Supplementary Information

Supplementary Material 1.

Supplementary Material 2.

Supplementary Material 3.

Supplementary Material 4.

Supplementary Material 5.

Supplementary Material 6.

## Data Availability

The resources of all the public data used in the manuscript and the major results of this study are available in the supplementary data.
