# Supplementary material for: High-resolution map of chromatin accessibility - insights into the focused binding of a large number of transcription factors
Source: Epigenetics Chromatin. 2026 Feb 22;19:14. doi: 10.1186/s13072-026-00665-2 (PMC13032593; doi:10.1186/s13072-026-00665-2)
Supplement: Supplementary file 1 — Supplementary Material 1. [file 13072_2026_665_MOESM1_ESM.pdf]

Supplementary Figures

Note*: Supplementary Fig. 3 is placed at the last due to its large size (51 pages)

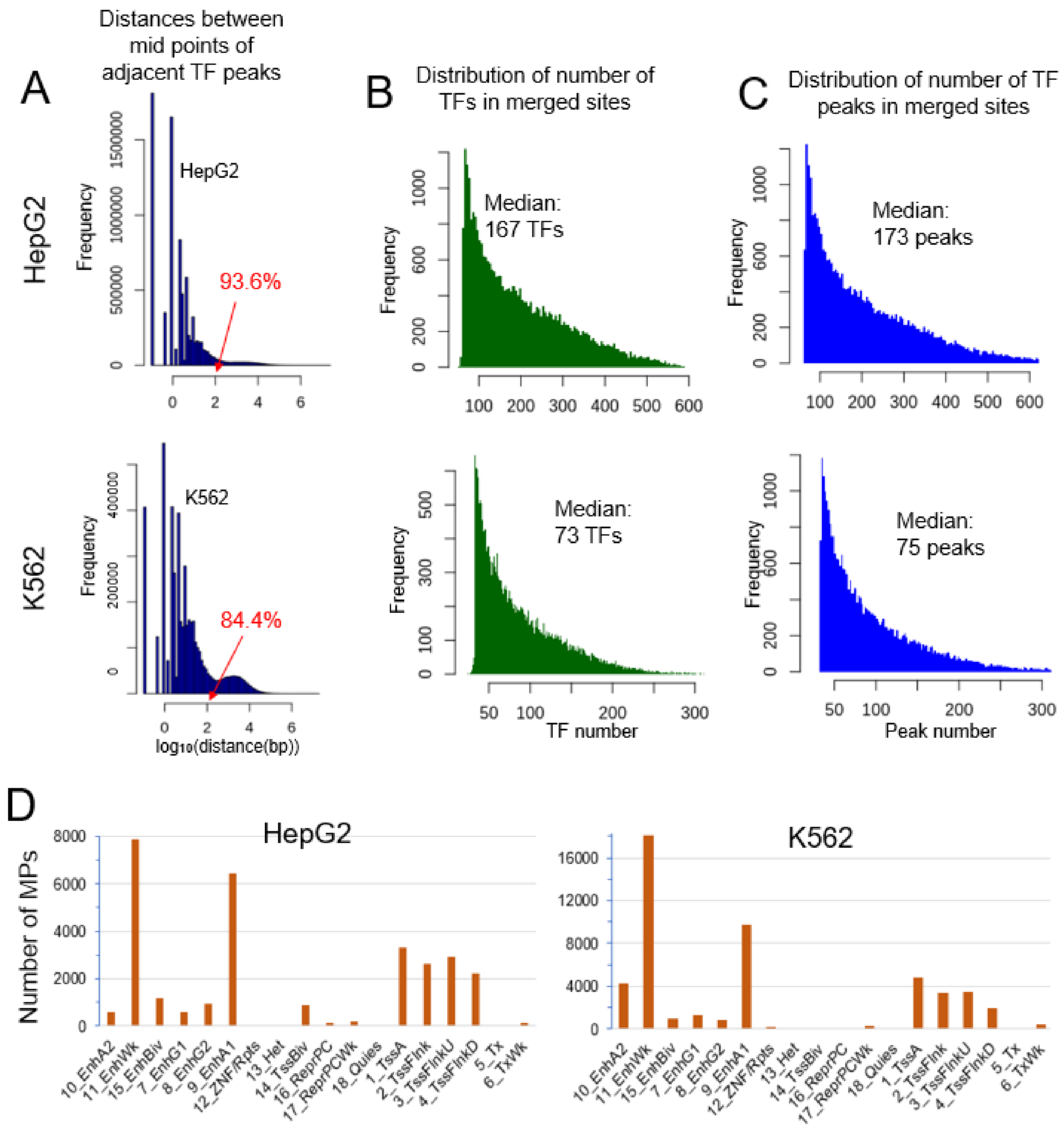

Figure S1: Metrics of individual TF peak adjacency and the merged peaks in HepG2 cells and K562 cells. A) Distribution of the distances of each TF peak to its nearest peak. The distance is center to center distance. If the centers of two peaks overlap, we use 0.1 bp as distance, to get a valid value for log10(distance). B) The distribution of numbers of TFs associated with each of the top 30,000 most TF-enriched merged peaks. C) The distribution of numbers of TF peaks associated with each of the top 30,000 most TF-enriched merged peaks. D) Mapping of the most TF-enriched MPs (the top 30,000 in HepG2 and the top 50,000 in K562) to the annotated 18 chromatin states computed with HMM model.

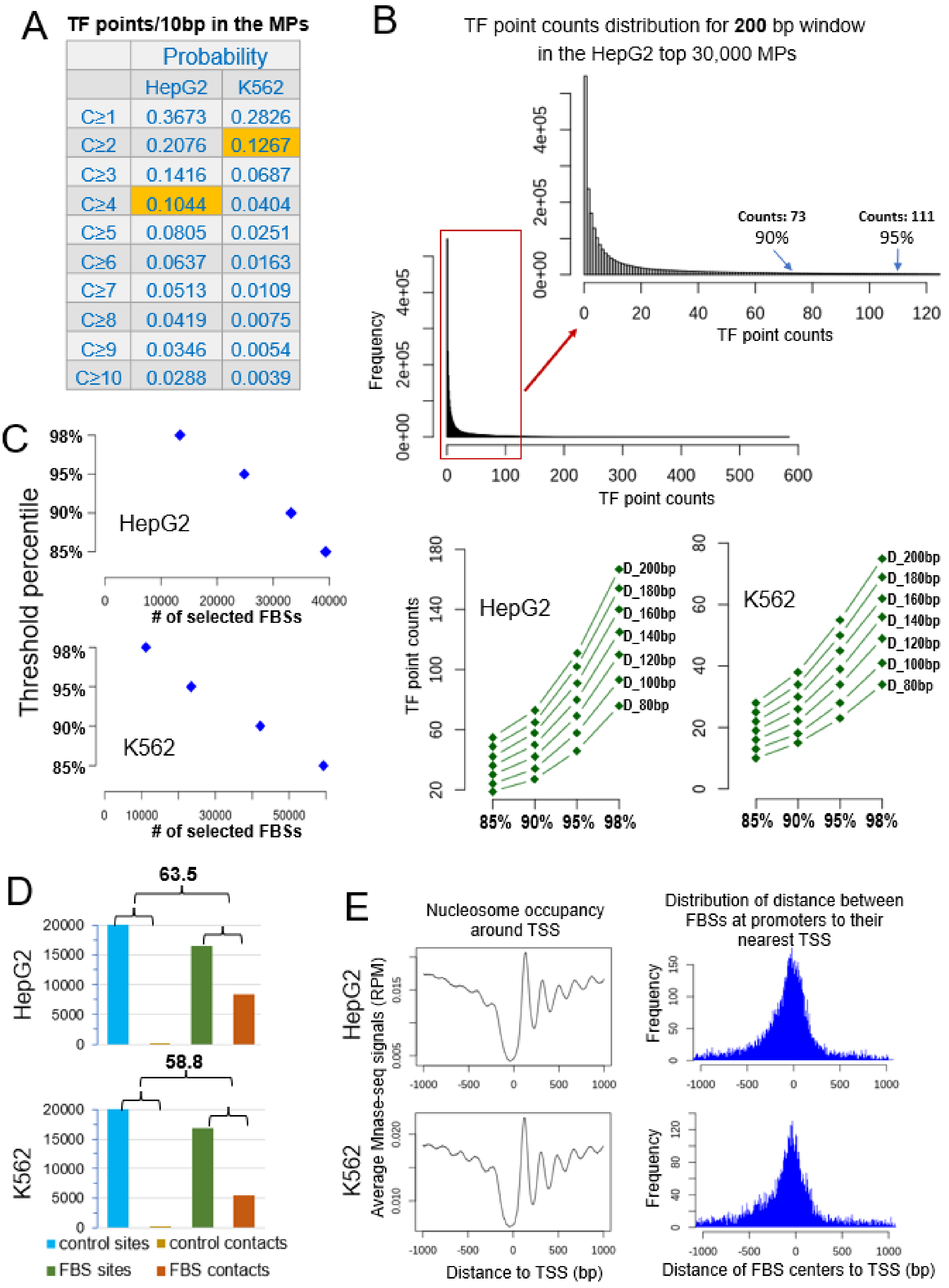

	Probability	
	HepG2	K562
C≥1	0.3673	0.2826
C≥2	0.2076	0.1267
C≥3	0.1416	0.0687
C≥4	0.1044	0.0404
C≥5	0.0805	0.0251
C≥6	0.0637	0.0163
C≥7	0.0513	0.0109
C≥8	0.0419	0.0075
C≥9	0.0346	0.0054
C≥10	0.0288	0.0039

Figure S2: FBS identification from the top merged peaks (MPs). A) Probability table of different TF point counts (per 10 bp) in the MPs. B) Top panel: The distribution of TF point counts per unit length (shown is 200bp unit) in the MPs has an extreme right skewed long-tail shape, with the right tail representing the focused TF binding sites. Bottom panel: The distribution was profiled for unit lengths of 80, 100, 120, 140, 160, 180 and 200 bps and the counts at right tail 85%, 90%, 95% and 98% level are shown for each distribution. C) The number of total FBS selected at the four threshold percentile levels. D) Enrichment of 3D chromatin contacts in the distal FBSs compared to random genomic sites. E) Left panel: Nucleosome occupancy related to transcription start sites (TSSs) of active genes. Right panel: Positions of FBSs (center points) at promoter region in relation to the TSSs. Negative values indicate upstream of TSS and positive values downstream of TSSs. E) Enrichment of 3D loop contacts among distal FBSs compared to random genomic sites.

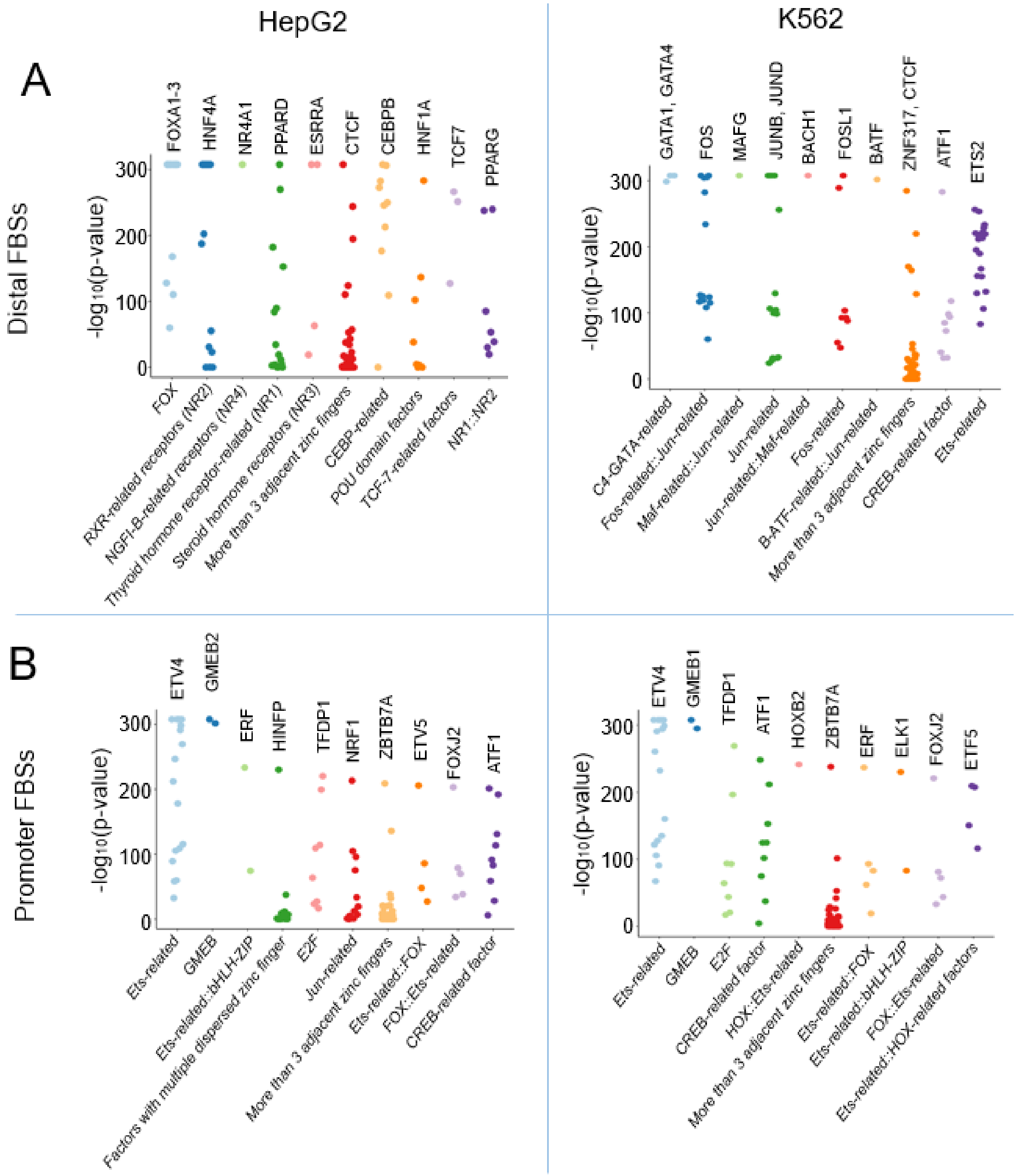

Figure S4: The 10 most enriched TF families identified from the distal focused sites in HepG2 and K562 cells using JASPAR enrichment analysis. Representative TF(s) are shown on the top, which are highly expressed and have been proven to be critical for cellular functions. A) The FBSs at distal regions. B) The FBSs at promoter regions.

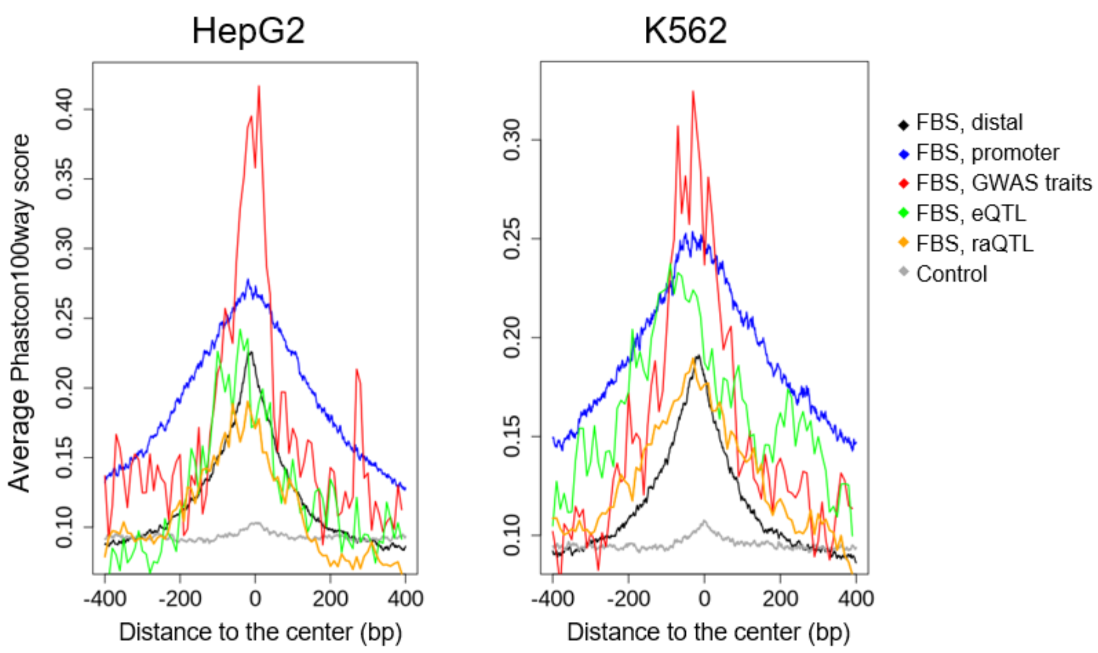

Figure S5: Comparison of the sequence conservation of different groups of FBSs. Average phastcon100 way score of the GWAS traits SNP associated FBSs, eQTL associated FBSs, raQTL associated FBSs and distal and promoter FBSs. The GWAS traits SNPs, eQTLs and raQTLs are from the analysis results of Figure 5. The control group consists of randomly sampled 20,000 genomic sites that are bound by only one TF.

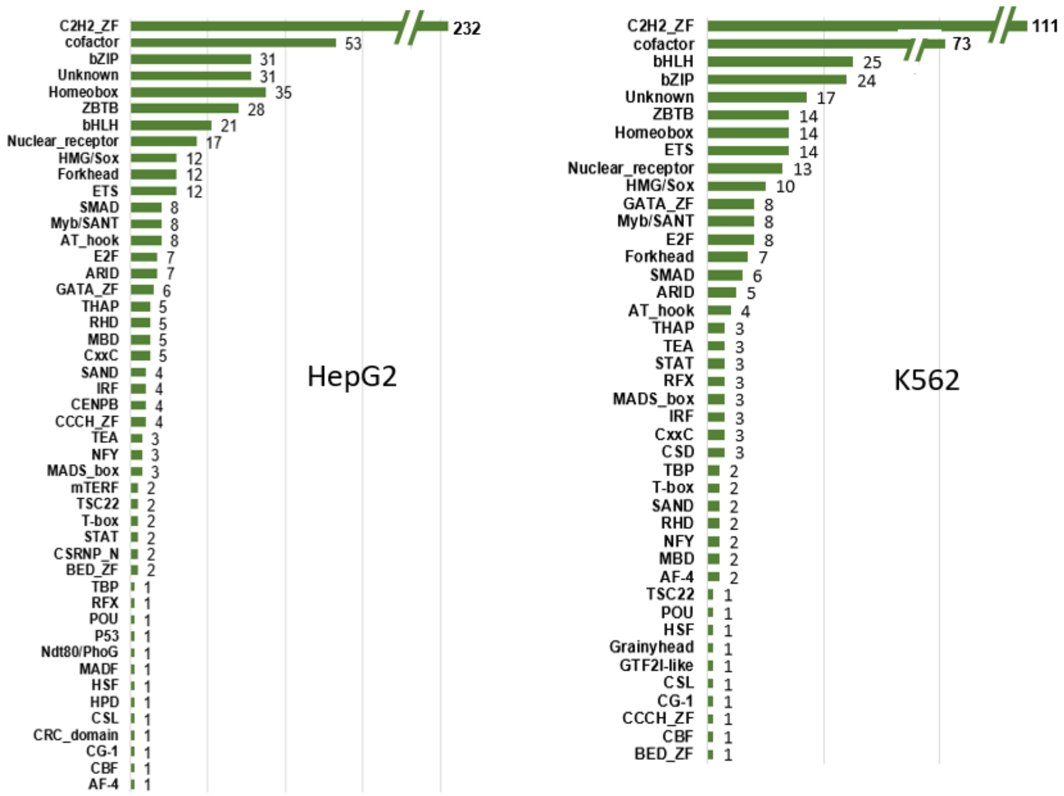

Figure S6: For all the TFs analyzed in this study, the number of TFs that belong to different TF families, in the HepG2 and K562 cells. TF family information, and whether a DNA binding protein (DAP) is a TF or a cofactor, are obtained through three different resources: JASPAR database (PMID 34850907), human TF database from https://humantfs.ccbr.utoronto.ca/ (PMID 29425488) and HumanTFDB 4.0 (PMID 36268869). More than 95% of TFs are categorized to the same family across the three resources. For those with inconsistent TF family curations we manually assigned the TF to the family from one of the curations. In this way, the 545 non-cofactor TFs in HepG2 cell are assigned to 47 families. The 337 non-cofactor TFs in K562 cells are assigned to 42 families.

HepG2, pair-wise co-occurrence rates at promoter FBSs

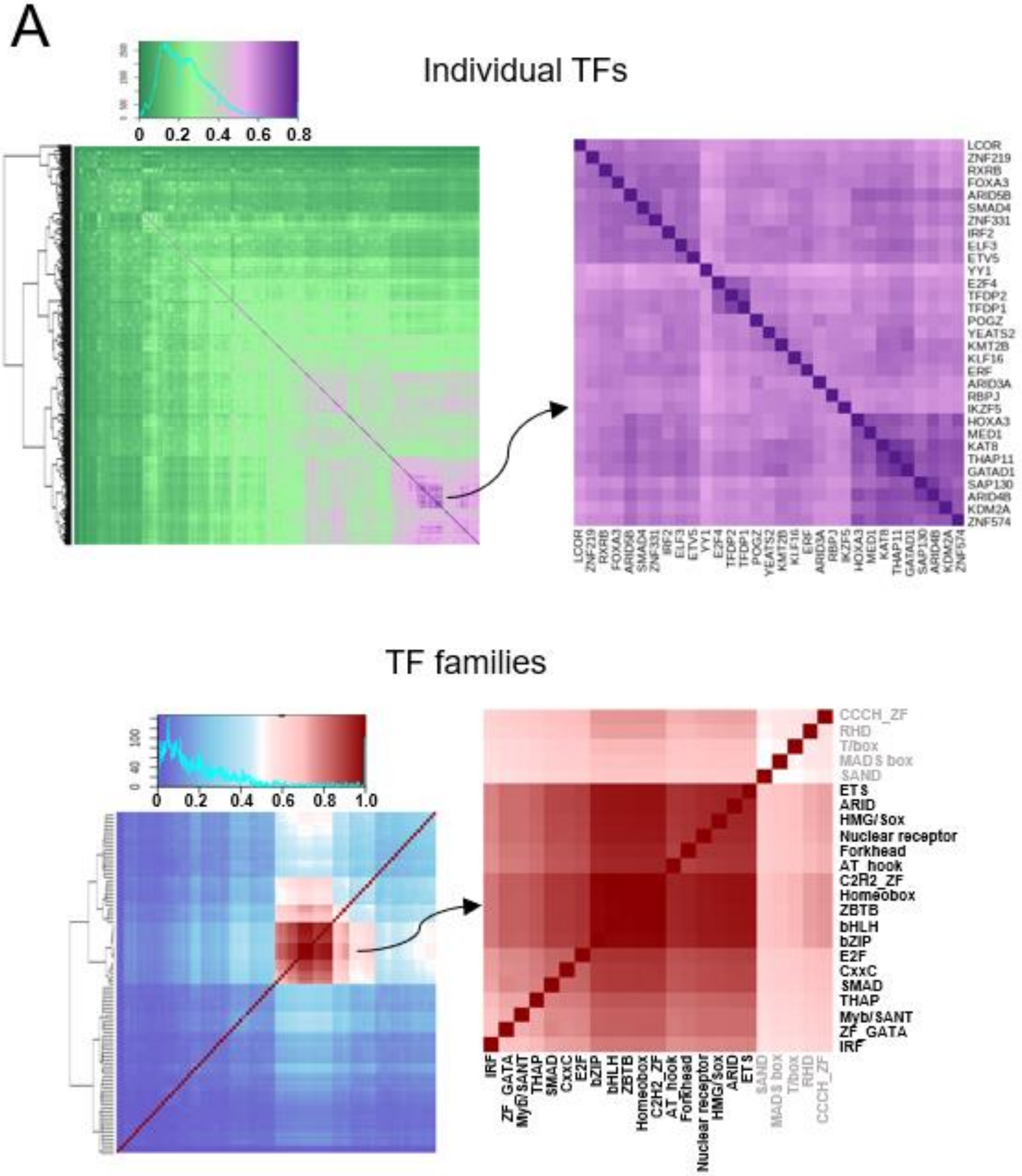

Figure S7A

K562, pair-wise co-occurrence rates at distal FBSs

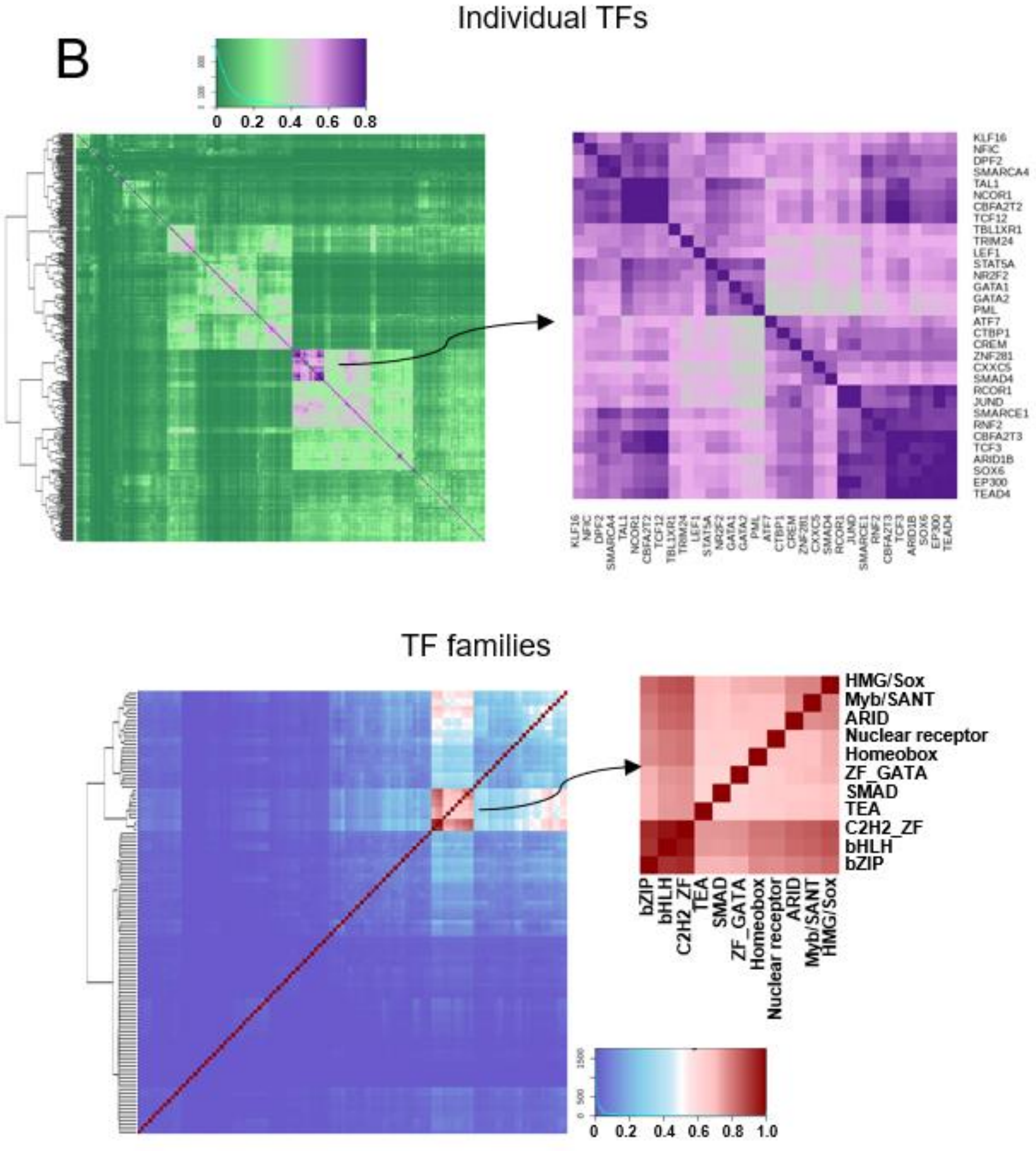

Figure S7B

K562, pair-wise co-occurrence rates at promoter FBSs

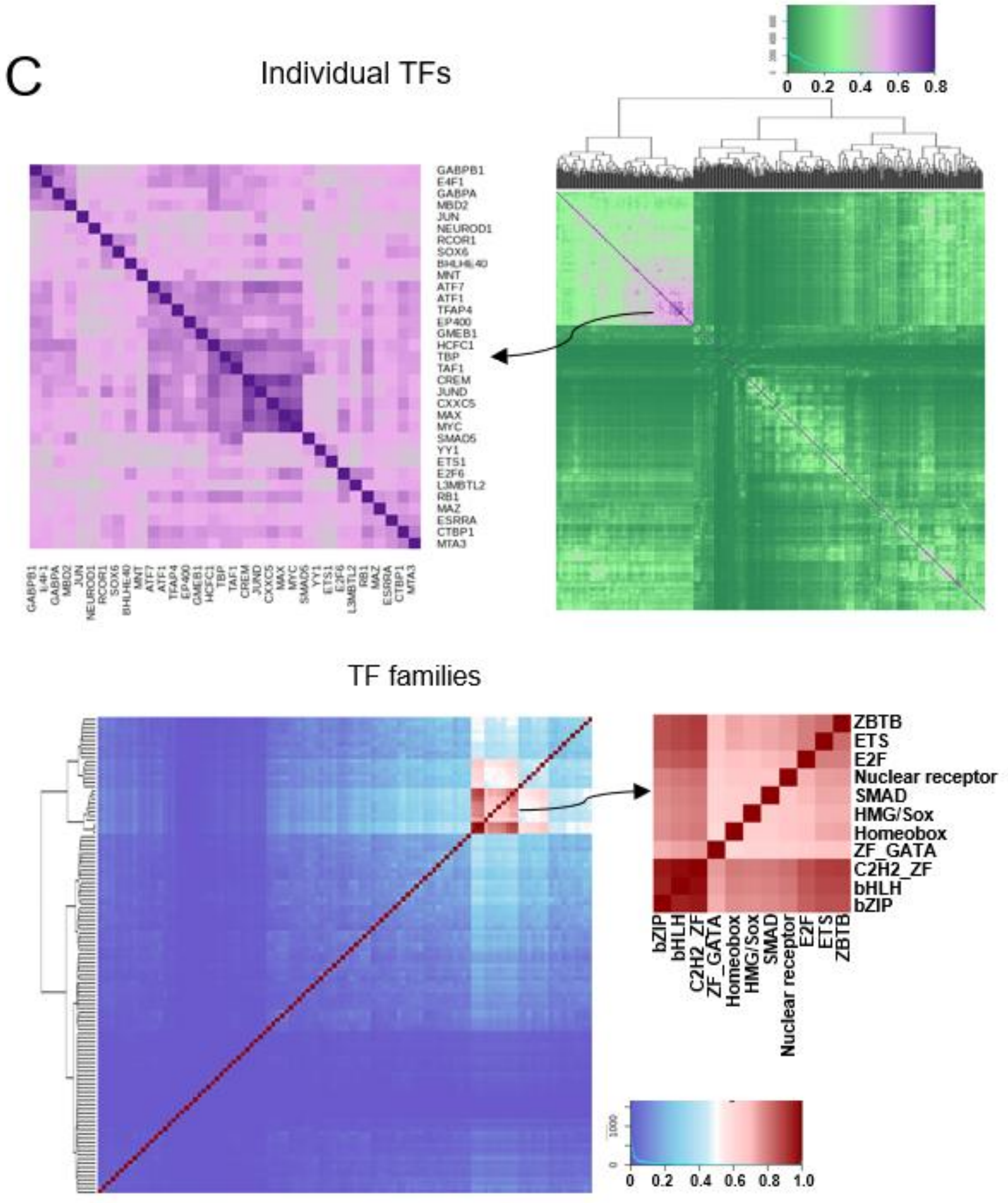

Figure S7C

Figure S7: Co-occurrence rates between any two TFs and any two TF families at FBSs at distal regions and promoter regions. A) The co-occurrence rates at promoter FBSs in HepG2 cells. B) The co-occurrence rates at distal FBSs in K562 cells. C) The co-occurrence rates at promoter FBSs in K562 cells. Top panel: individual TFs including cofactors. Bottom panel: TF families/cofactors (see Methods for details).

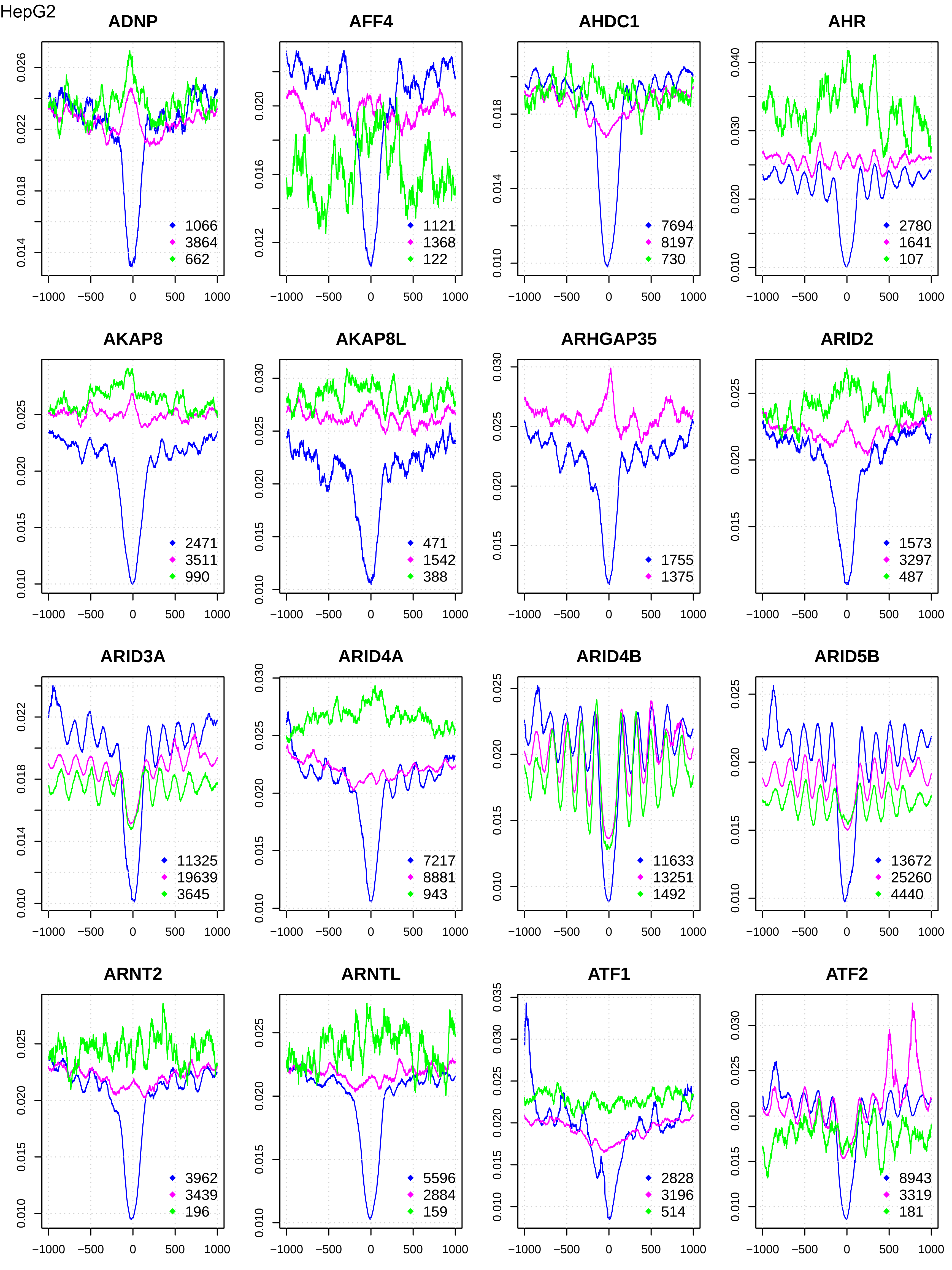
HepG2
ADNP
1066
3864
662
AFF4
1121
1368
122
AHDC1
7694
8197
730
AHR
2780
1641
107
AKAP8
2471
3511
990
AKAP8L
471
1542
388
ARHGAP35
1755
1375
ARID2
1573
3297
487
ARID3A
11325
19639
3645
ARID4A
7217
8881
943
ARID4B
11633
13251
1492
ARID5B
13672
25260
4440
ARNT2
3962
3439
196
ARNTL
5596
2884
159
ATF1
2828
3196
514
ATF2
8943
3319
181

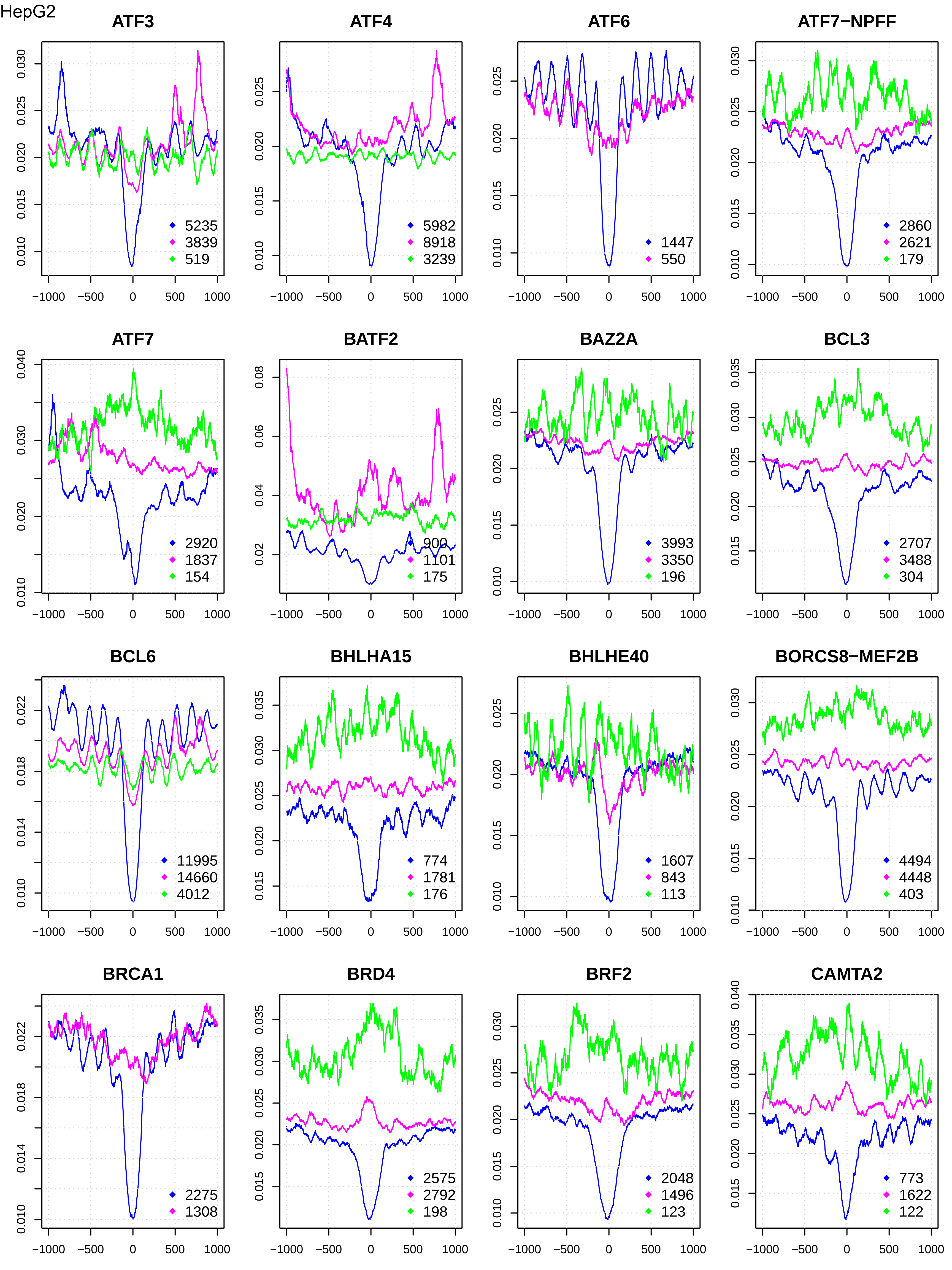
HepG2
ATF3
5235
3839
519
ATF4
5982
8918
3239
ATF6
1447
550
ATF7−NPFF
2860
2621
179
ATF7
2920
1837
154
BATF2
900
1101
175
BAZ2A
3993
3350
196
BCL3
2707
3488
304
BCL6
11995
14660
4012
BHLHA15
774
1781
176
BHLHE40
1607
843
113
BORCS8−MEF2B
4494
4448
403
BRCA1
2275
1308
BRD4
2575
2792
198
BRF2
2048
1496
123
CAMTA2
773
1622
122

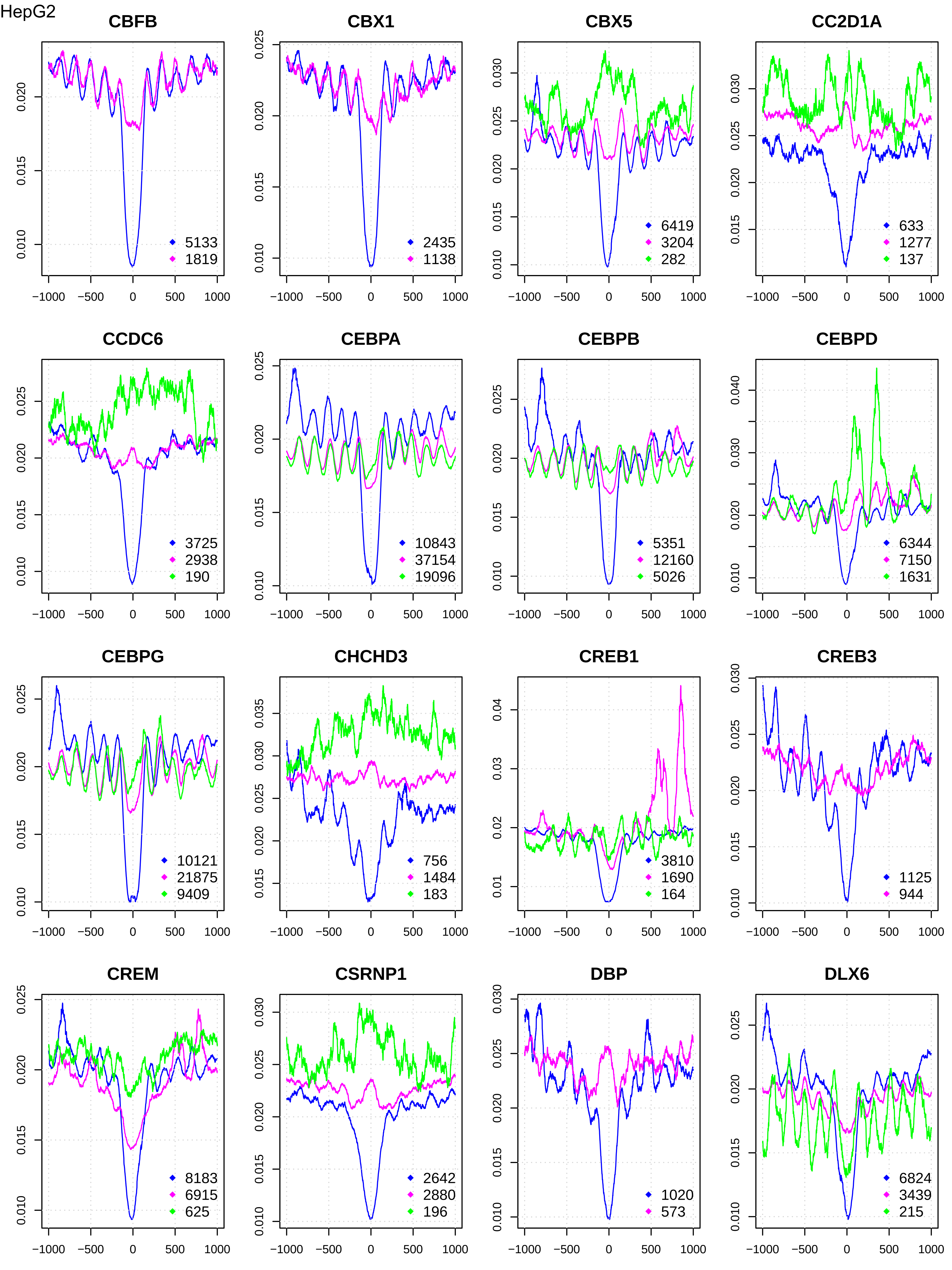
HepG2
CBFB
5133
1819
CBX1
2435
1138
CBX5
6419
3204
282
CC2D1A
633
1277
137
CCDC6
3725
2938
190
CEBPA
10843
37154
19096
CEBPB
5351
12160
5026
CEBPD
6344
7150
1631
CEBPG
10121
21875
9409
CHCHD3
756
1484
183
CREB1
3810
1690
164
CREB3
1125
944
CREM
8183
6915
625
CSRNP1
2642
2880
196
DBP
1020
573
DLX6
6824
3439
215

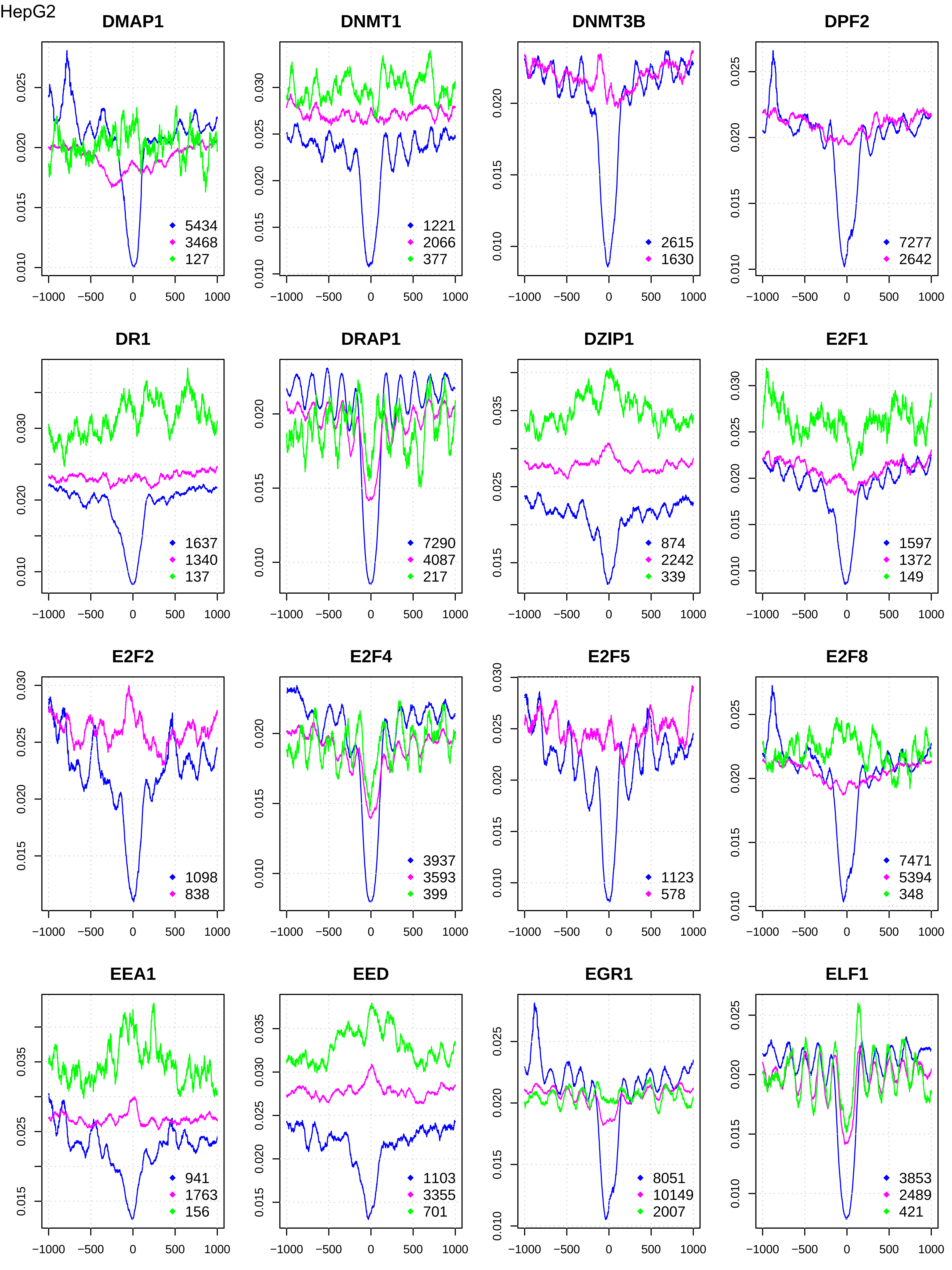
HepG2
DMAP1
5434
3468
127
DNMT1
1221
2066
377
DNMT3B
2615
1630
DPF2
7277
2642
DR1
1637
1340
137
DRAP1
7290
4087
217
DZIP1
874
2242
339
E2F1
1597
1372
149
E2F2
1098
838
E2F4
3937
3593
399
E2F5
1123
578
E2F8
7471
5394
348
EEA1
941
1763
156
EED
1103
3355
701
EGR1
8051
10149
2007
ELF1
3853
2489
421

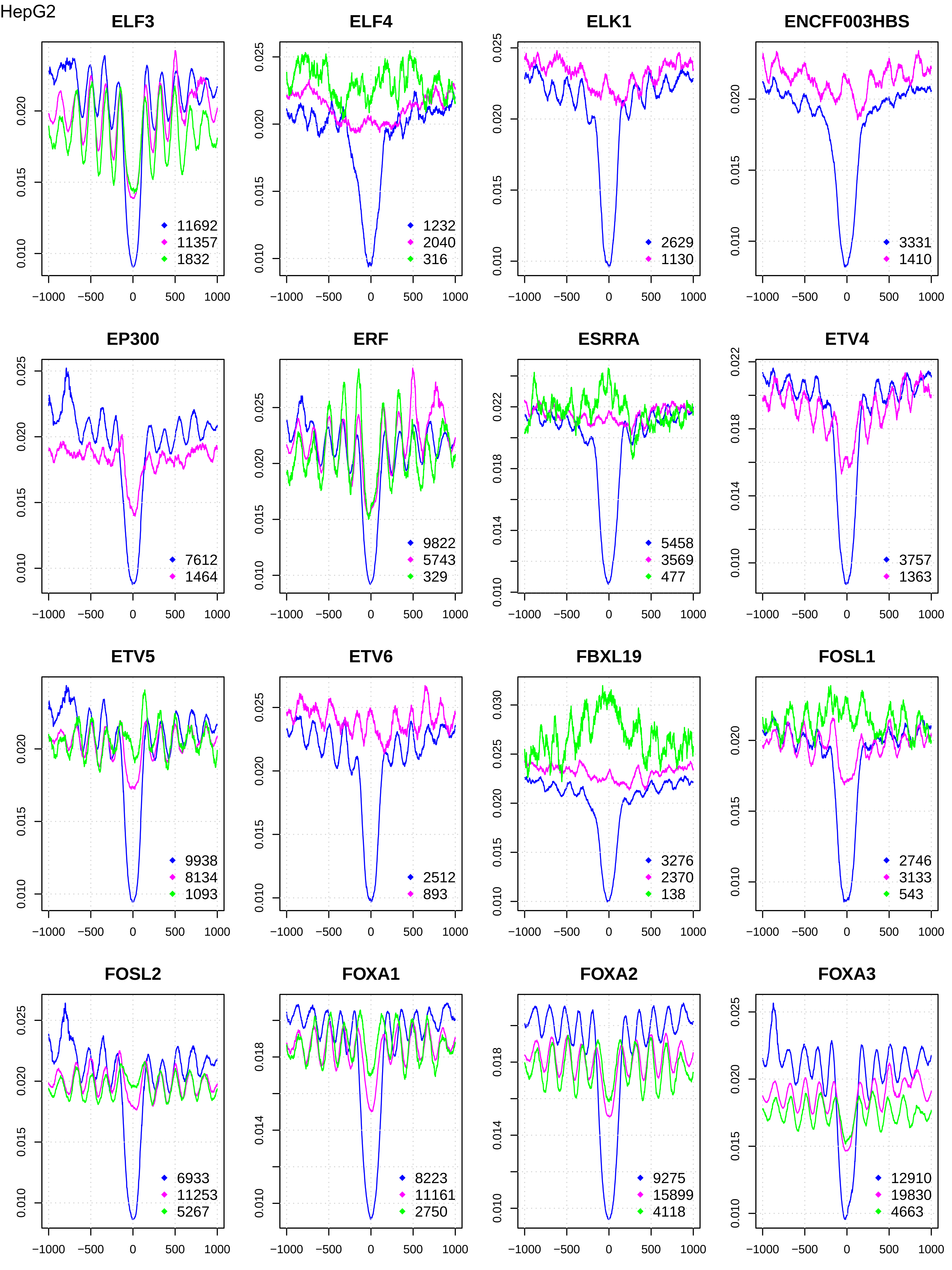
HepG2
ELF3
11692
11357
1832
ELF4
1232
2040
316
ELK1
2629
1130
ENCFF003HBS
3331
1410
EP300
7612
1464
ERF
9822
5743
329
ESRRA
5458
3569
477
ETV4
3757
1363
ETV5
9938
8134
1093
ETV6
2512
893
FBXL19
3276
2370
138
FOSL1
2746
3133
543
FOSL2
6933
11253
5267
FOXA1
8223
11161
2750
FOXA2
9275
15899
4118
FOXA3
12910
19830
4663

HepG2

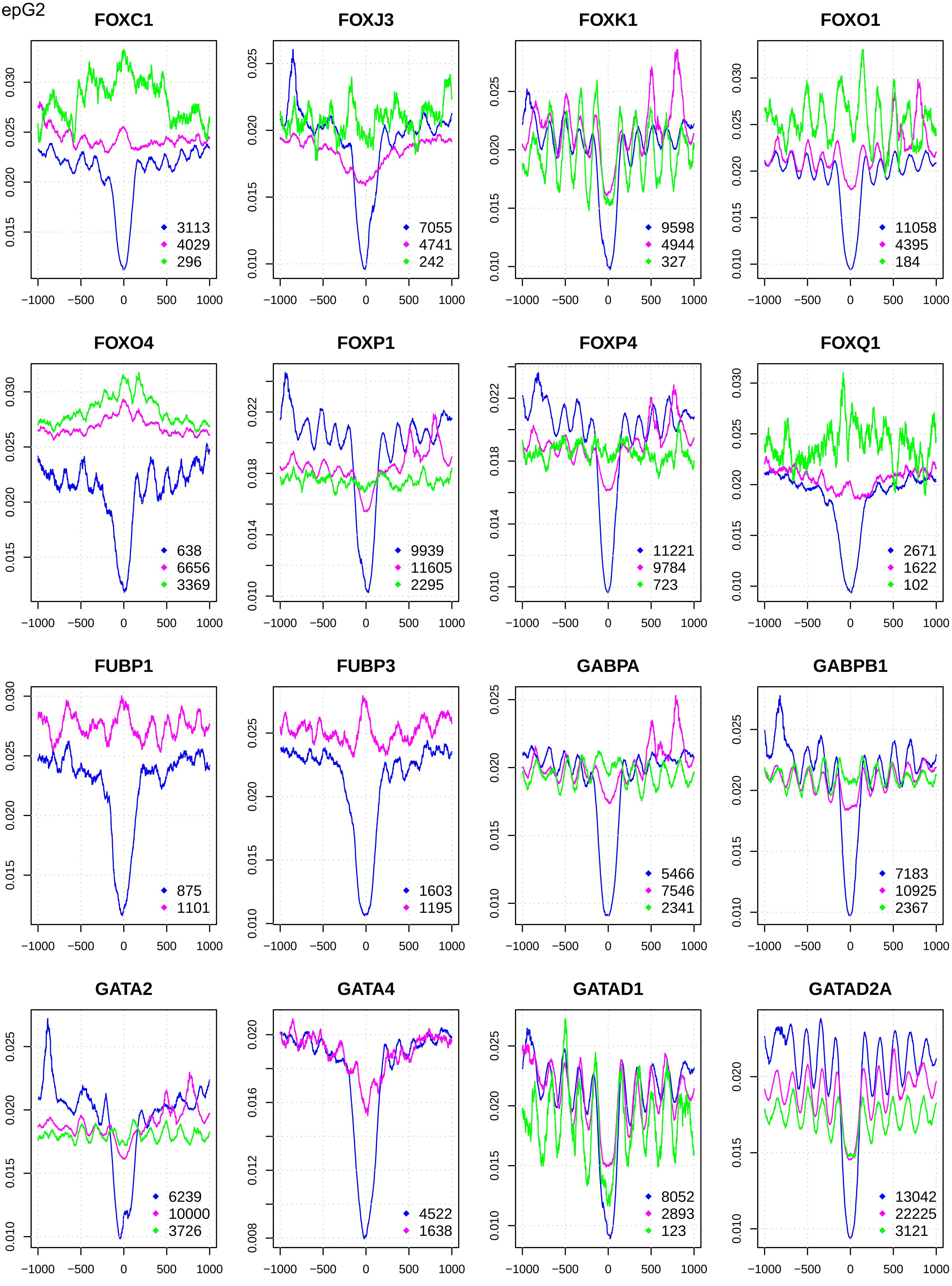
FOXC1
3113
4029
296
FOXJ3
7055
4741
242
FOXK1
9598
4944
327
FOXO1
11058
4395
184
FOXO4
638
6656
3369
FOXP1
9939
11605
2295
FOXP4
11221
9784
723
FOXQ1
2671
1622
102
FUBP1
875
1101
FUBP3
1603
1195
GABPA
5466
7546
2341
GABPB1
7183
10925
2367
GATA2
6239
10000
3726
GATA4
4522
1638
GATAD1
8052
2893
123
GATAD2A
13042
22225
3121

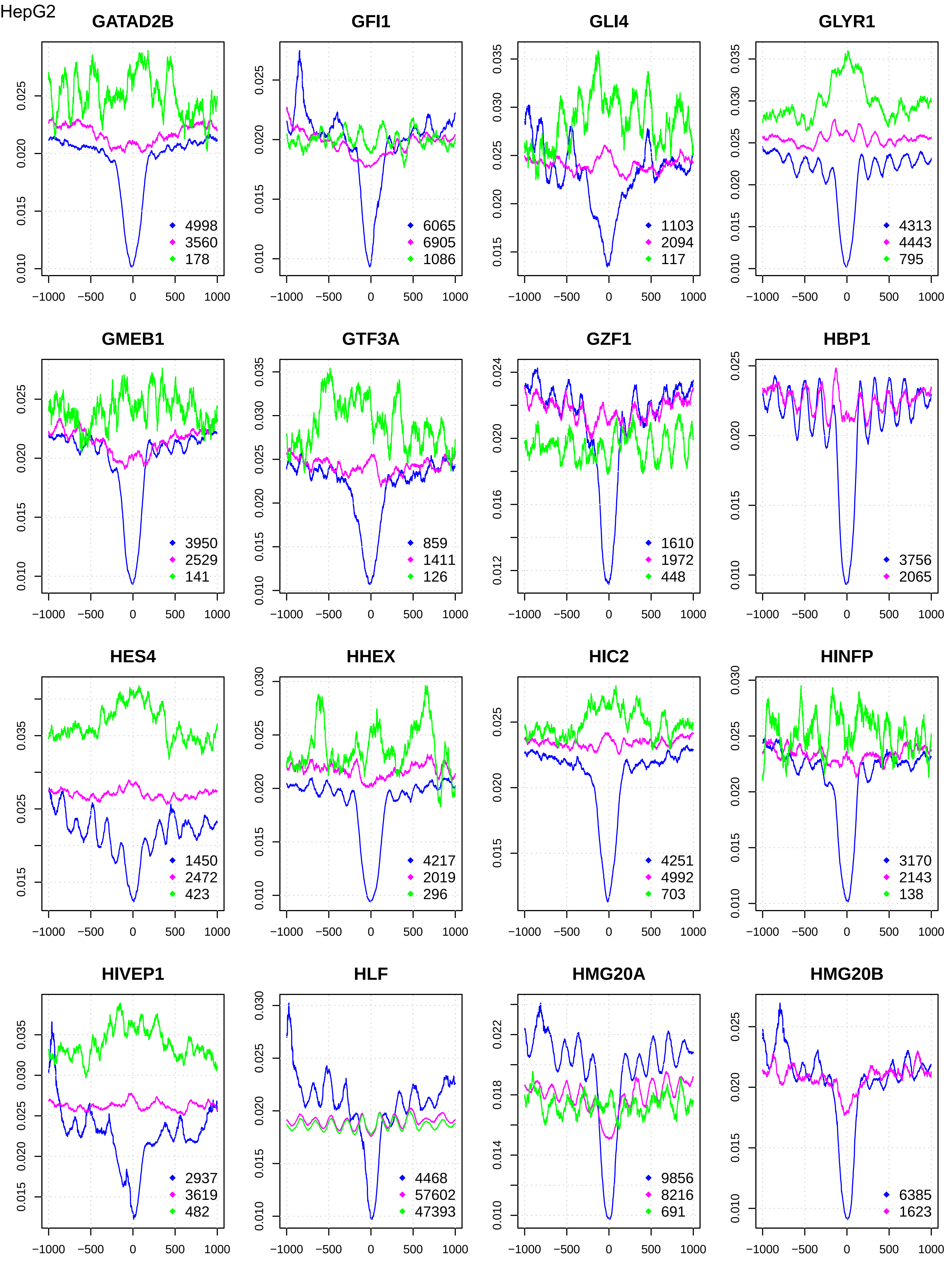
HepG2
GATAD2B
4998
3560
178
GFI1
6065
6905
1086
GLI4
1103
2094
117
GLYR1
4313
4443
795
GMEB1
3950
2529
141
GTF3A
859
1411
126
GZF1
1610
1972
448
HBP1
3756
2065
HES4
1450
2472
423
HHEX
4217
2019
296
HIC2
4251
4992
703
HINFP
3170
2143
138
HIVEP1
2937
3619
482
HLF
4468
57602
47393
HMG20A
9856
8216
691
HMG20B
6385
1623

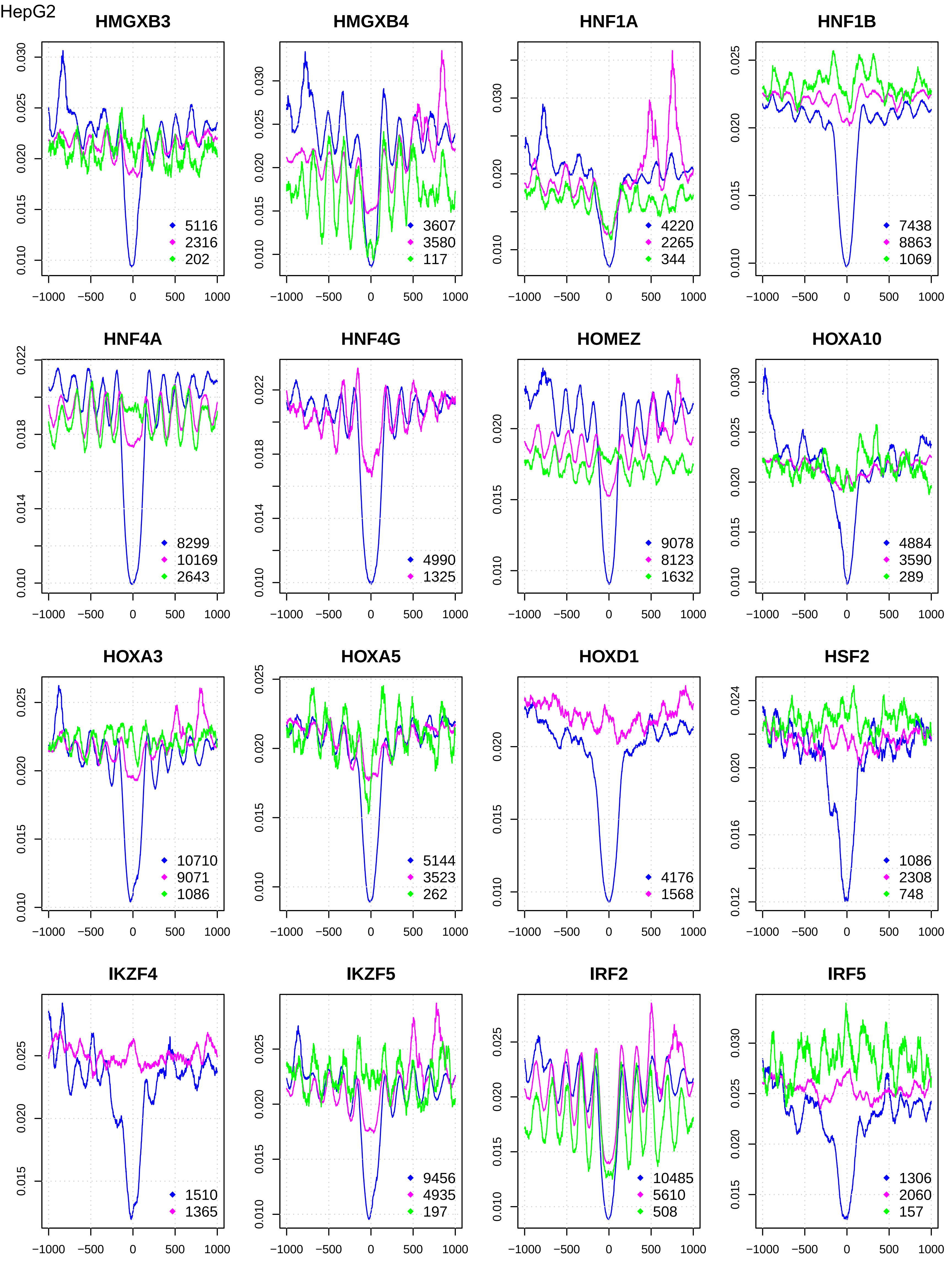
HepG2
HMGXB3
5116
2316
202
HMGXB4
3607
3580
117
HNF1A
4220
2265
344
HNF1B
7438
8863
1069
HNF4A
8299
10169
2643
HNF4G
4990
1325
HOMEZ
9078
8123
1632
HOXA10
4884
3590
289
HOXA3
10710
9071
1086
HOXA5
5144
3523
262
HOXD1
4176
1568
HSF2
1086
2308
748
IKZF4
1510
1365
IKZF5
9456
4935
197
IRF2
10485
5610
508
IRF5
1306
2060
157

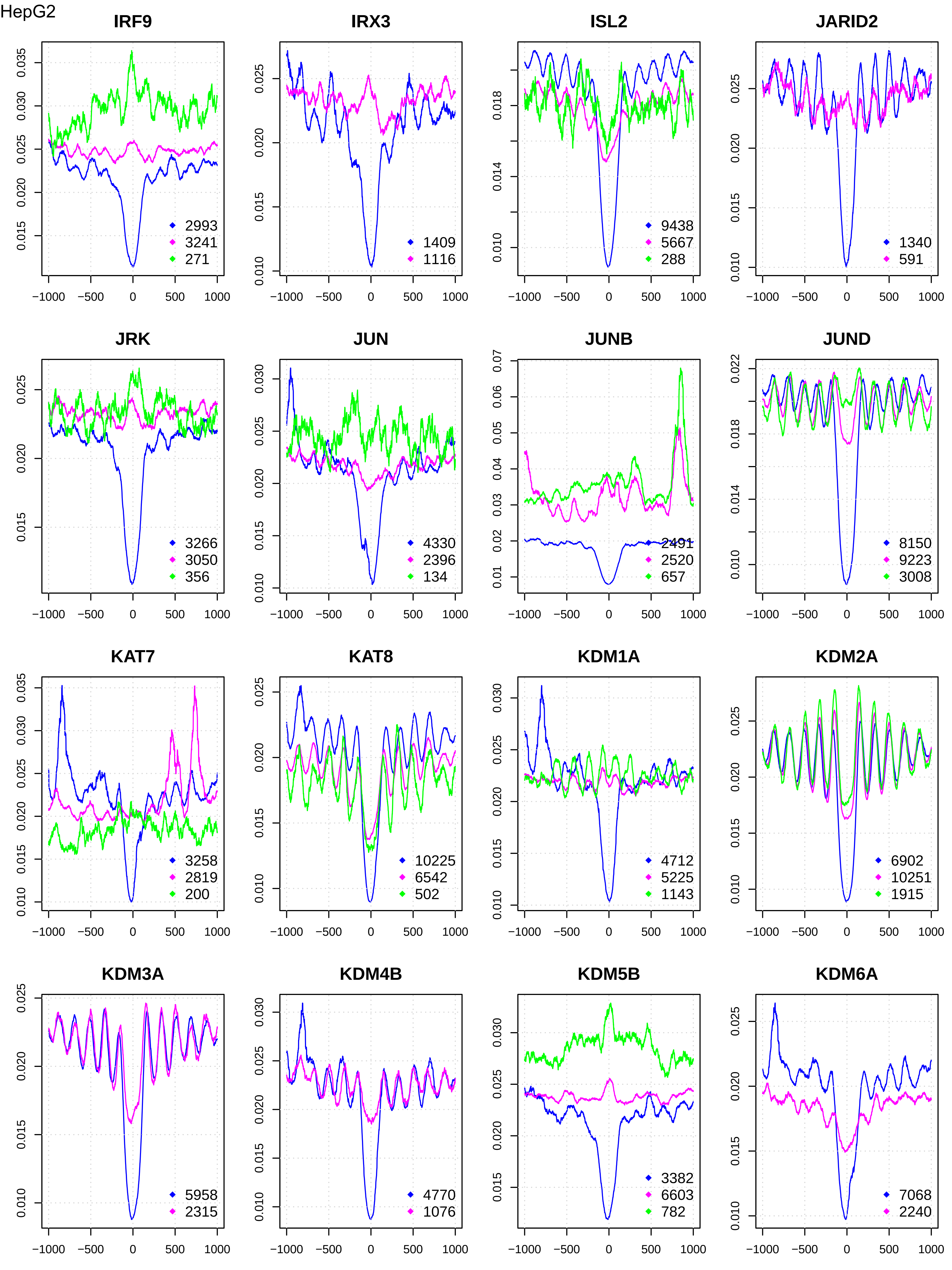
HepG2
IRF9
2993
3241
271
IRX3
1409
1116
ISL2
9438
5667
288
JARID2
1340
591
JRK
3266
3050
356
JUN
4330
2396
134
JUNB
2491
2520
657
JUND
8150
9223
3008
KAT7
3258
2819
200
KAT8
10225
6542
502
KDM1A
4712
5225
1143
KDM2A
6902
10251
1915
KDM3A
5958
2315
KDM4B
4770
1076
KDM5B
3382
6603
782
KDM6A
7068
2240
−1000 −500 0 500 1000

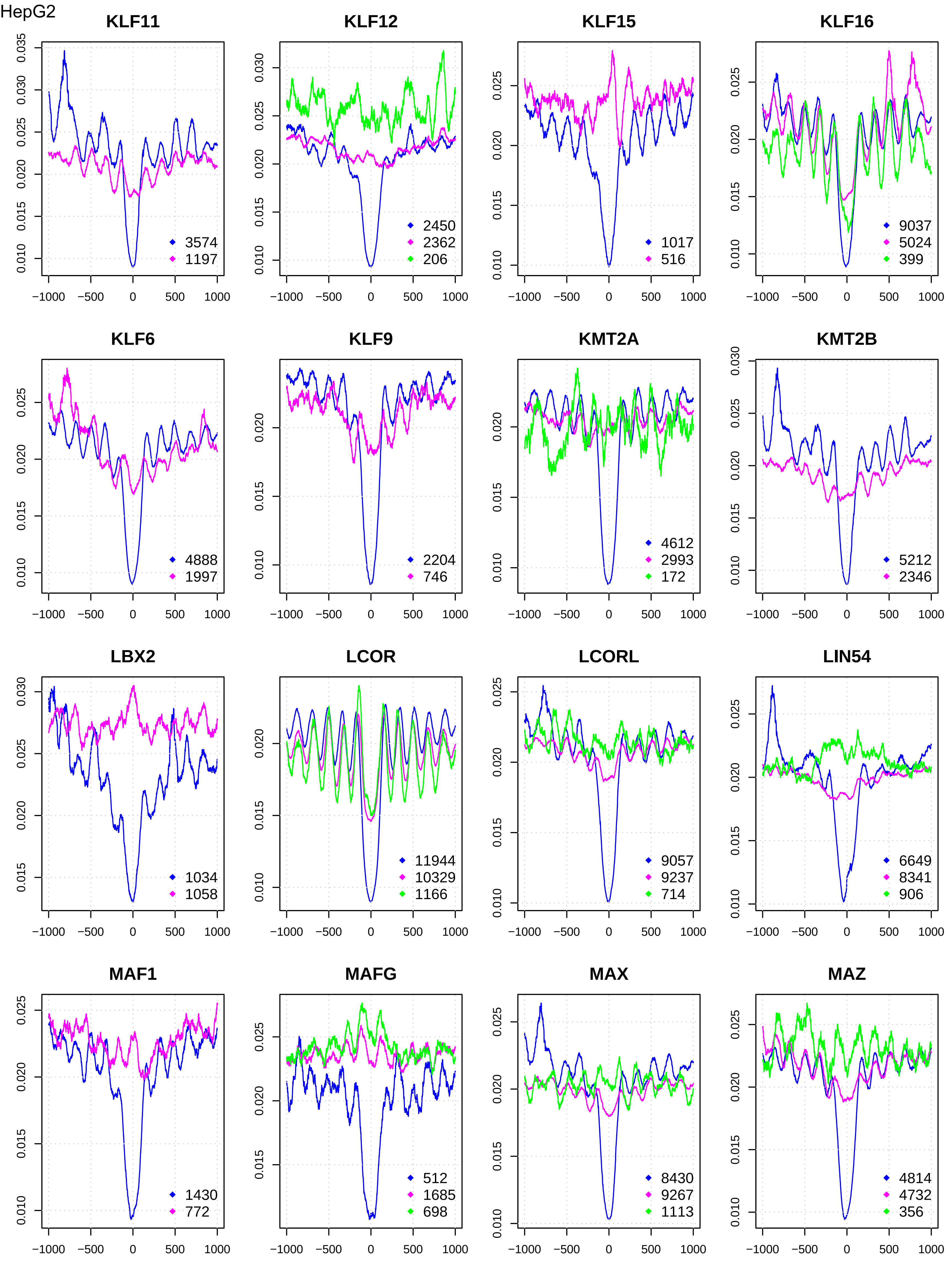
HepG2
KLF11
3574
1197
KLF12
2450
2362
206
KLF15
1017
516
KLF16
9037
5024
399
KLF6
4888
1997
KLF9
2204
746
KMT2A
4612
2993
172
KMT2B
5212
2346
LBX2
1034
1058
LCOR
11944
10329
1166
LCORL
9057
9237
714
LIN54
6649
8341
906
MAF1
1430
772
MAFG
512
1685
698
MAX
8430
9267
1113
MAZ
4814
4732
356
−1000 −500 0 500 1000
0.010 0.015 0.020 0.025 0.030 0.035

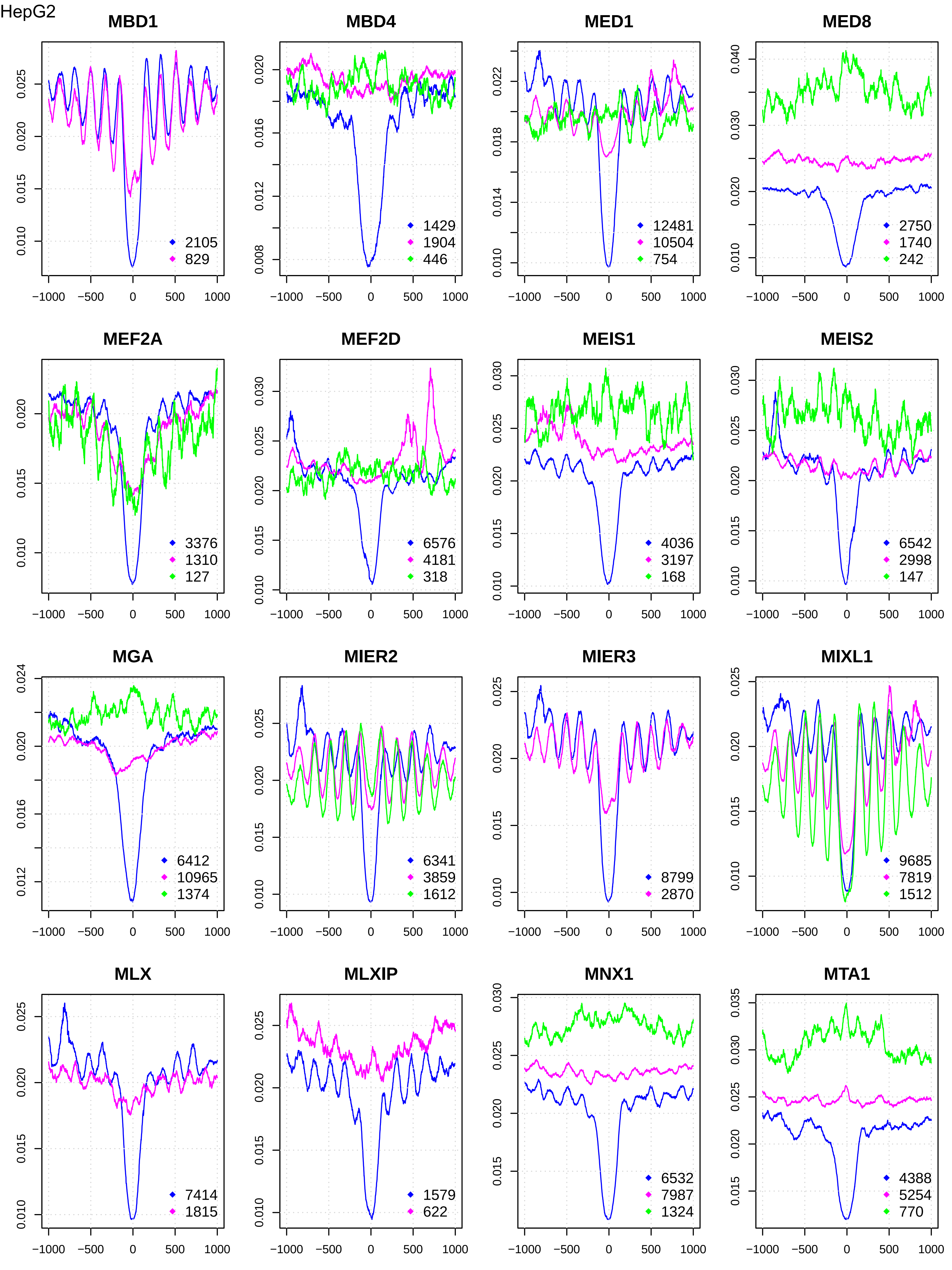
HepG2
MBD1
2105
829
MBD4
1429
1904
446
MED1
12481
10504
754
MED8
2750
1740
242
MEF2A
3376
1310
127
MEF2D
6576
4181
318
MEIS1
4036
3197
168
MEIS2
6542
2998
147
MGA
6412
10965
1374
MIER2
6341
3859
1612
MIER3
8799
2870
MIXL1
9685
7819
1512
MLX
7414
1815
MLXIP
1579
622
MNX1
6532
7987
1324
MTA1
4388
5254
770

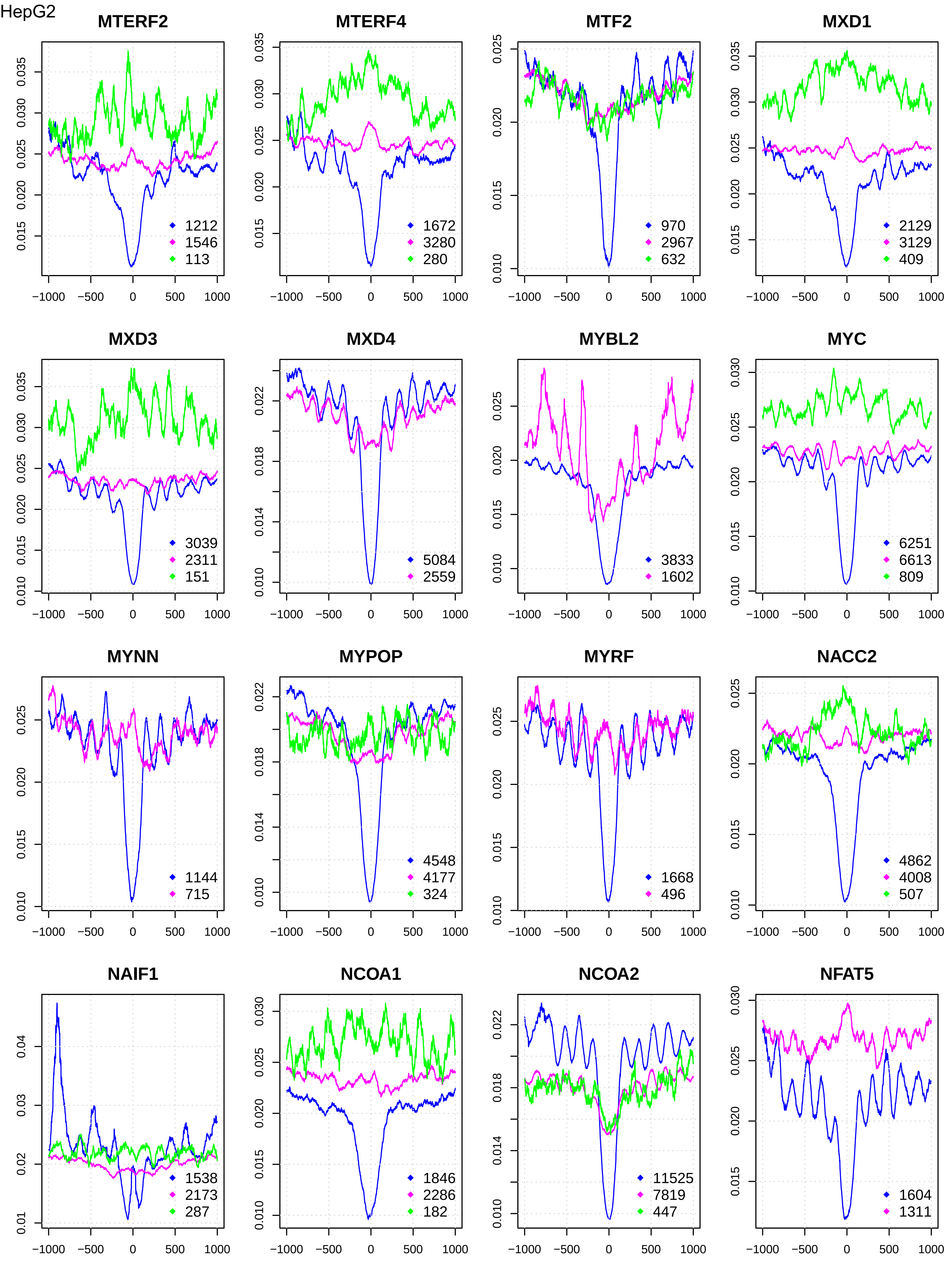

HepG2
MTERF2
1212
1546
113
MTERF4
1672
3280
280
MTF2
970
2967
632
MXD1
2129
3129
409
MXD3
3039
2311
151
MXD4
5084
2559
MYBL2
3833
1602
MYC
6251
6613
809
MYNN
1144
715
MYPOP
4548
4177
324
MYRF
1668
496
NACC2
4862
4008
507
NAIF1
1538
2173
287
NCOA1
1846
2286
182
NCOA2
11525
7819
447
NFAT5
1604
1311
−1000
−500
0
500
1000

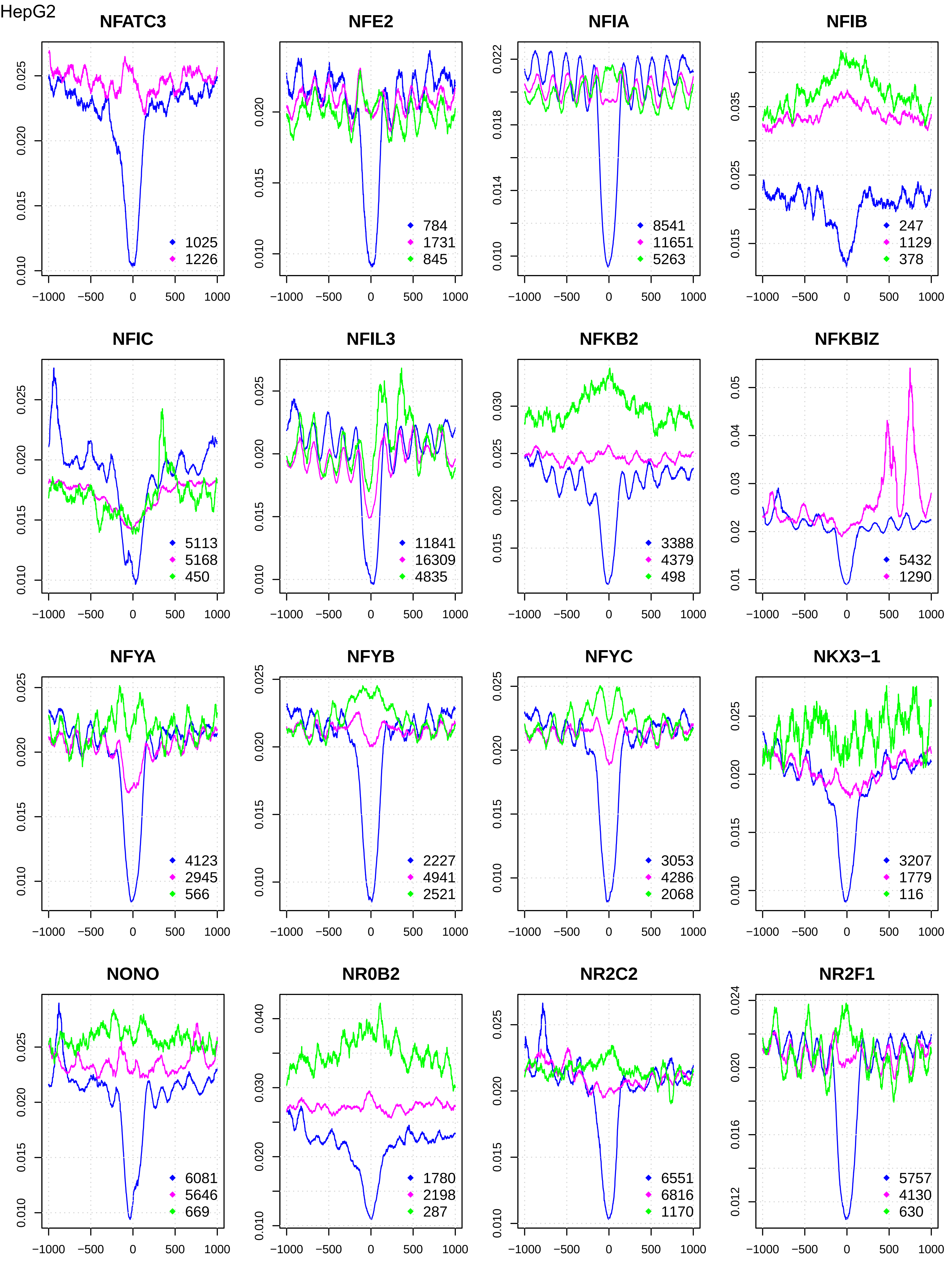
HepG2
NFATC3
1025
1226
NFE2
784
1731
845
NFIA
8541
11651
5263
NFIB
247
1129
378
NFIC
5113
5168
450
NFIL3
11841
16309
4835
NFKB2
3388
4379
498
NFKBIZ
5432
1290
NFYA
4123
2945
566
NFYB
2227
4941
2521
NFYC
3053
4286
2068
NKX3−1
3207
1779
116
NONO
6081
5646
669
NR0B2
1780
2198
287
NR2C2
6551
6816
1170
NR2F1
5757
4130
630

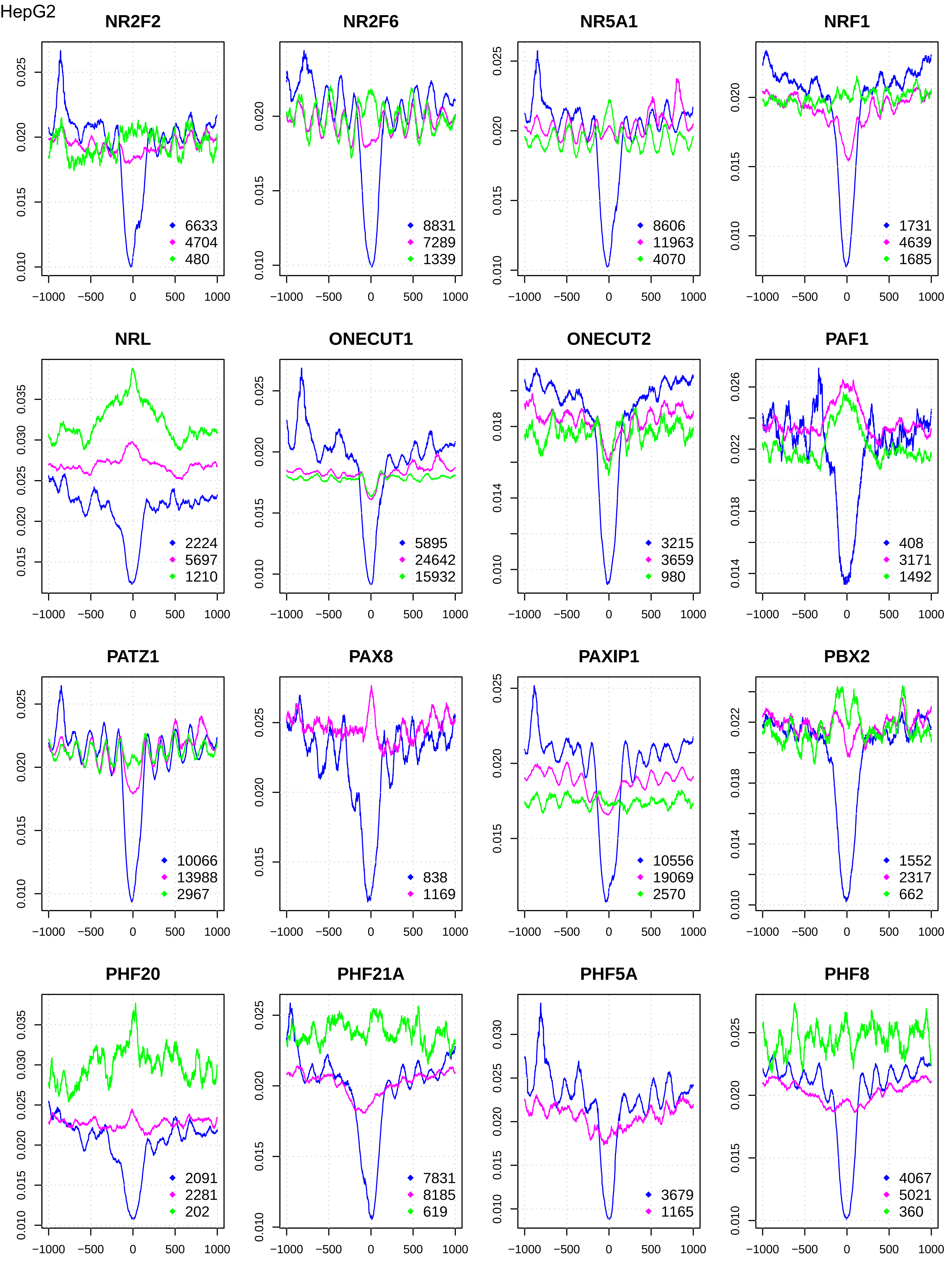
HepG2
NR2F2
6633
4704
480
NR2F6
8831
7289
1339
NR5A1
8606
11963
4070
NRF1
1731
4639
1685
NRL
2224
5697
1210
ONECUT1
5895
24642
15932
ONECUT2
3215
3659
980
PAF1
408
3171
1492
PATZ1
10066
13988
2967
PAX8
838
1169
PAXIP1
10556
19069
2570
PBX2
1552
2317
662
PHF20
2091
2281
202
PHF21A
7831
8185
619
PHF5A
3679
1165
PHF8
4067
5021
360

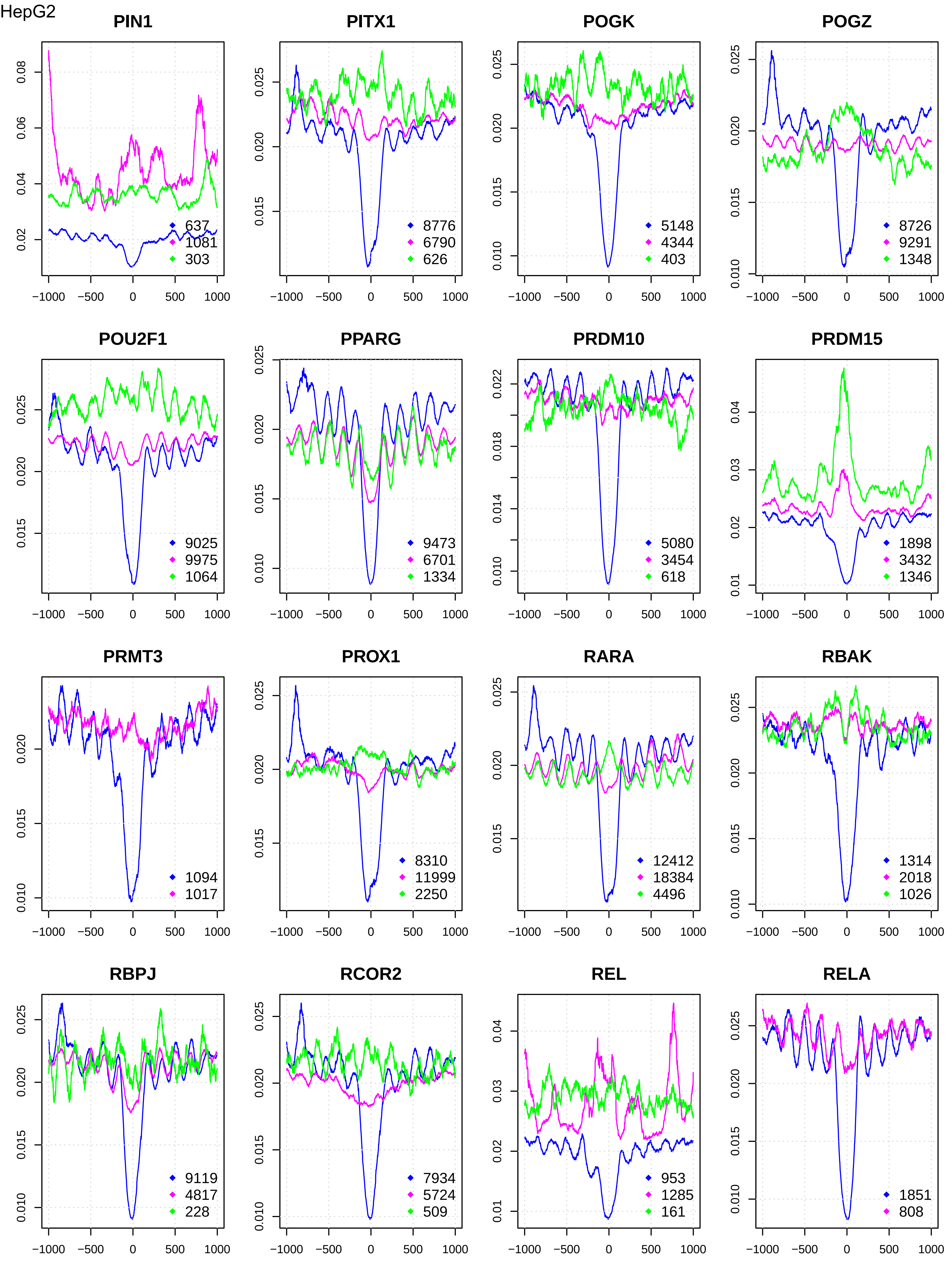
HepG2
PIN1
637
1081
303
PITX1
8776
6790
626
POGK
5148
4344
403
POGZ
8726
9291
1348
POU2F1
9025
9975
1064
PPARG
9473
6701
1334
PRDM10
5080
3454
618
PRDM15
1898
3432
1346
PRMT3
1094
1017
PROX1
8310
11999
2250
RARA
12412
18384
4496
RBAK
1314
2018
1026
RBPJ
9119
4817
228
RCOR2
7934
5724
509
REL
953
1285
161
RELA
1851
808
−1000
−500
0
500
1000

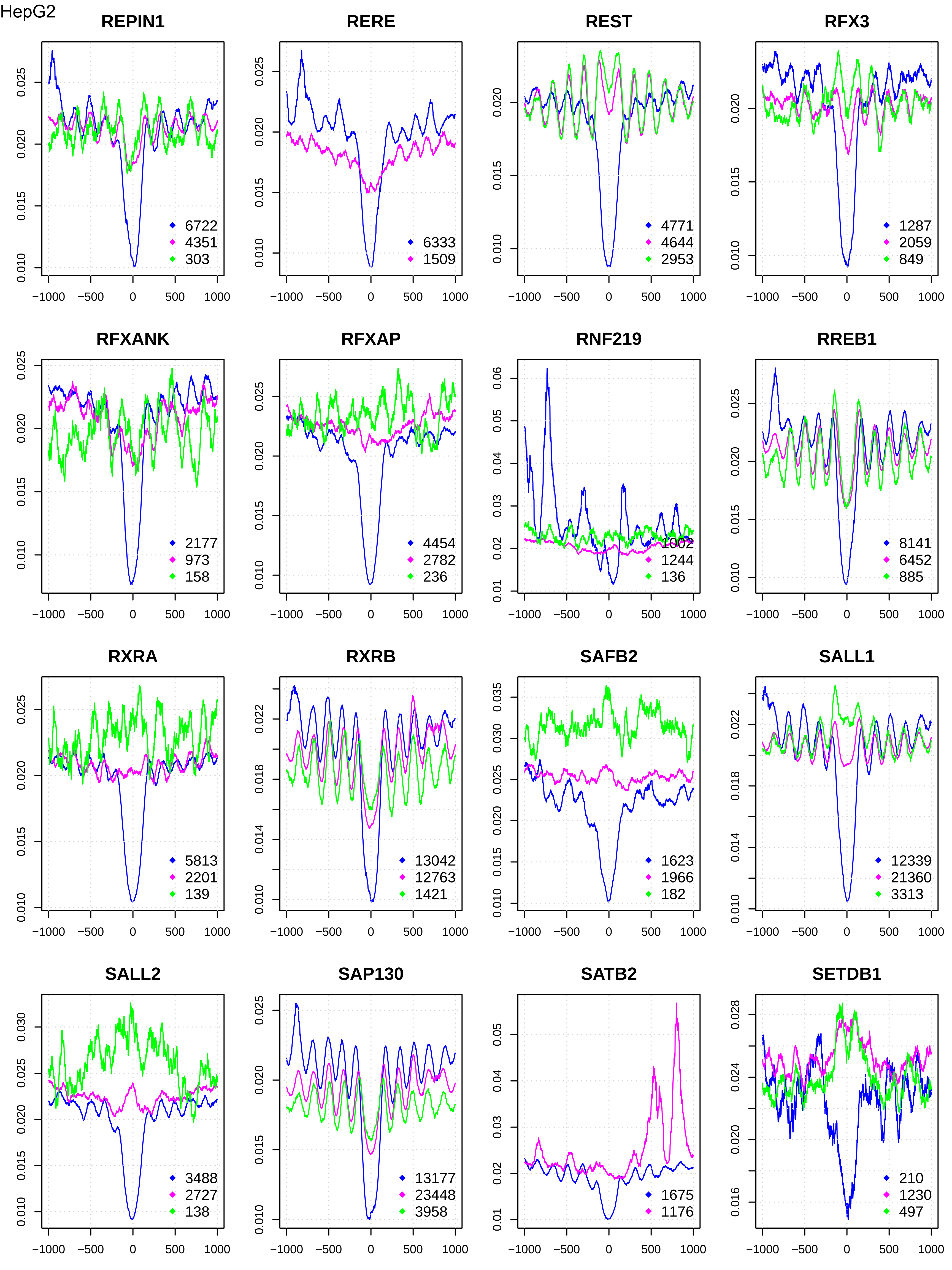
HepG2
REPIN1
6722
4351
303
RERE
6333
1509
REST
4771
4644
2953
RFX3
1287
2059
849
RFXANK
2177
973
158
RFXAP
4454
2782
236
RNF219
1002
1244
136
RREB1
8141
6452
885
RXRA
5813
2201
139
RXRB
13042
12763
1421
SAFB2
1623
1966
182
SALL1
12339
21360
3313
SALL2
3488
2727
138
SAP130
13177
23448
3958
SATB2
1675
1176
SETDB1
210
1230
497

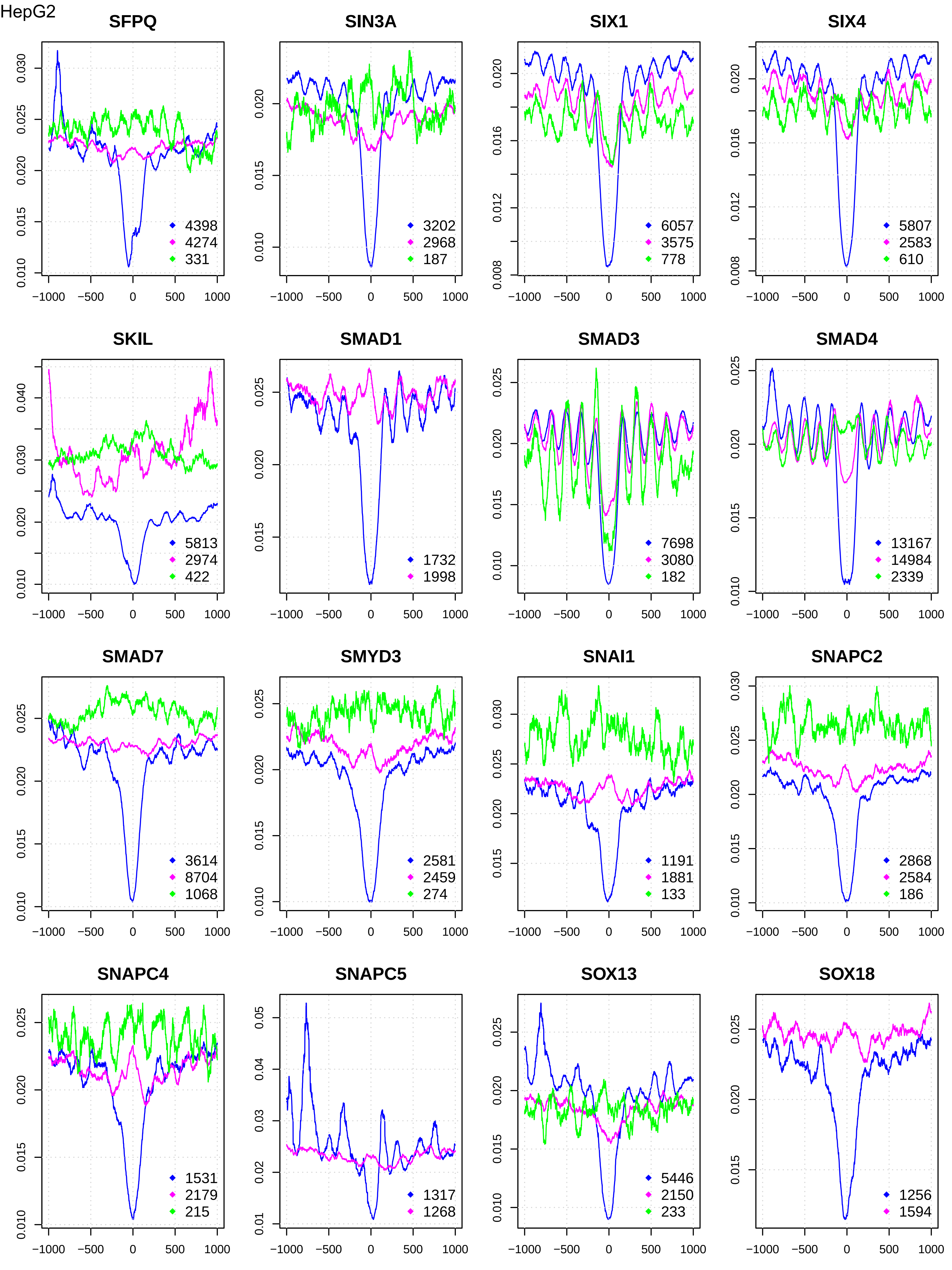
HepG2
SFPQ
4398
4274
331
SIN3A
3202
2968
187
SIX1
6057
3575
778
SIX4
5807
2583
610
SKIL
5813
2974
422
SMAD1
1732
1998
SMAD3
7698
3080
182
SMAD4
13167
14984
2339
SMAD7
3614
8704
1068
SMYD3
2581
2459
274
SNAI1
1191
1881
133
SNAPC2
2868
2584
186
SNAPC4
1531
2179
215
SNAPC5
1317
1268
SOX13
5446
2150
233
SOX18
1256
1594

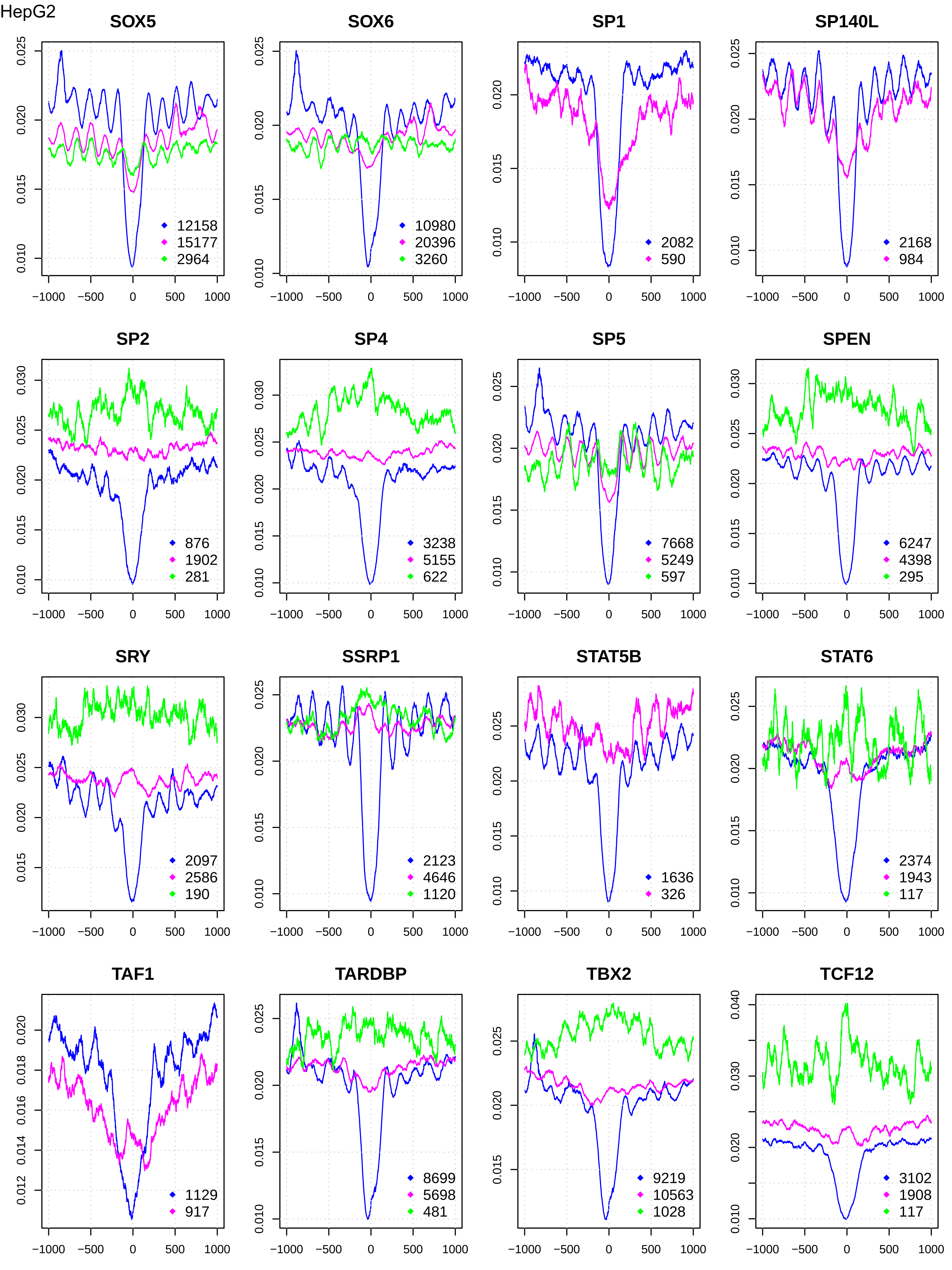
HepG2
SOX5
12158
15177
2964
SOX6
10980
20396
3260
SP1
2082
590
SP140L
2168
984
SP2
876
1902
281
SP4
3238
5155
622
SP5
7668
5249
597
SPEN
6247
4398
295
SRY
2097
2586
190
SSRP1
2123
4646
1120
STAT5B
1636
326
STAT6
2374
1943
117
TAF1
1129
917
TARDBP
8699
5698
481
TBX2
9219
10563
1028
TCF12
3102
1908
117

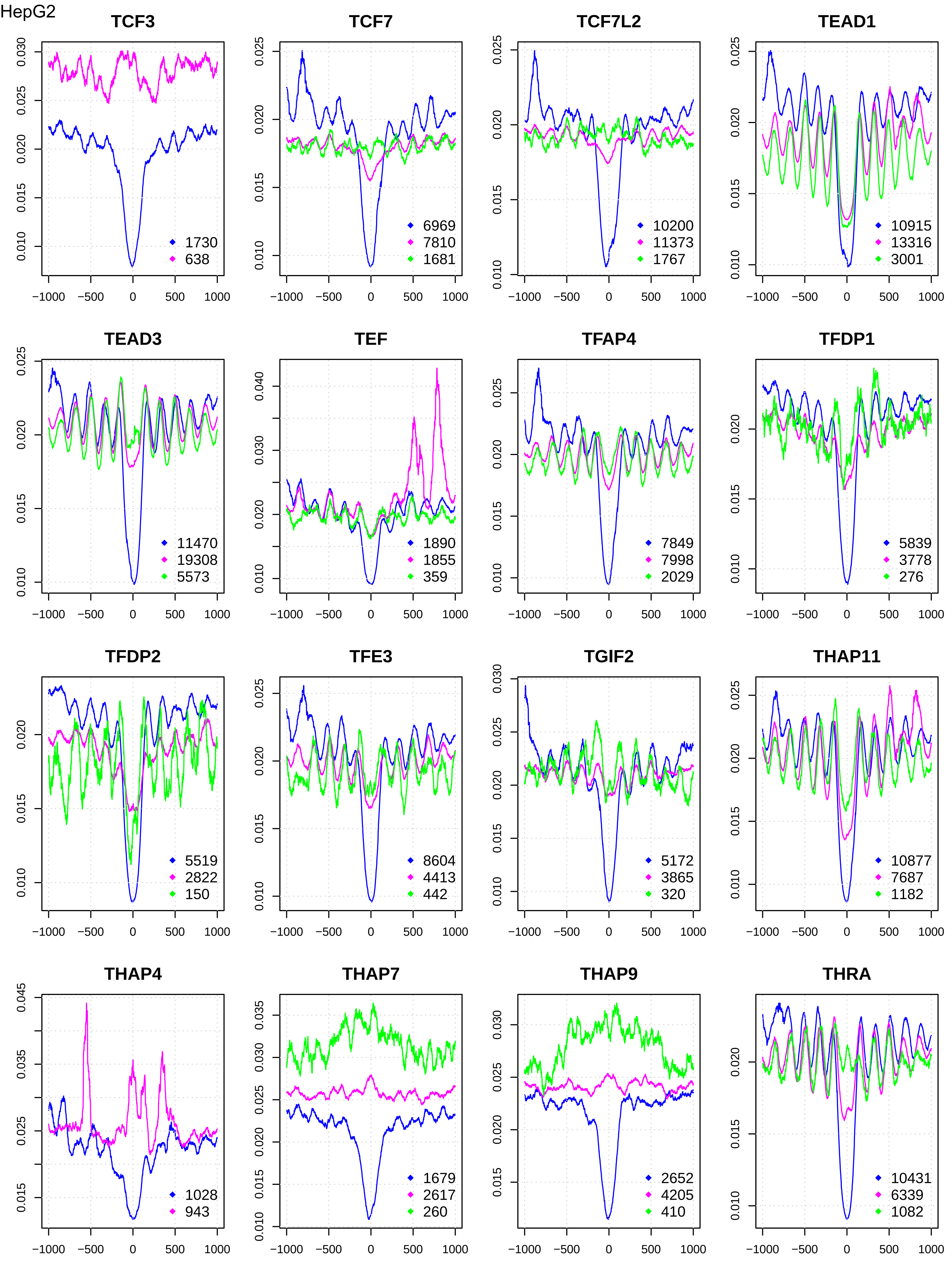
HepG2
TCF3
1730
638
TCF7
6969
7810
1681
TCF7L2
10200
11373
1767
TEAD1
10915
13316
3001
TEAD3
11470
19308
5573
TEF
1890
1855
359
TFAP4
7849
7998
2029
TFDP1
5839
3778
276
TFDP2
5519
2822
150
TFE3
8604
4413
442
TGIF2
5172
3865
320
THAP11
10877
7687
1182
THAP4
1028
943
THAP7
1679
2617
260
THAP9
2652
4205
410
THRA
10431
6339
1082
−1000
−500
0
500
1000

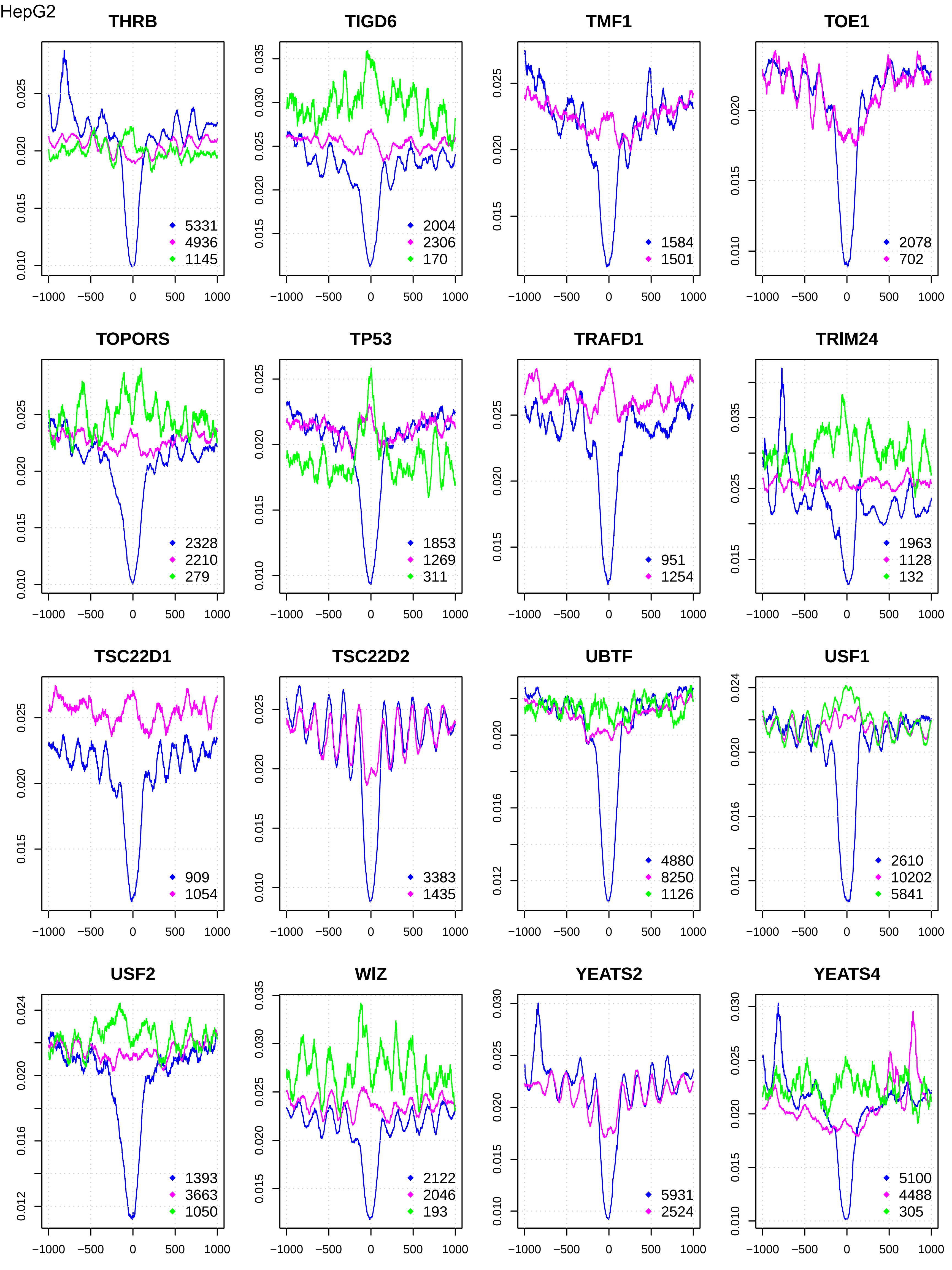
HepG2
THRB
5331
4936
1145
TIGD6
2004
2306
170
TMF1
1584
1501
TOE1
2078
702
TOPORS
2328
2210
279
TP53
1853
1269
311
TRAFD1
951
1254
TRIM24
1963
1128
132
TSC22D1
909
1054
TSC22D2
3383
1435
UBTF
4880
8250
1126
USF1
2610
10202
5841
USF2
1393
3663
1050
WIZ
2122
2046
193
YEATS2
5931
2524
YEATS4
5100
4488
305

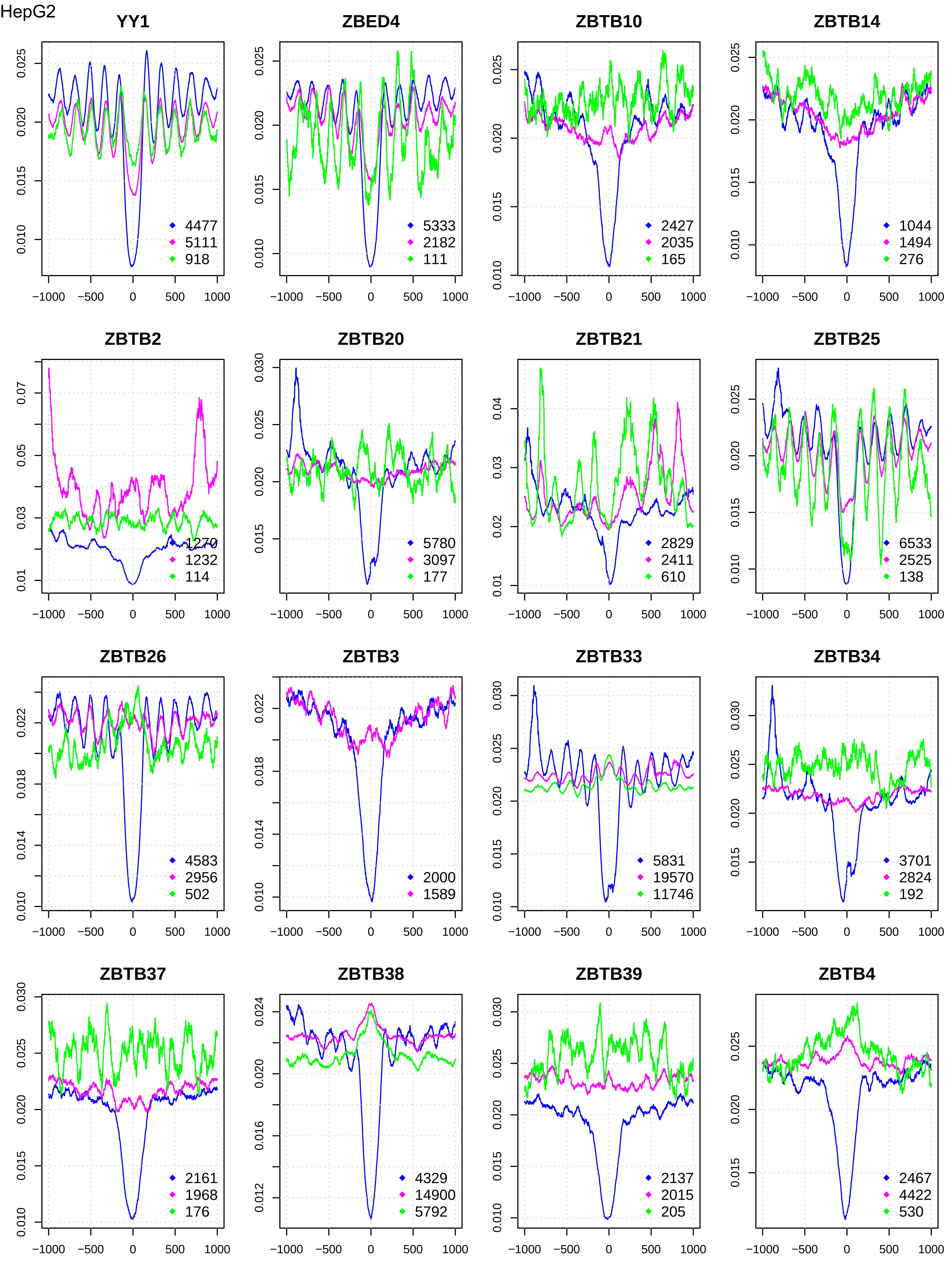
HepG2
YY1
4477
5111
918
ZBED4
5333
2182
111
ZBTB10
2427
2035
165
ZBTB14
1044
1494
276
ZBTB2
1270
1232
114
ZBTB20
5780
3097
177
ZBTB21
2829
2411
610
ZBTB25
6533
2525
138
ZBTB26
4583
2956
502
ZBTB3
2000
1589
ZBTB33
5831
19570
11746
ZBTB34
3701
2824
192
ZBTB37
2161
1968
176
ZBTB38
4329
14900
5792
ZBTB39
2137
2015
205
ZBTB4
2467
4422
530
−1000 −500 0 500 1000

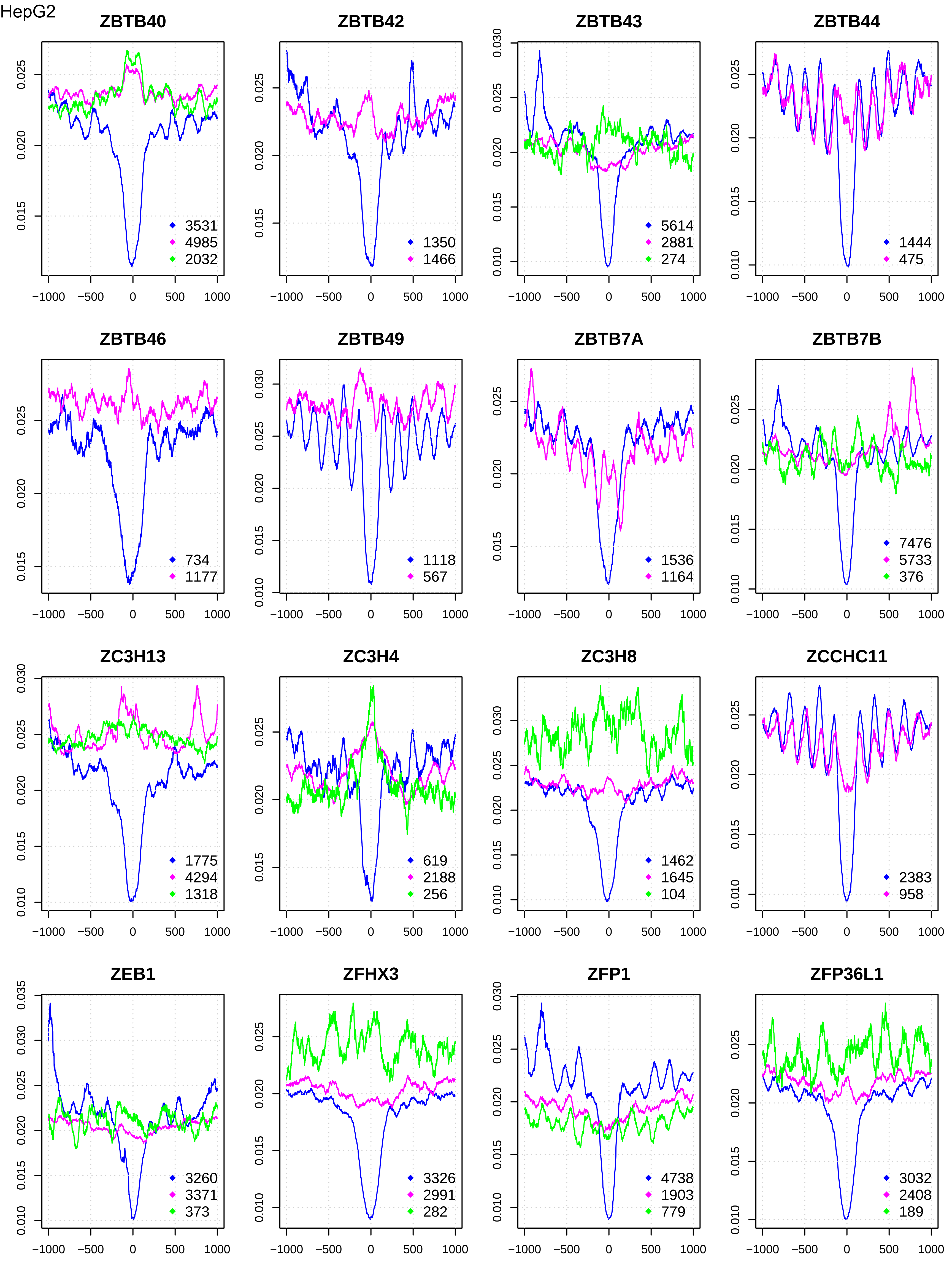
HepG2
ZBTB40
3531
4985
2032
ZBTB42
1350
1466
ZBTB43
5614
2881
274
ZBTB44
1444
475
ZBTB46
734
1177
ZBTB49
1118
567
ZBTB7A
1536
1164
ZBTB7B
7476
5733
376
ZC3H13
1775
4294
1318
ZC3H4
619
2188
256
ZC3H8
1462
1645
104
ZCCHC11
2383
958
ZEB1
3260
3371
373
ZFHX3
3326
2991
282
ZFP1
4738
1903
779
ZFP36L1
3032
2408
189
−1000
−500
0
500
1000

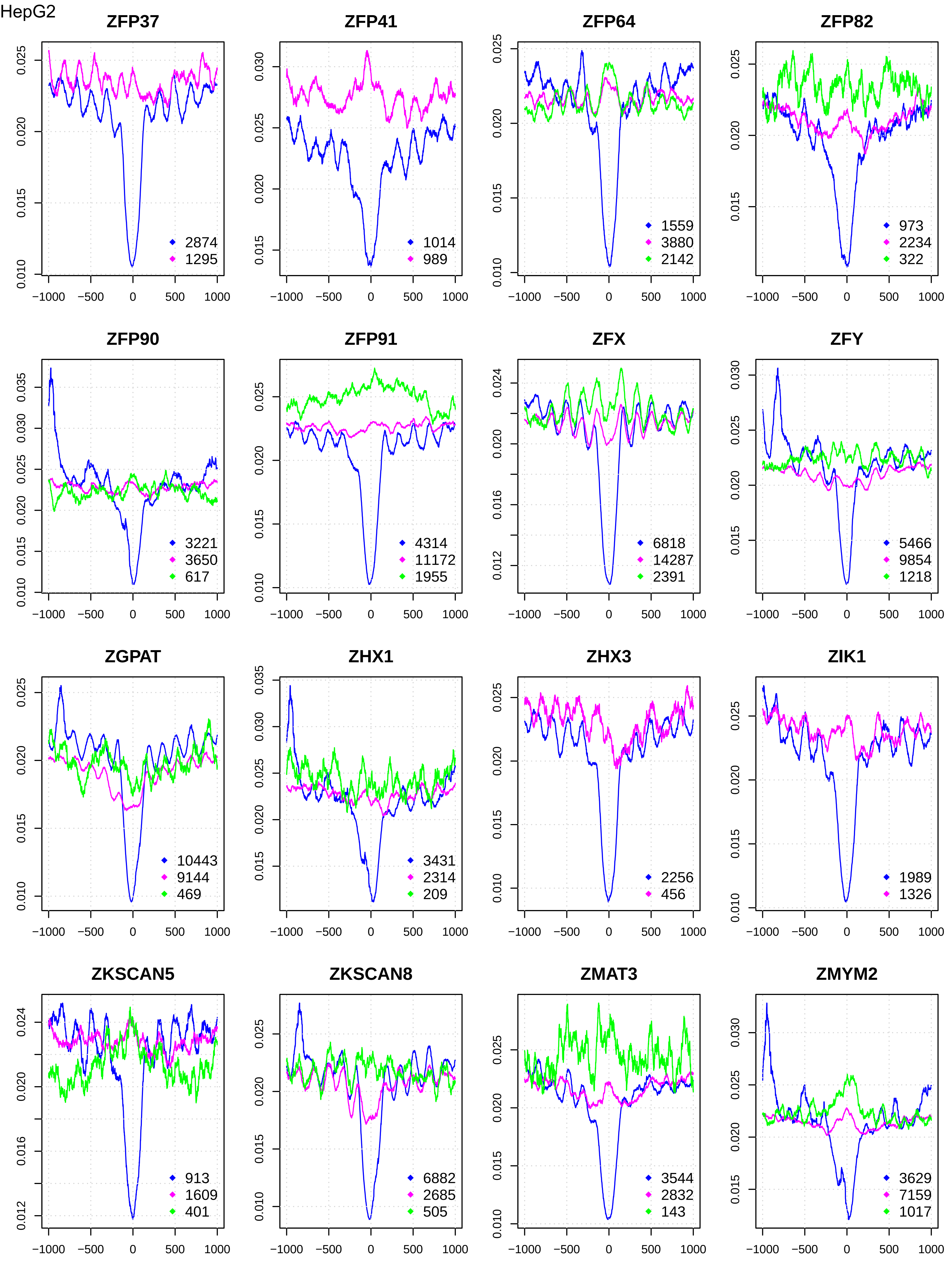
HepG2
ZFP37
2874
1295
ZFP41
1014
989
ZFP64
1559
3880
2142
ZFP82
973
2234
322
ZFP90
3221
3650
617
ZFP91
4314
11172
1955
ZFX
6818
14287
2391
ZFY
5466
9854
1218
ZGPAT
10443
9144
469
ZHX1
3431
2314
209
ZHX3
2256
456
ZIK1
1989
1326
ZKSCAN5
913
1609
401
ZKSCAN8
6882
2685
505
ZMAT3
3544
2832
143
ZMYM2
3629
7159
1017

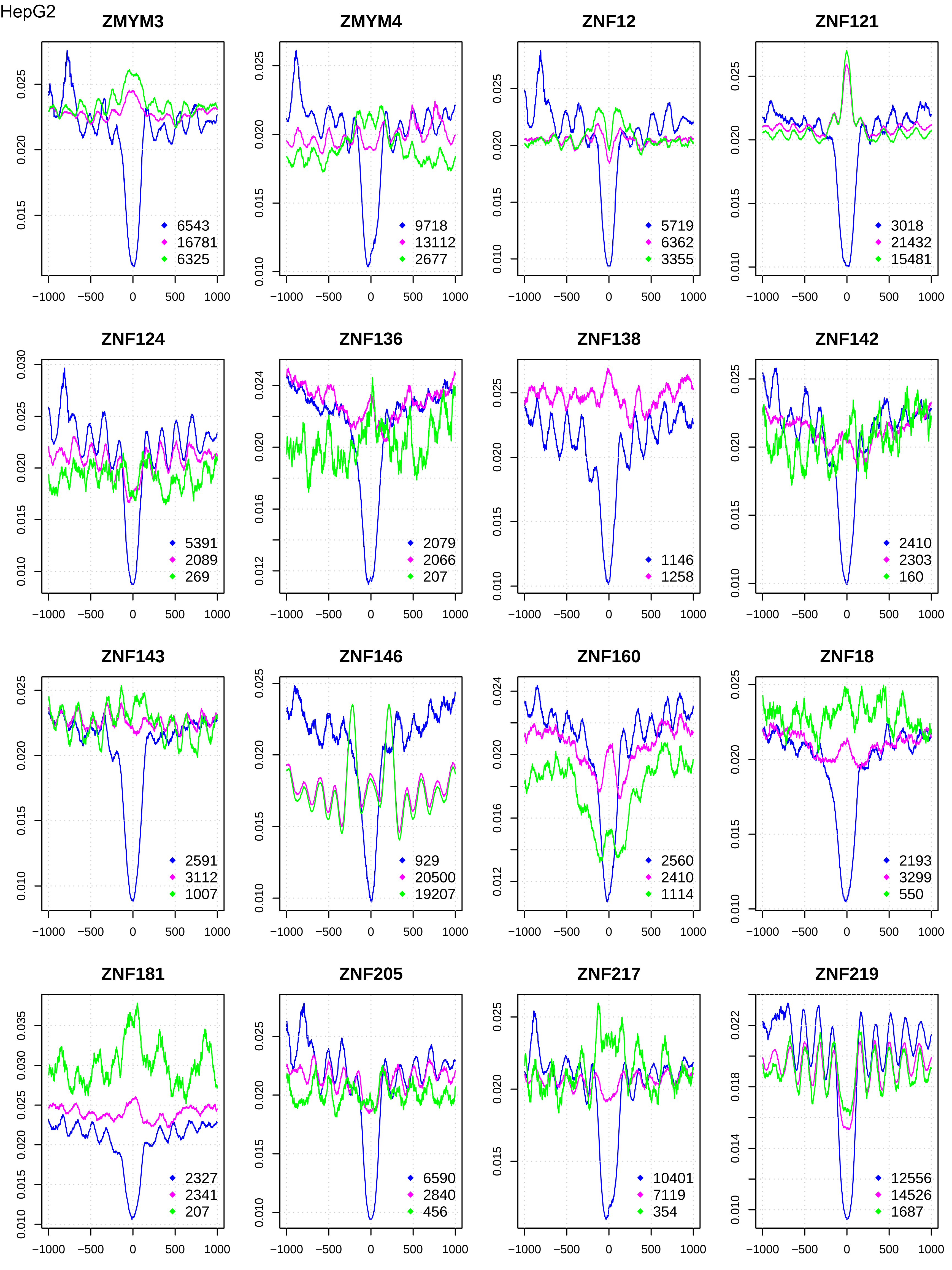
HepG2
ZMYM3
6543
16781
6325
ZMYM4
9718
13112
2677
ZNF12
5719
6362
3355
ZNF121
3018
21432
15481
ZNF124
5391
2089
269
ZNF136
2079
2066
207
ZNF138
1146
1258
ZNF142
2410
2303
160
ZNF143
2591
3112
1007
ZNF146
929
20500
19207
ZNF160
2560
2410
1114
ZNF18
2193
3299
550
ZNF181
2327
2341
207
ZNF205
6590
2840
456
ZNF217
10401
7119
354
ZNF219
12556
14526
1687

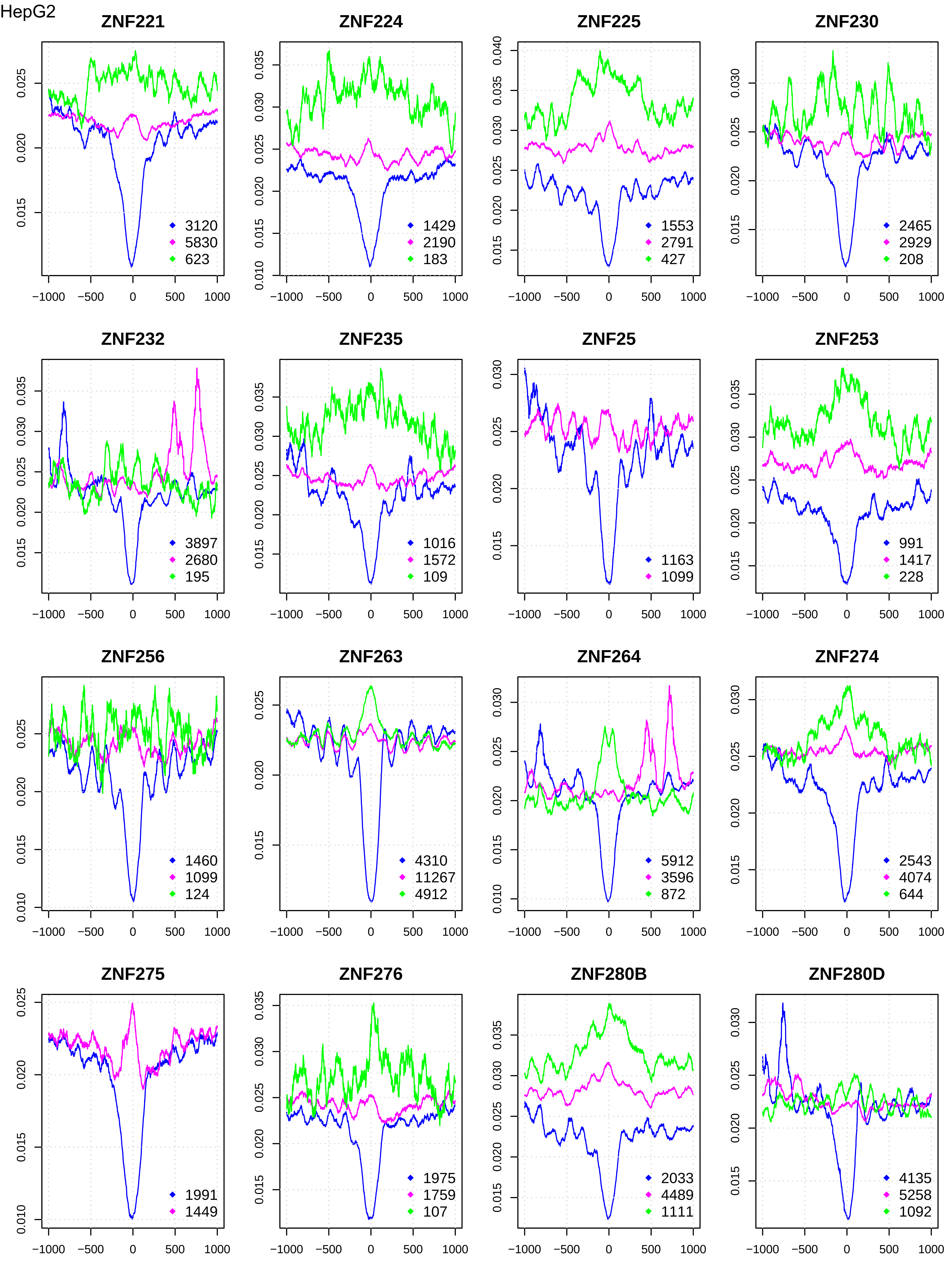
HepG2
ZNF221
3120
5830
623
ZNF224
1429
2190
183
ZNF225
1553
2791
427
ZNF230
2465
2929
208
ZNF232
3897
2680
195
ZNF235
1016
1572
109
ZNF25
1163
1099
ZNF253
991
1417
228
ZNF256
1460
1099
124
ZNF263
4310
11267
4912
ZNF264
5912
3596
872
ZNF274
2543
4074
644
ZNF275
1991
1449
ZNF276
1975
1759
107
ZNF280B
2033
4489
1111
ZNF280D
4135
5258
1092
−1000
−500
0
500
1000

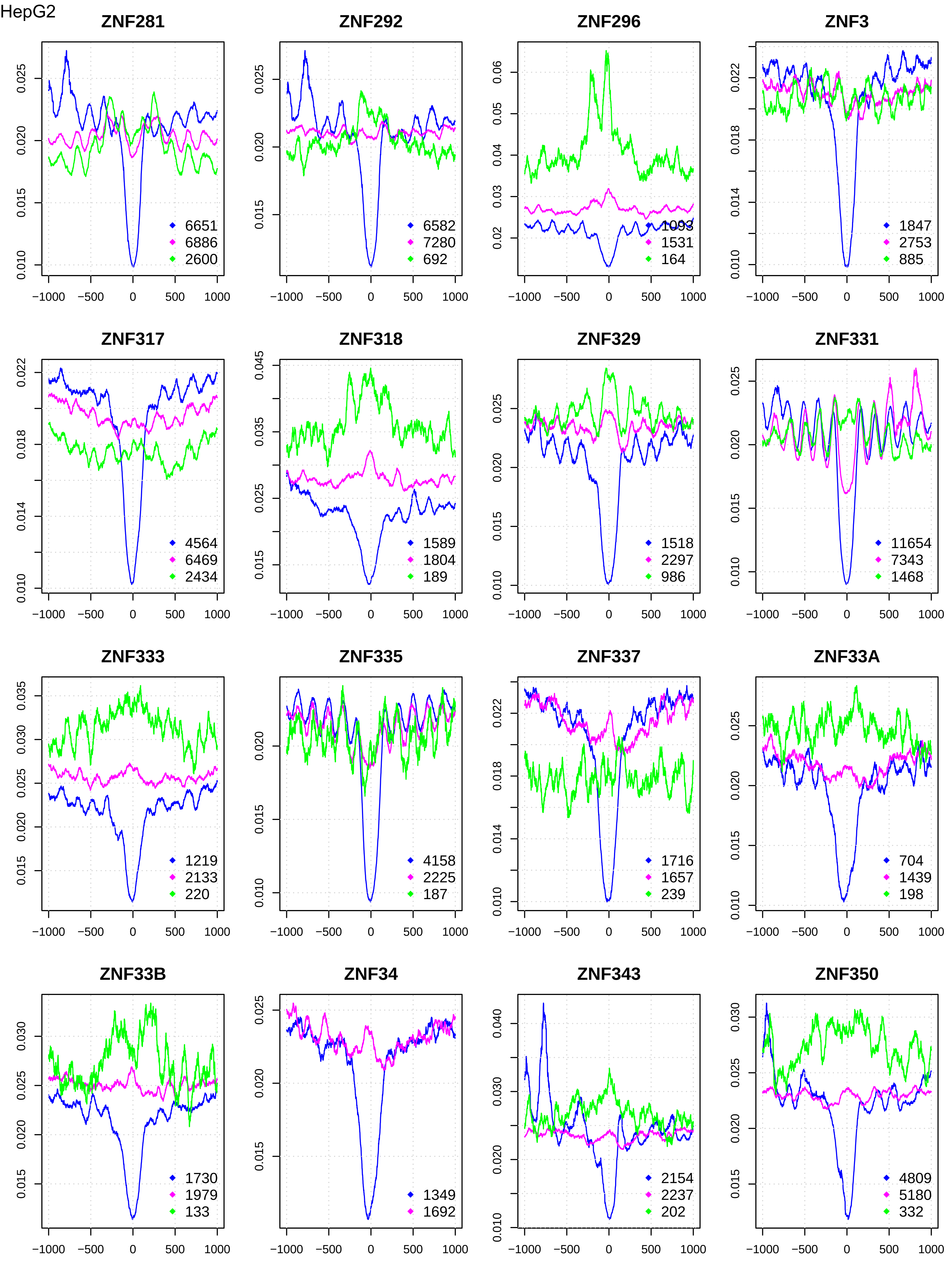
HepG2
ZNF281
6651
6886
2600
ZNF292
6582
7280
692
ZNF296
1093
1531
164
ZNF3
1847
2753
885
ZNF317
4564
6469
2434
ZNF318
1589
1804
189
ZNF329
1518
2297
986
ZNF331
11654
7343
1468
ZNF333
1219
2133
220
ZNF335
4158
2225
187
ZNF337
1716
1657
239
ZNF33A
704
1439
198
ZNF33B
1730
1979
133
ZNF34
1349
1692
ZNF343
2154
2237
202
ZNF350
4809
5180
332

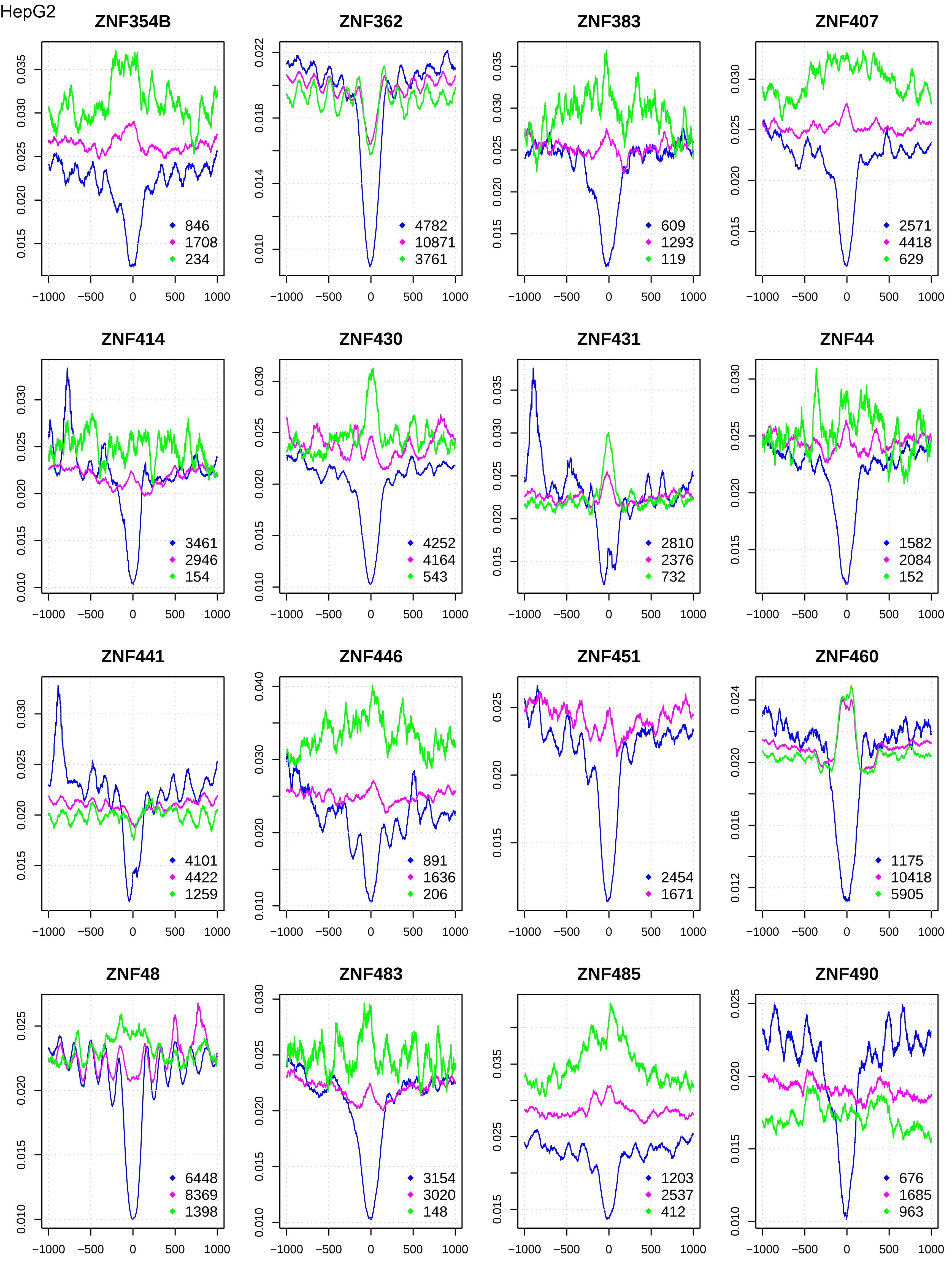
HepG2
ZNF354B
846
1708
234
ZNF362
4782
10871
3761
ZNF383
609
1293
119
ZNF407
2571
4418
629
ZNF414
3461
2946
154
ZNF430
4252
4164
543
ZNF431
2810
2376
732
ZNF44
1582
2084
152
ZNF441
4101
4422
1259
ZNF446
891
1636
206
ZNF451
2454
1671
ZNF460
1175
10418
5905
ZNF48
6448
8369
1398
ZNF483
3154
3020
148
ZNF485
1203
2537
412
ZNF490
676
1685
963

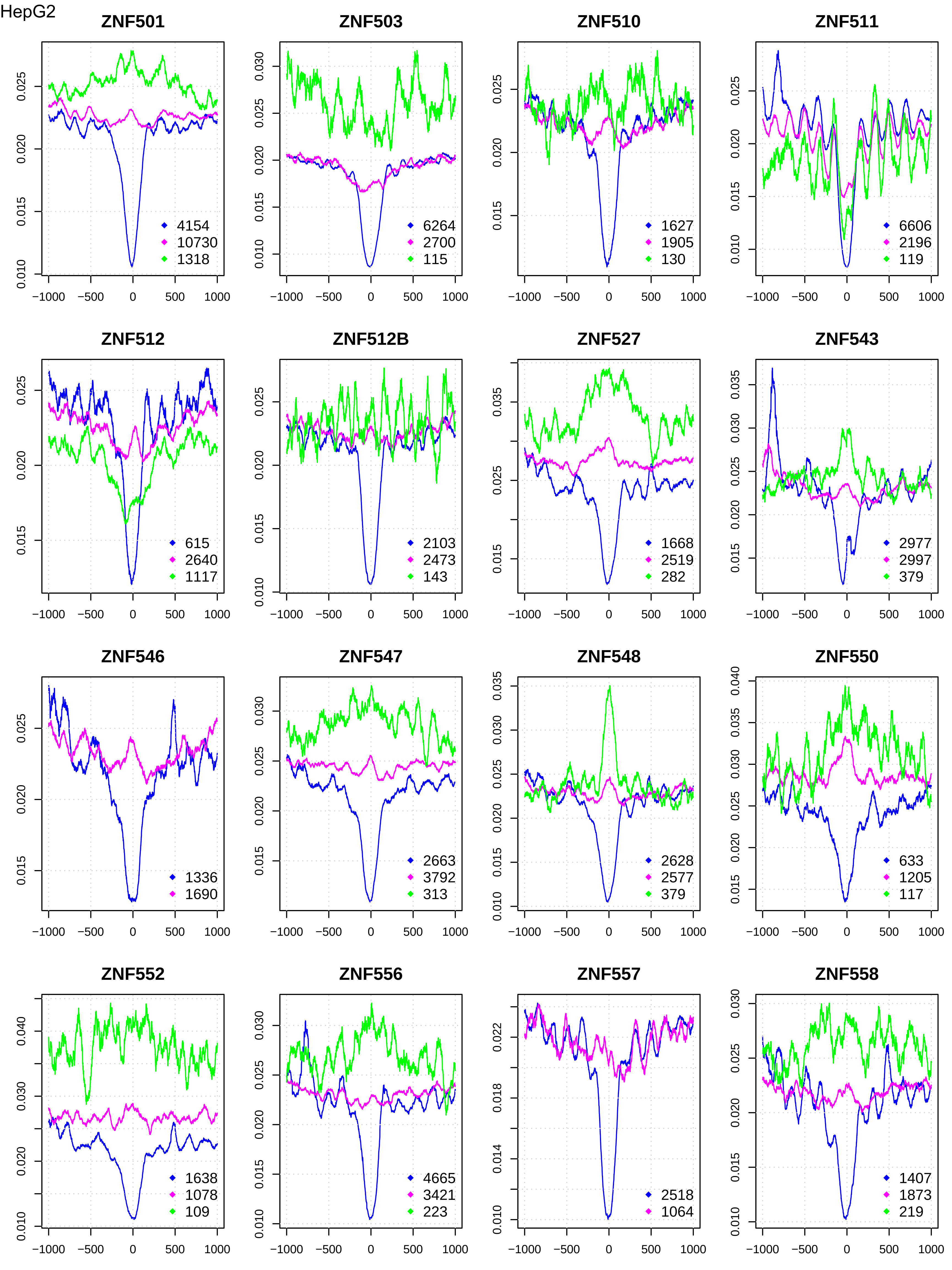
HepG2
ZNF501
4154
10730
1318
ZNF503
6264
2700
115
ZNF510
1627
1905
130
ZNF511
6606
2196
119
ZNF512
615
2640
1117
ZNF512B
2103
2473
143
ZNF527
1668
2519
282
ZNF543
2977
2997
379
ZNF546
1336
1690
ZNF547
2663
3792
313
ZNF548
2628
2577
379
ZNF550
633
1205
117
ZNF552
1638
1078
109
ZNF556
4665
3421
223
ZNF557
2518
1064
ZNF558
1407
1873
219

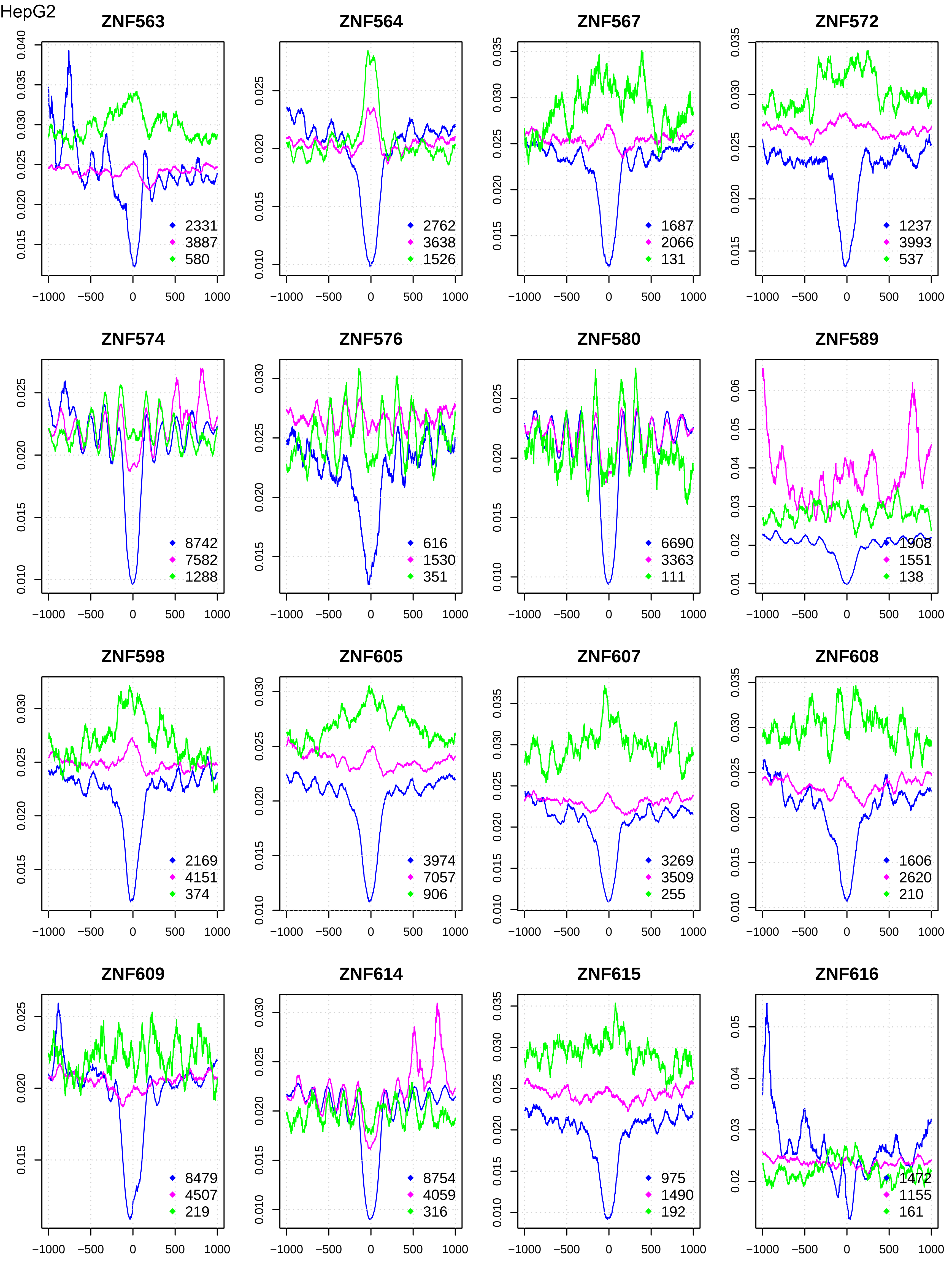
HepG2
ZNF563
2331
3887
580
ZNF564
2762
3638
1526
ZNF567
1687
2066
131
ZNF572
1237
3993
537
ZNF574
8742
7582
1288
ZNF576
616
1530
351
ZNF580
6690
3363
111
ZNF589
1908
1551
138
ZNF598
2169
4151
374
ZNF605
3974
7057
906
ZNF607
3269
3509
255
ZNF608
1606
2620
210
ZNF609
8479
4507
219
ZNF614
8754
4059
316
ZNF615
975
1490
192
ZNF616
1472
1155
161

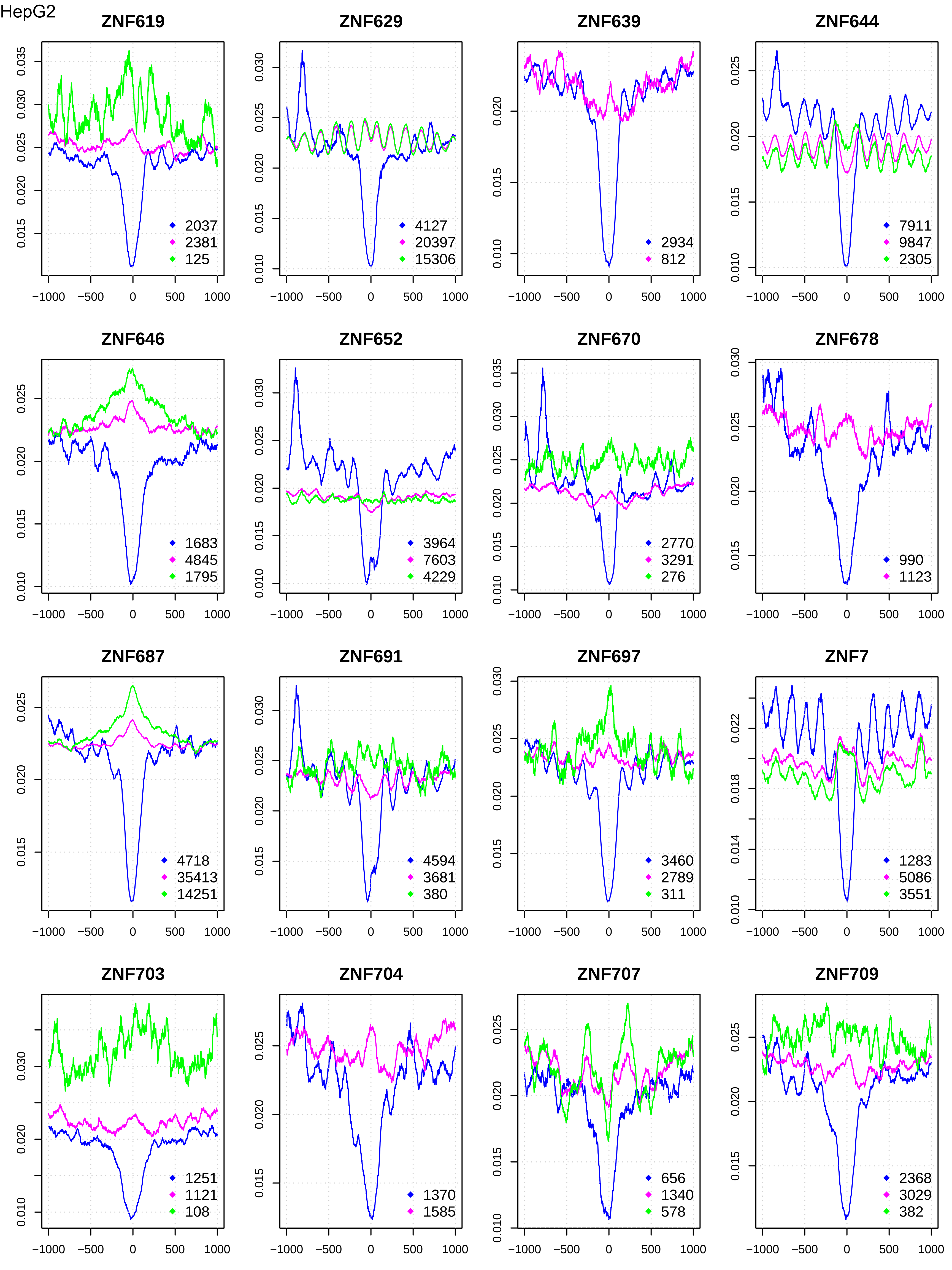
HepG2
ZNF619
2037
2381
125
ZNF629
4127
20397
15306
ZNF639
2934
812
ZNF644
7911
9847
2305
ZNF646
1683
4845
1795
ZNF652
3964
7603
4229
ZNF670
2770
3291
276
ZNF678
990
1123
ZNF687
4718
35413
14251
ZNF691
4594
3681
380
ZNF697
3460
2789
311
ZNF7
1283
5086
3551
ZNF703
1251
1121
108
ZNF704
1370
1585
ZNF707
656
1340
578
ZNF709
2368
3029
382
−1000
−500
0
500
1000

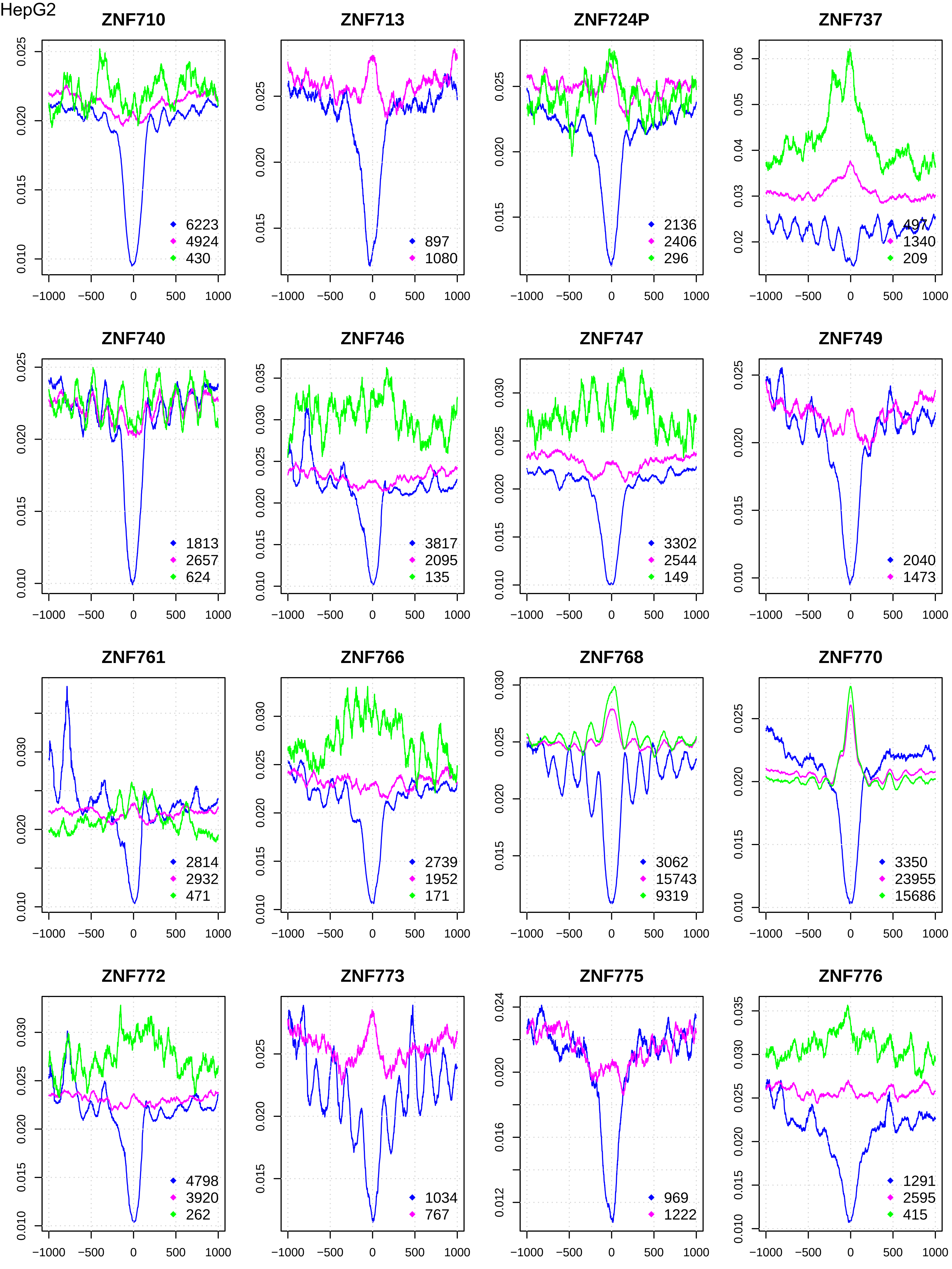
HepG2
ZNF710
6223
4924
430
ZNF713
897
1080
ZNF724P
2136
2406
296
ZNF737
497
1340
209
ZNF740
1813
2657
624
ZNF746
3817
2095
135
ZNF747
3302
2544
149
ZNF749
2040
1473
ZNF761
2814
2932
471
ZNF766
2739
1952
171
ZNF768
3062
15743
9319
ZNF770
3350
23955
15686
ZNF772
4798
3920
262
ZNF773
1034
767
ZNF775
969
1222
ZNF776
1291
2595
415

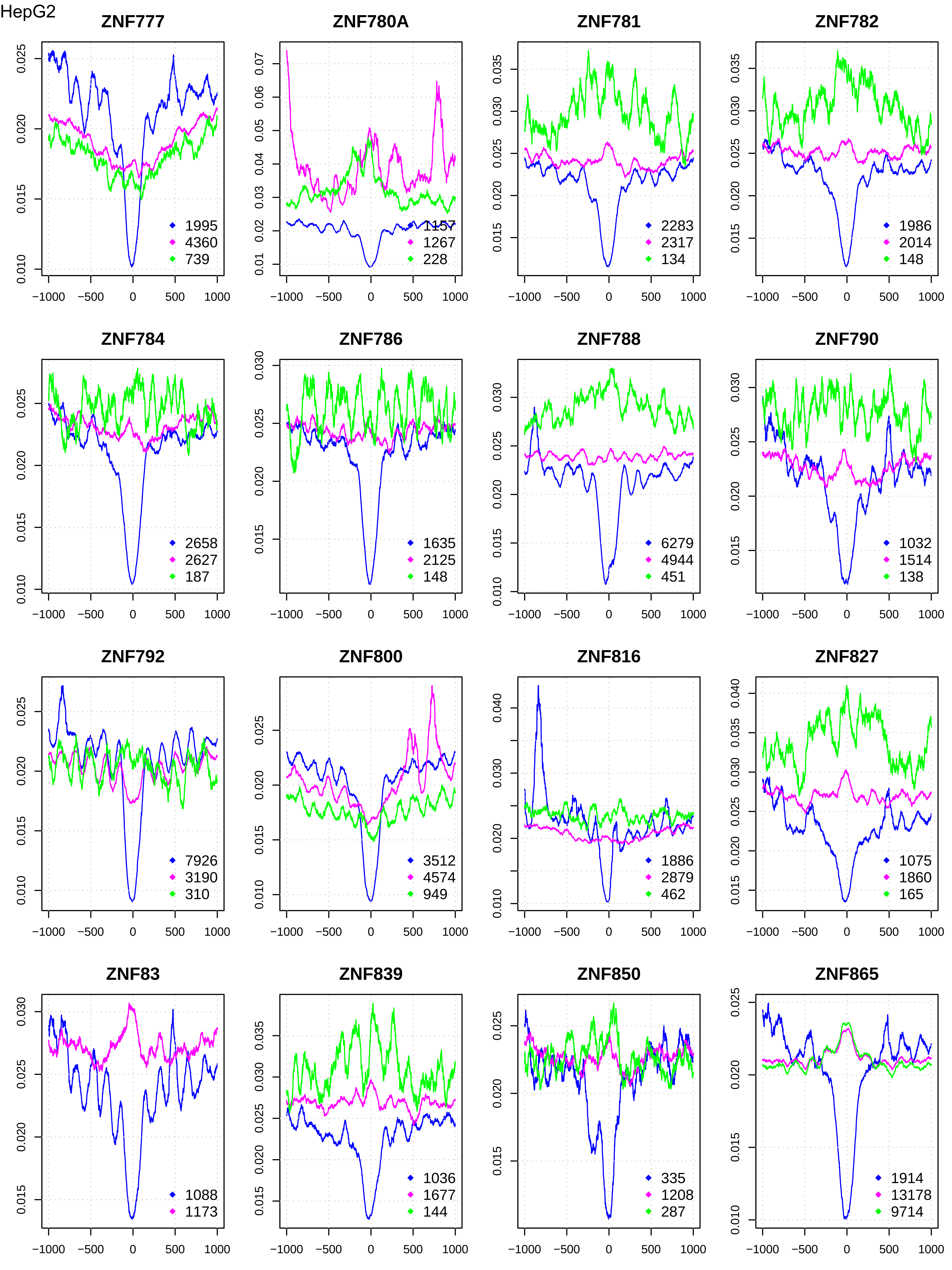
HepG2
ZNF777
1995
4360
739
ZNF780A
1157
1267
228
ZNF781
2283
2317
134
ZNF782
1986
2014
148
ZNF784
2658
2627
187
ZNF786
1635
2125
148
ZNF788
6279
4944
451
ZNF790
1032
1514
138
ZNF792
7926
3190
310
ZNF800
3512
4574
949
ZNF816
1886
2879
462
ZNF827
1075
1860
165
ZNF83
1088
1173
ZNF839
1036
1677
144
ZNF850
335
1208
287
ZNF865
1914
13178
9714

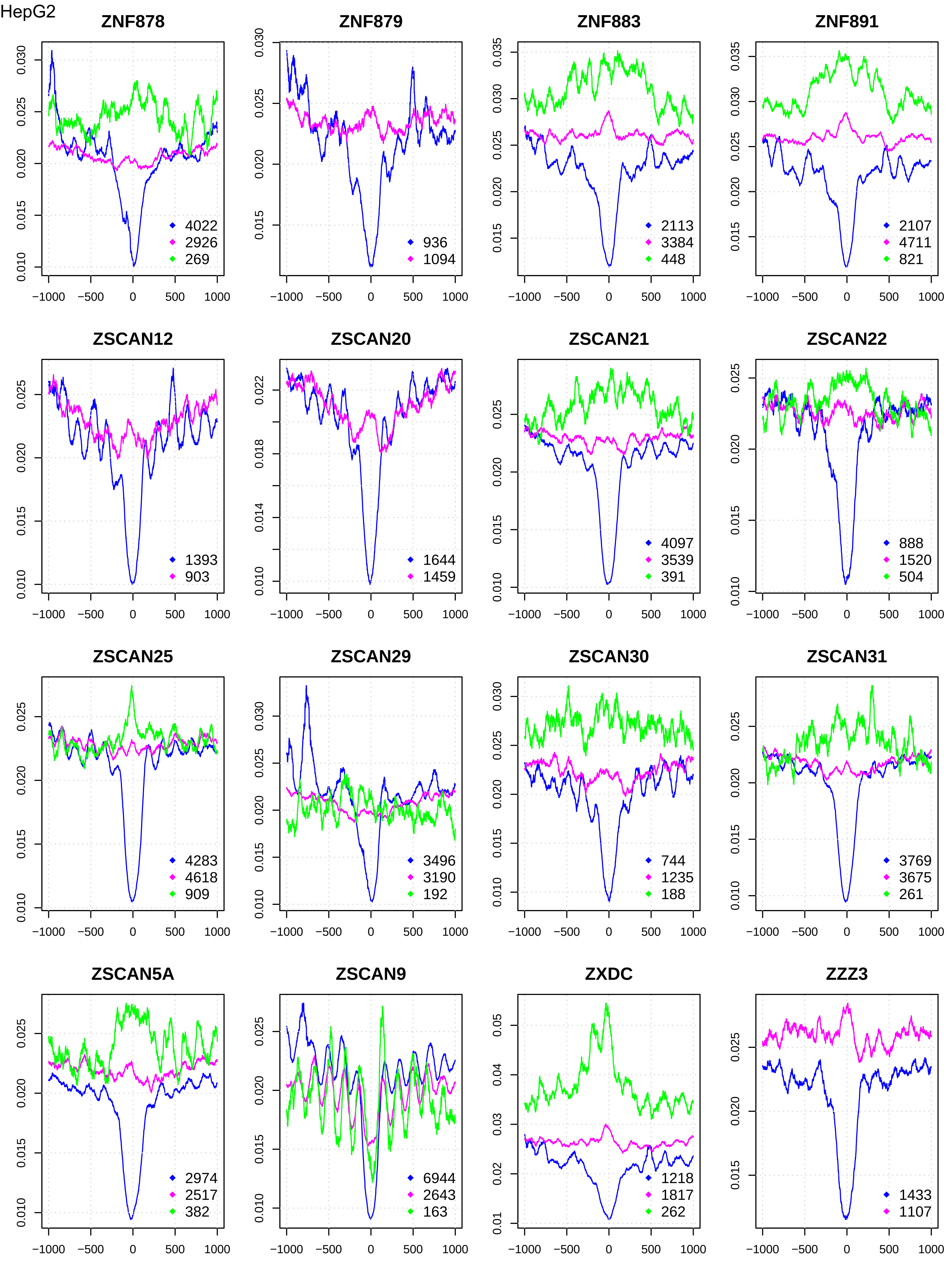
HepG2
ZNF878
4022
2926
269
ZNF879
936
1094
ZNF883
2113
3384
448
ZNF891
2107
4711
821
ZSCAN12
1393
903
ZSCAN20
1644
1459
ZSCAN21
4097
3539
391
ZSCAN22
888
1520
504
ZSCAN25
4283
4618
909
ZSCAN29
3496
3190
192
ZSCAN30
744
1235
188
ZSCAN31
3769
3675
261
ZSCAN5A
2974
2517
382
ZSCAN9
6944
2643
163
ZXDC
1218
1817
262
ZZZ3
1433
1107

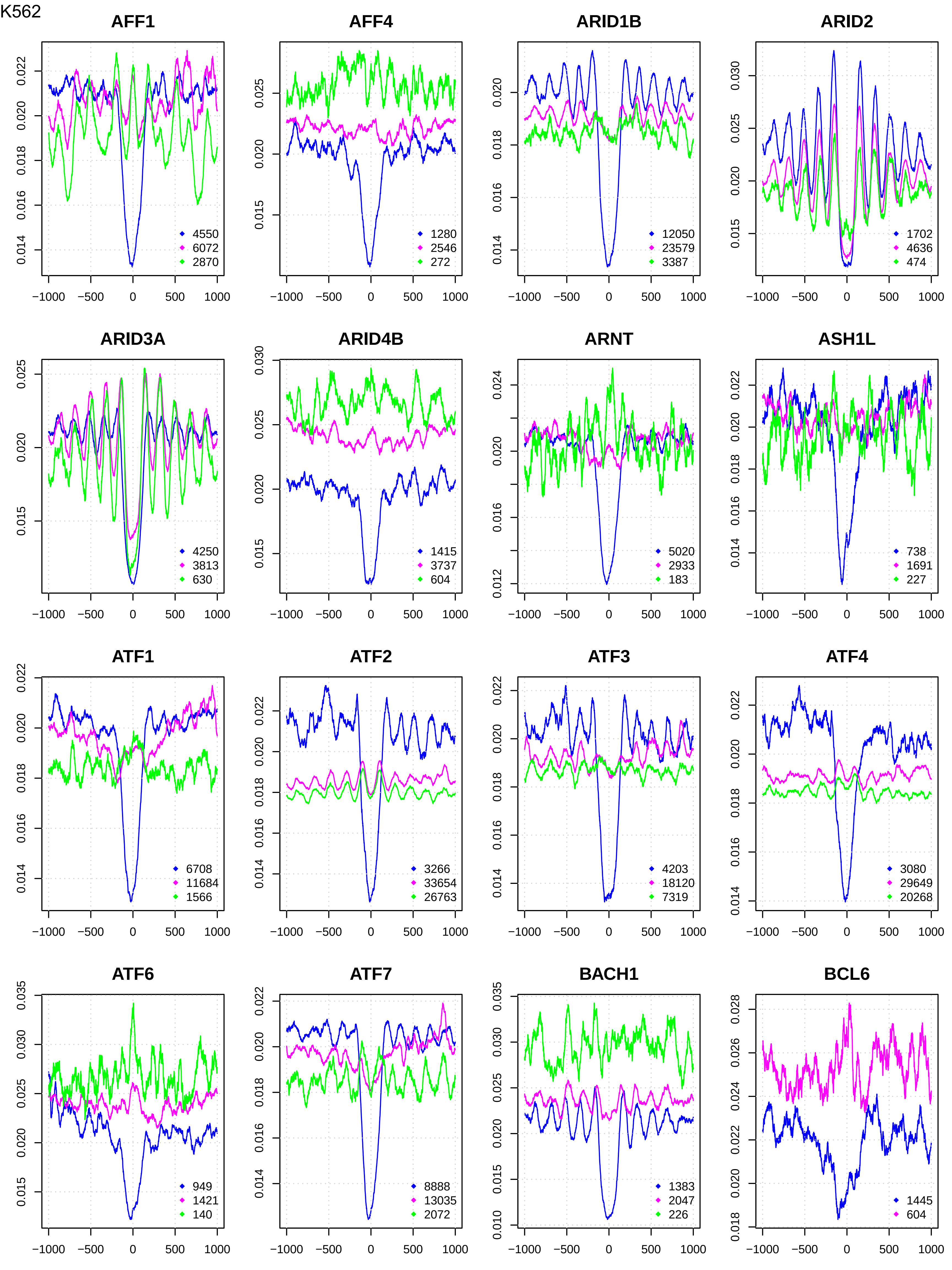

K562
AFF1
4550
6072
2870
AFF4
1280
2546
272
ARID1B
12050
23579
3387
ARID2
1702
4636
474
ARID3A
4250
3813
630
ARID4B
1415
3737
604
ARNT
5020
2933
183
ASH1L
738
1691
227
ATF1
6708
11684
1566
ATF2
3266
33654
26763
ATF3
4203
18120
7319
ATF4
3080
29649
20268
ATF6
949
1421
140
ATF7
8888
13035
2072
BACH1
1383
2047
226
BCL6
1445
604

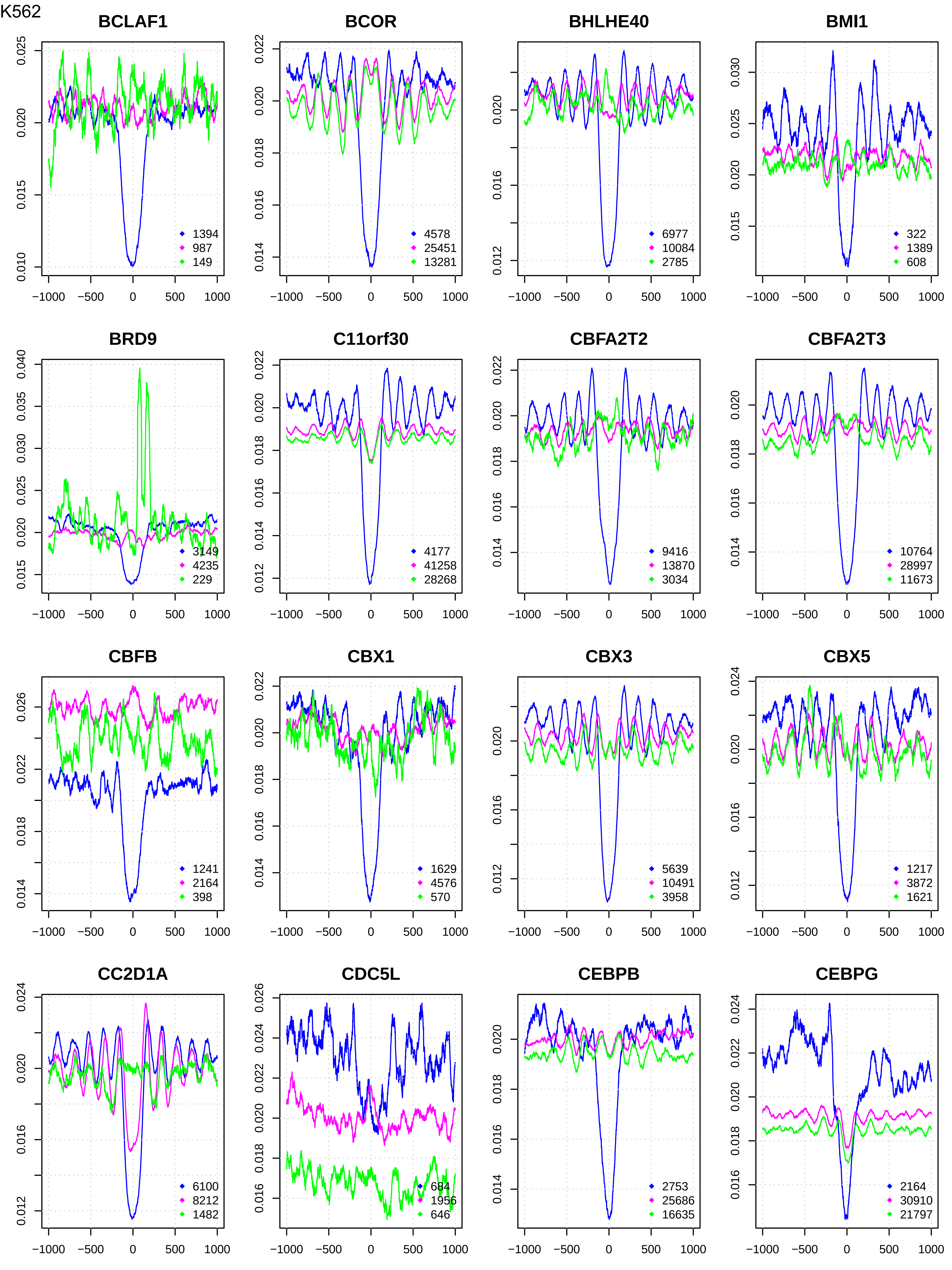
K562
BCLAF1
1394
987
149
BCOR
4578
25451
13281
BHLHE40
6977
10084
2785
BMI1
322
1389
608
BRD9
3149
4235
229
C11orf30
4177
41258
28268
CBFA2T2
9416
13870
3034
CBFA2T3
10764
28997
11673
CBFB
1241
2164
398
CBX1
1629
4576
570
CBX3
5639
10491
3958
CBX5
1217
3872
1621
CC2D1A
6100
8212
1482
CDC5L
684
1956
646
CEBPB
2753
25686
16635
CEBPG
2164
30910
21797

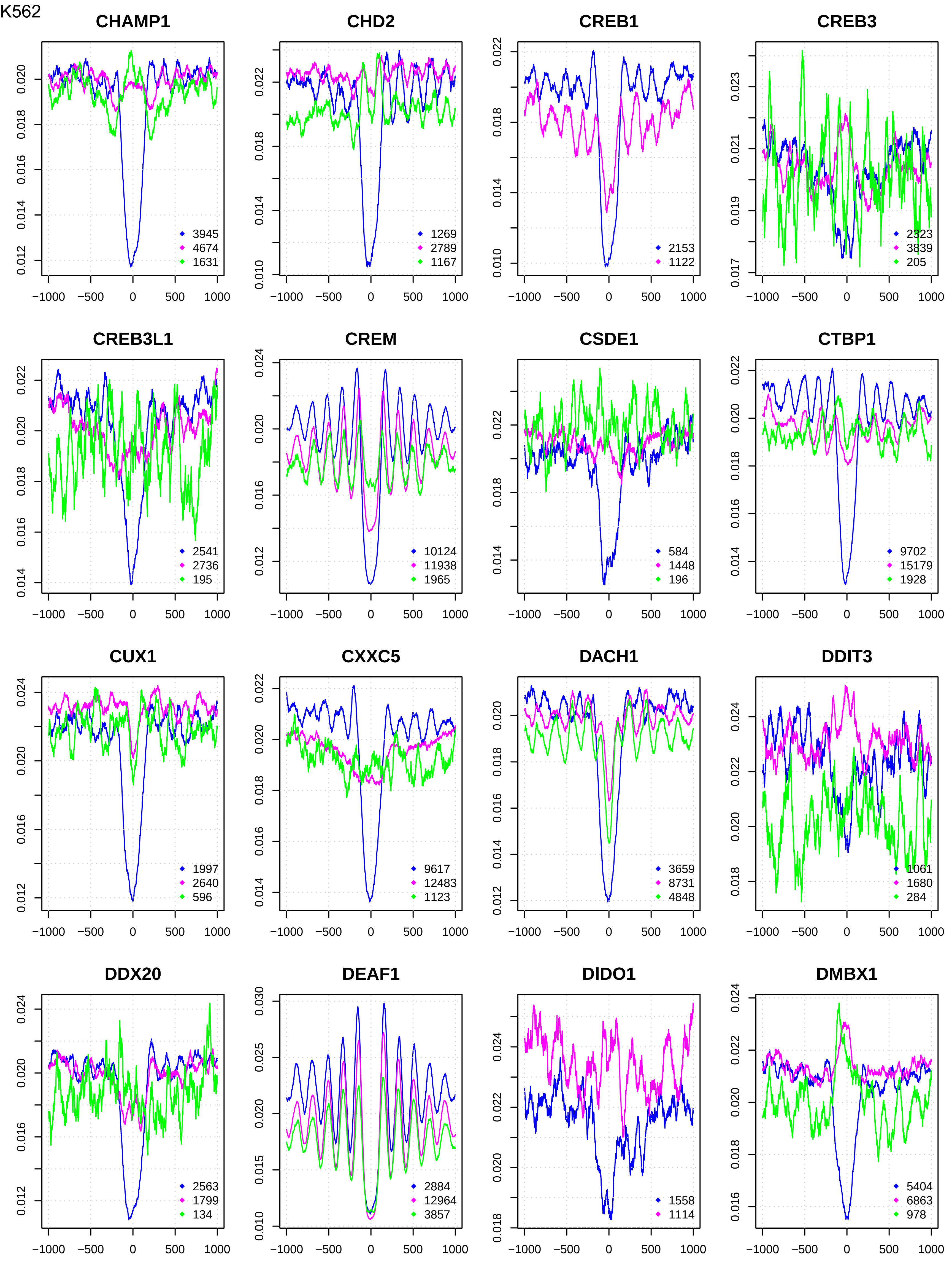
K562
CHAMP1
3945
4674
1631
CHD2
1269
2789
1167
CREB1
2153
1122
CREB3
2323
3839
205
CREB3L1
2541
2736
195
CREM
10124
11938
1965
CSDE1
584
1448
196
CTBP1
9702
15179
1928
CUX1
1997
2640
596
CXXC5
9617
12483
1123
DACH1
3659
8731
4848
DDIT3
1061
1680
284
DDX20
2563
1799
134
DEAF1
2884
12964
3857
DIDO1
1558
1114
DMBX1
5404
6863
978

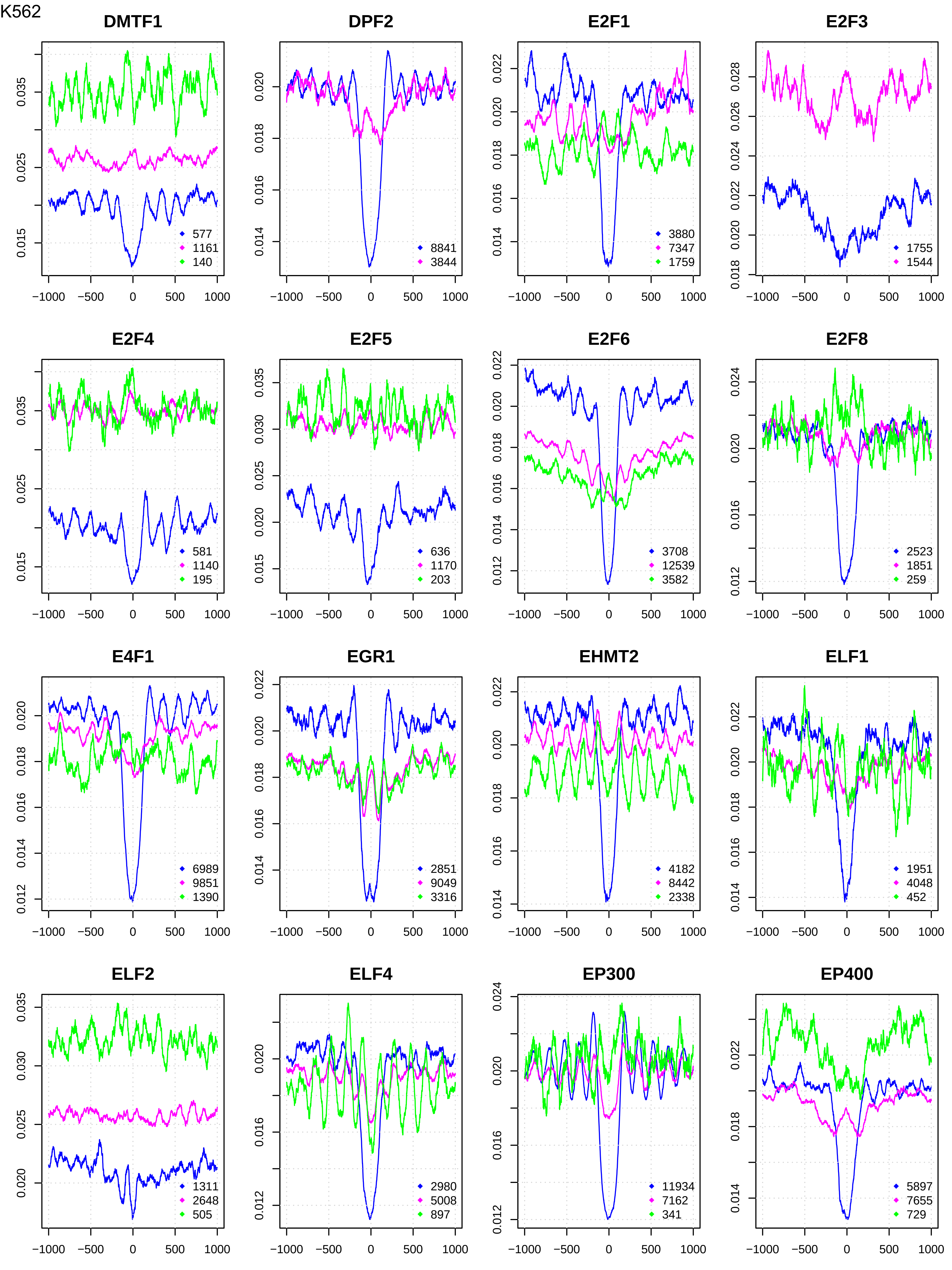
K562
DMTF1
577
1161
140
DPF2
8841
3844
E2F1
3880
7347
1759
E2F3
1755
1544
E2F4
581
1140
195
E2F5
636
1170
203
E2F6
3708
12539
3582
E2F8
2523
1851
259
E4F1
6989
9851
1390
EGR1
2851
9049
3316
EHMT2
4182
8442
2338
ELF1
1951
4048
452
ELF2
1311
2648
505
ELF4
2980
5008
897
EP300
11934
7162
341
EP400
5897
7655
729

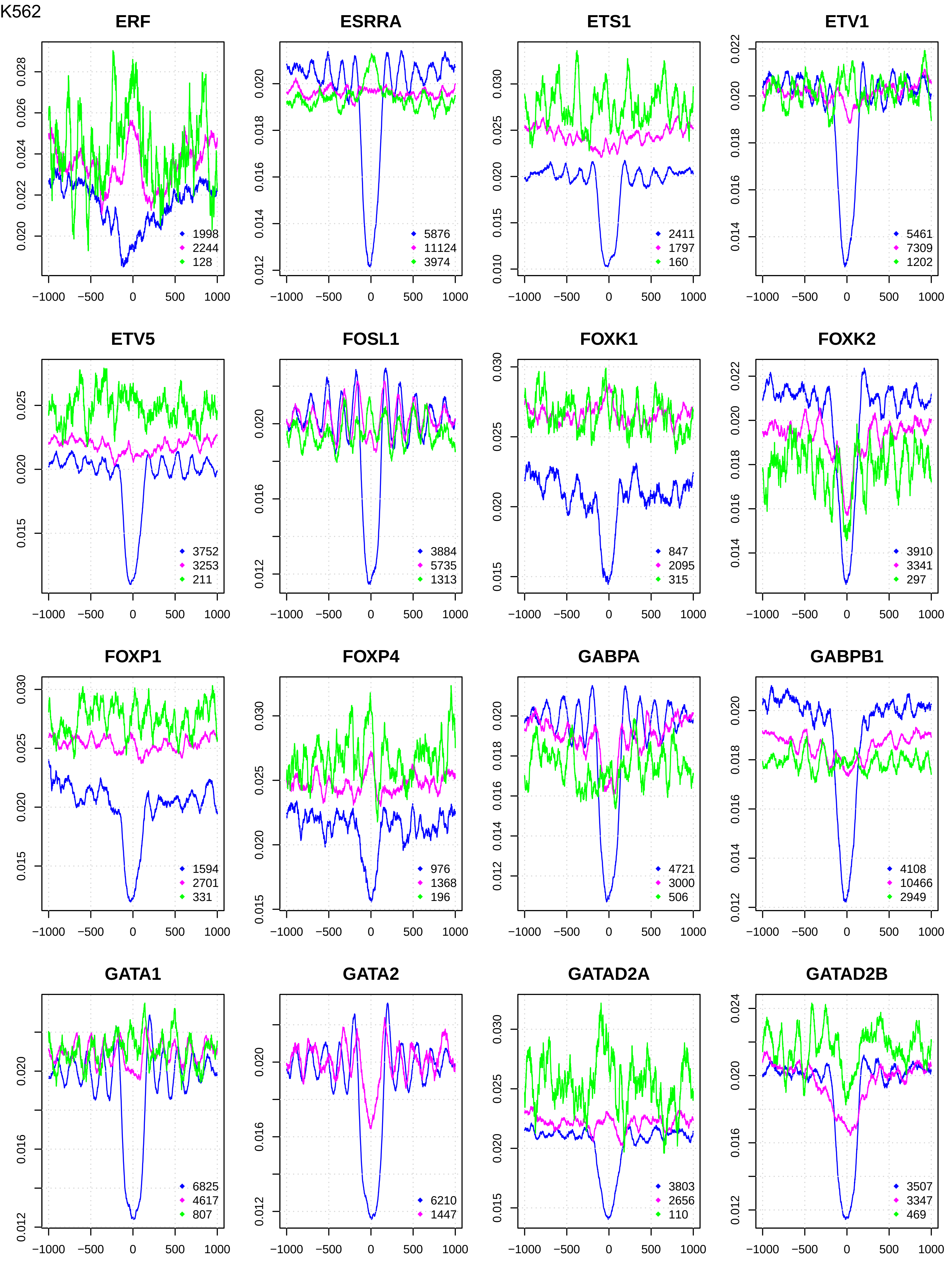
K562
ERF
1998
2244
128
ESRRA
5876
11124
3974
ETS1
2411
1797
160
ETV1
5461
7309
1202
ETV5
3752
3253
211
FOSL1
3884
5735
1313
FOXK1
847
2095
315
FOXK2
3910
3341
297
FOXP1
1594
2701
331
FOXP4
976
1368
196
GABPA
4721
3000
506
GABPB1
4108
10466
2949
GATA1
6825
4617
807
GATA2
6210
1447
GATAD2A
3803
2656
110
GATAD2B
3507
3347
469
−1000 −500 0 500 1000

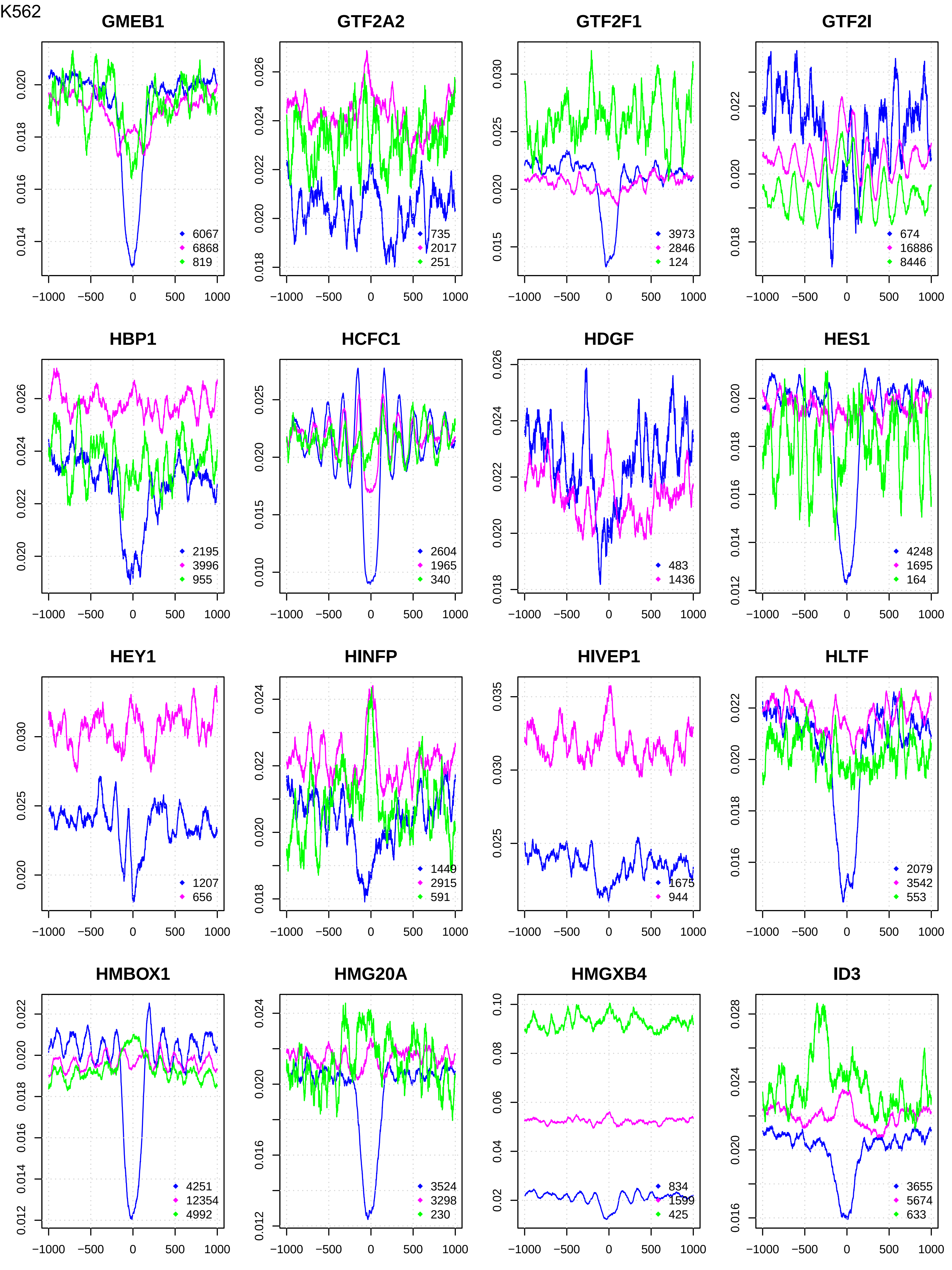
K562
GMEB1
6067
6868
819
GTF2A2
735
2017
251
GTF2F1
3973
2846
124
GTF2I
674
16886
8446
HBP1
2195
3996
955
HCFC1
2604
1965
340
HDGF
483
1436
HES1
4248
1695
164
HEY1
1207
656
HINFP
1449
2915
591
HIVEP1
1675
944
HLTF
2079
3542
553
HMBOX1
4251
12354
4992
HMG20A
3524
3298
230
HMGXB4
834
1599
425
ID3
3655
5674
633

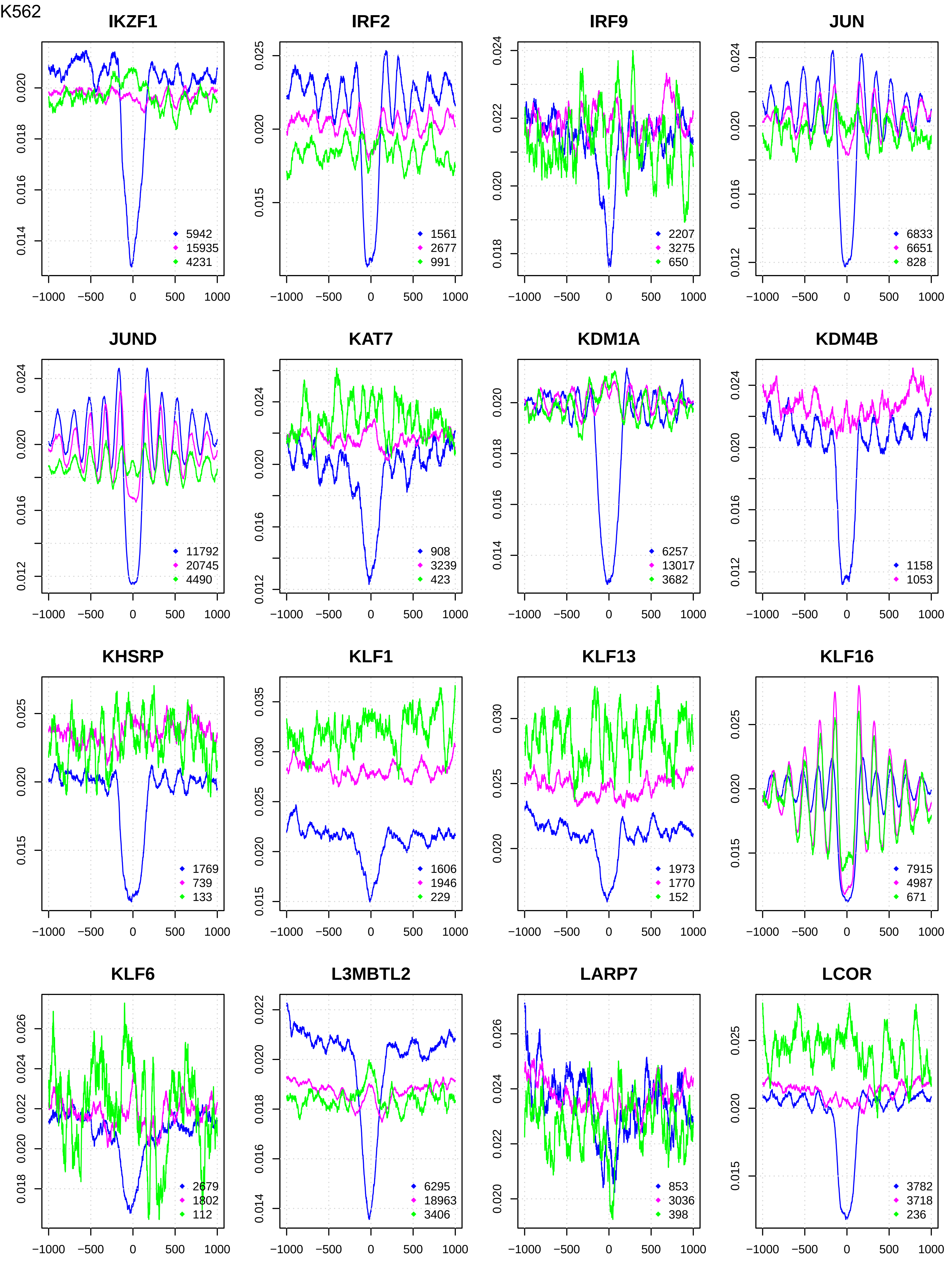
K562
IKZF1
5942
15935
4231
IRF2
1561
2677
991
IRF9
2207
3275
650
JUN
6833
6651
828
JUND
11792
20745
4490
KAT7
908
3239
423
KDM1A
6257
13017
3682
KDM4B
1158
1053
KHSRP
1769
739
133
KLF1
1606
1946
229
KLF13
1973
1770
152
KLF16
7915
4987
671
KLF6
2679
1802
112
L3MBTL2
6295
18963
3406
LARP7
853
3036
398
LCOR
3782
3718
236
−1000 −500 0 500 1000

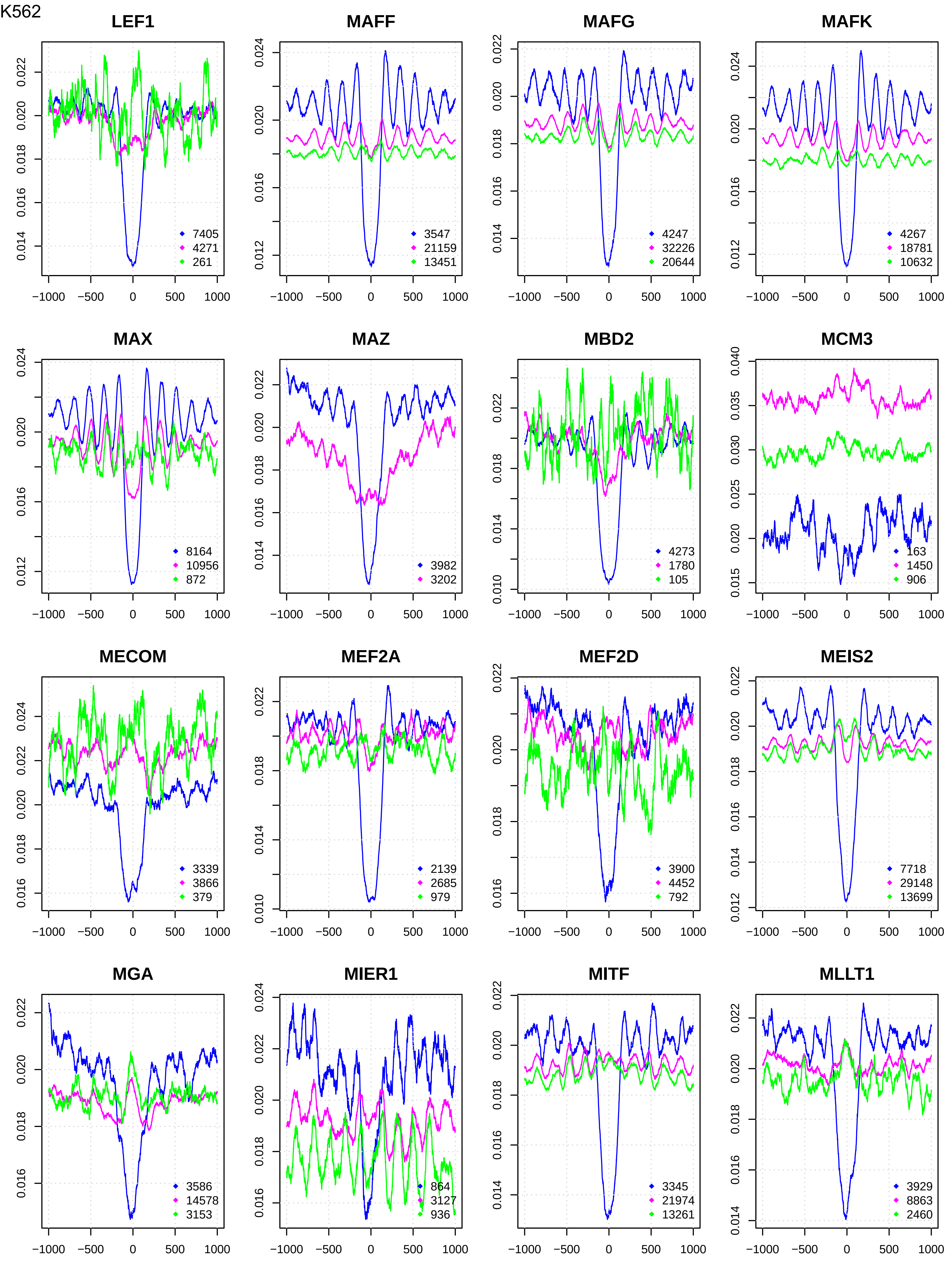
K562
LEF1
7405
4271
261
MAFF
3547
21159
13451
MAFG
4247
32226
20644
MAFK
4267
18781
10632
MAX
8164
10956
872
MAZ
3982
3202
MBD2
4273
1780
105
MCM3
163
1450
906
MECOM
3339
3866
379
MEF2A
2139
2685
979
MEF2D
3900
4452
792
MEIS2
7718
29148
13699
MGA
3586
14578
3153
MIER1
864
3127
936
MITF
3345
21974
13261
MLLT1
3929
8863
2460

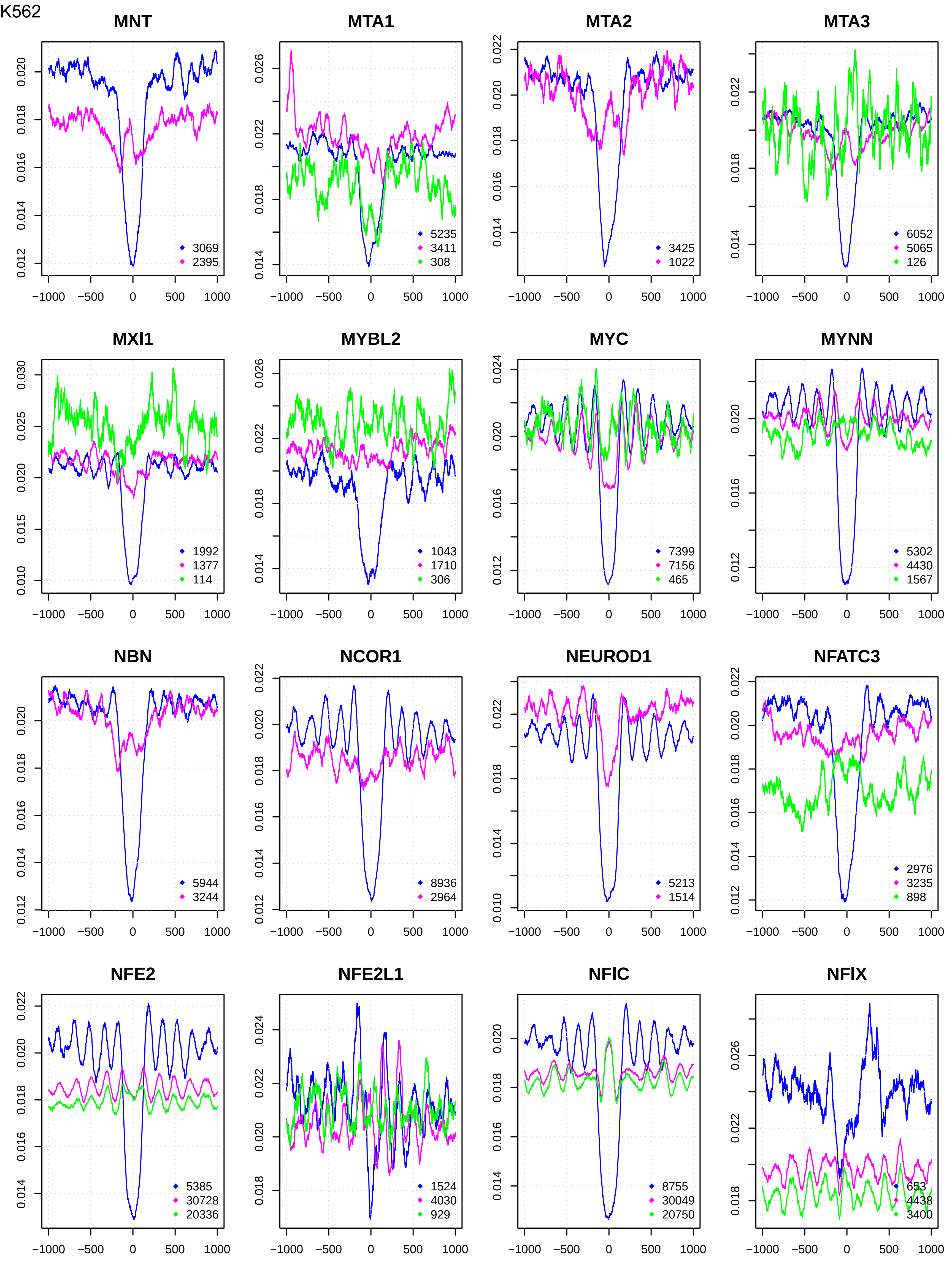
K562
MNT
0.012 0.014 0.016 0.018 0.020
3069
2395
MTA1
0.014 0.018 0.022 0.026
5235
3411
308
MTA2
0.014 0.016 0.018 0.020 0.022
3425
1022
MTA3
0.014 0.018 0.022
6052
5065
126
MXI1
0.010 0.015 0.020 0.025 0.030
1992
1377
114
MYBL2
0.014 0.018 0.022 0.026
1043
1710
306
MYC
0.012 0.016 0.020 0.024
7399
7156
465
MYNN
0.012 0.016 0.020
5302
4430
1567
NBN
0.012 0.014 0.016 0.018 0.020
5944
3244
NCOR1
0.012 0.014 0.016 0.018 0.020 0.022
8936
2964
NEUROD1
0.010 0.014 0.018 0.022
5213
1514
NFATC3
0.012 0.014 0.016 0.018 0.020 0.022
2976
3235
898
NFE2
0.014 0.016 0.018 0.020 0.022
5385
30728
20336
NFE2L1
0.018 0.020 0.022 0.024
1524
4030
929
NFIC
0.014 0.016 0.018 0.020
8755
30049
20750
NFIX
0.018 0.022 0.026
653
4438
3400
−1000 −500 0 500 1000

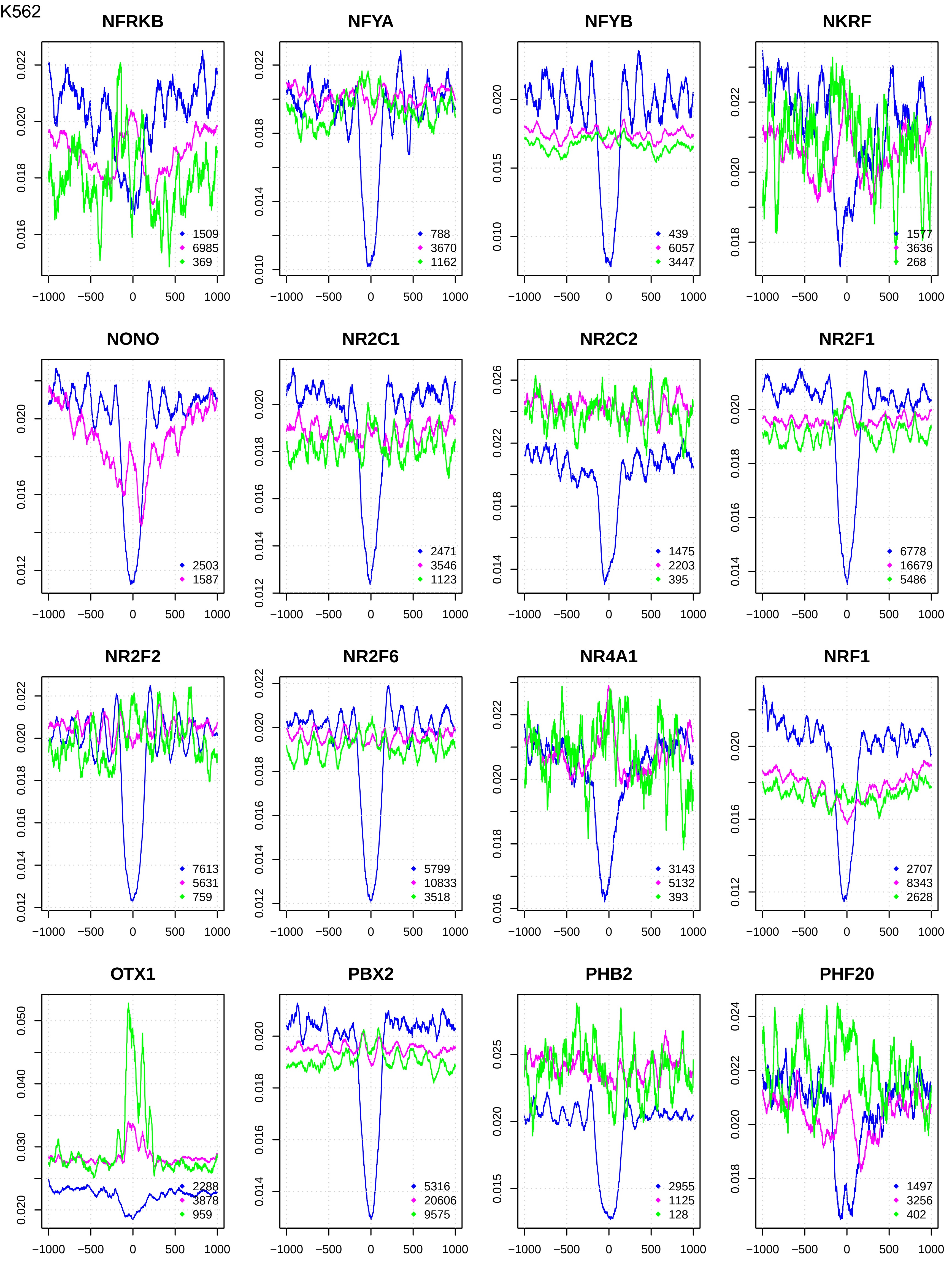
K562
NFRKB
1509
6985
369
NFYA
788
3670
1162
NFYB
439
6057
3447
NKRF
1577
3636
268
NONO
2503
1587
NR2C1
2471
3546
1123
NR2C2
1475
2203
395
NR2F1
6778
16679
5486
NR2F2
7613
5631
759
NR2F6
5799
10833
3518
NR4A1
3143
5132
393
NRF1
2707
8343
2628
OTX1
2288
3878
959
PBX2
5316
20606
9575
PHB2
2955
1125
128
PHF20
1497
3256
402
−1000 −500 0 500 1000

K562

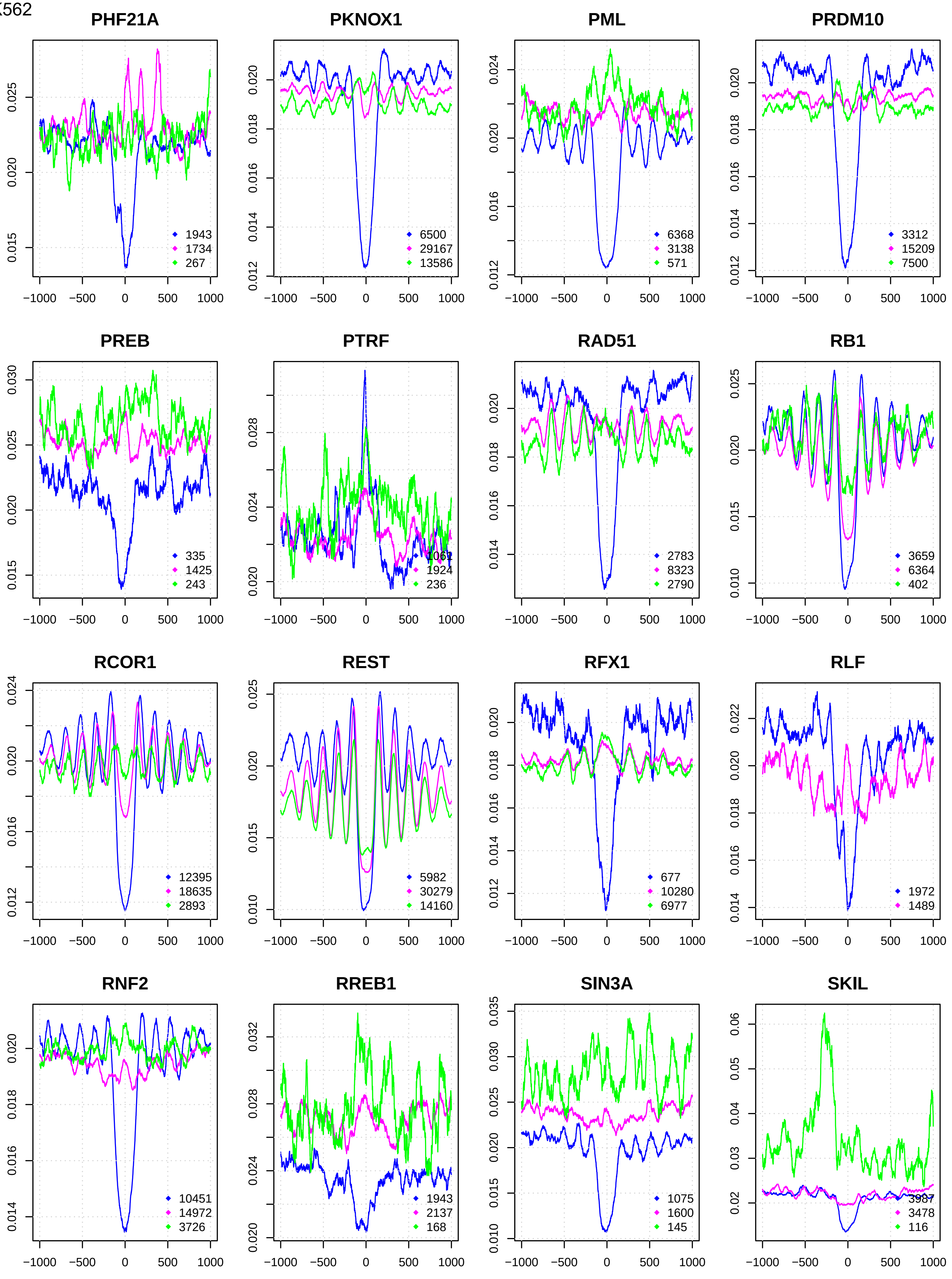
PHF21A
1943
1734
267
PKNOX1
6500
29167
13586
PML
6368
3138
571
PRDM10
3312
15209
7500
PREB
335
1425
243
PTRF
1061
1924
236
RAD51
2783
8323
2790
RB1
3659
6364
402
RCOR1
12395
18635
2893
REST
5982
30279
14160
RFX1
677
10280
6977
RLF
1972
1489
RNF2
10451
14972
3726
RREB1
1943
2137
168
SIN3A
1075
1600
145
SKIL
3987
3478
116

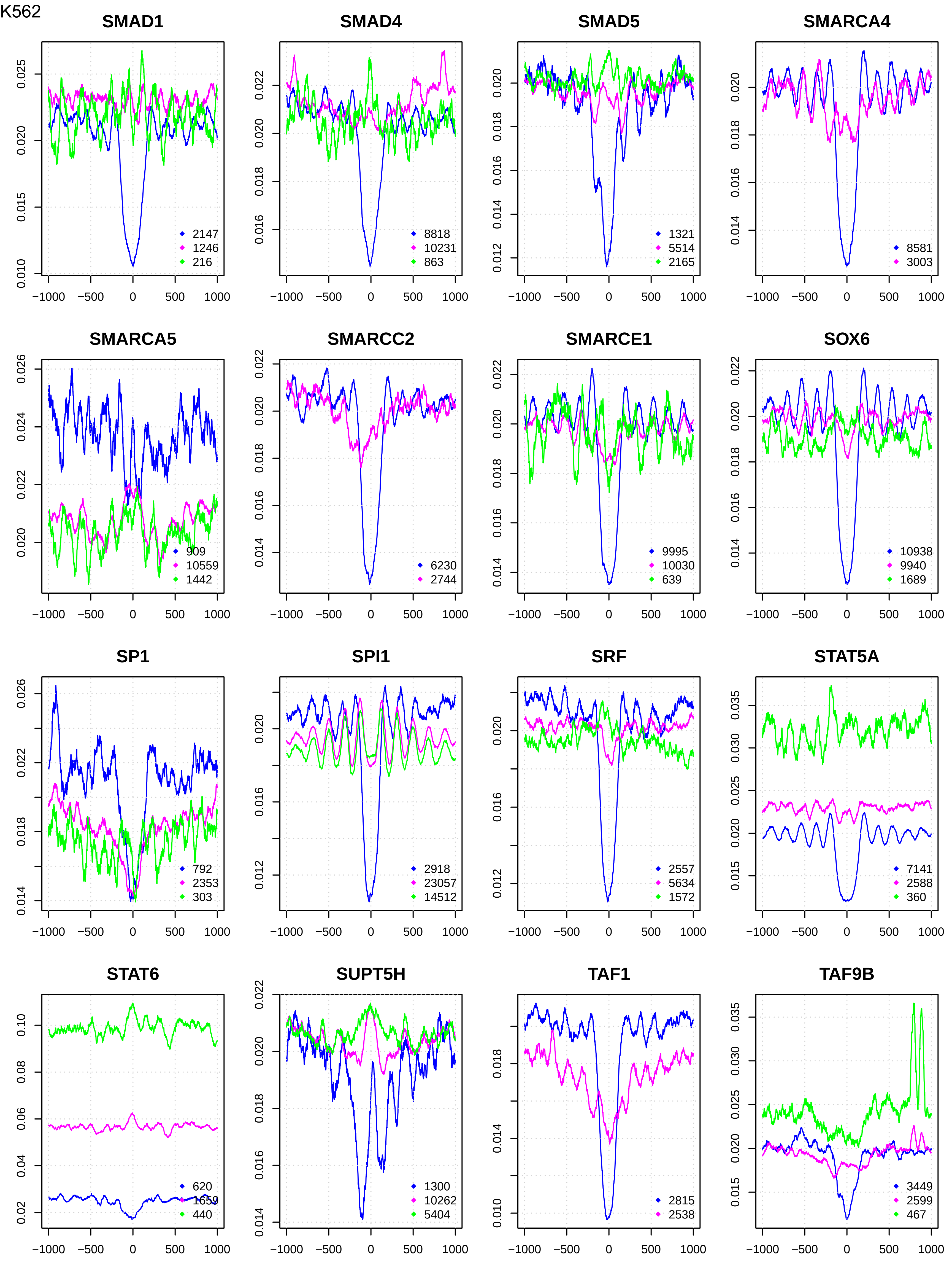
K562
SMAD1
2147
1246
216
SMAD4
8818
10231
863
SMAD5
1321
5514
2165
SMARCA4
8581
3003
SMARCA5
909
10559
1442
SMARCC2
6230
2744
SMARCE1
9995
10030
639
SOX6
10938
9940
1689
SP1
792
2353
303
SPI1
2918
23057
14512
SRF
2557
5634
1572
STAT5A
7141
2588
360
STAT6
620
1659
440
SUPT5H
1300
10262
5404
TAF1
2815
2538
TAF9B
3449
2599
467

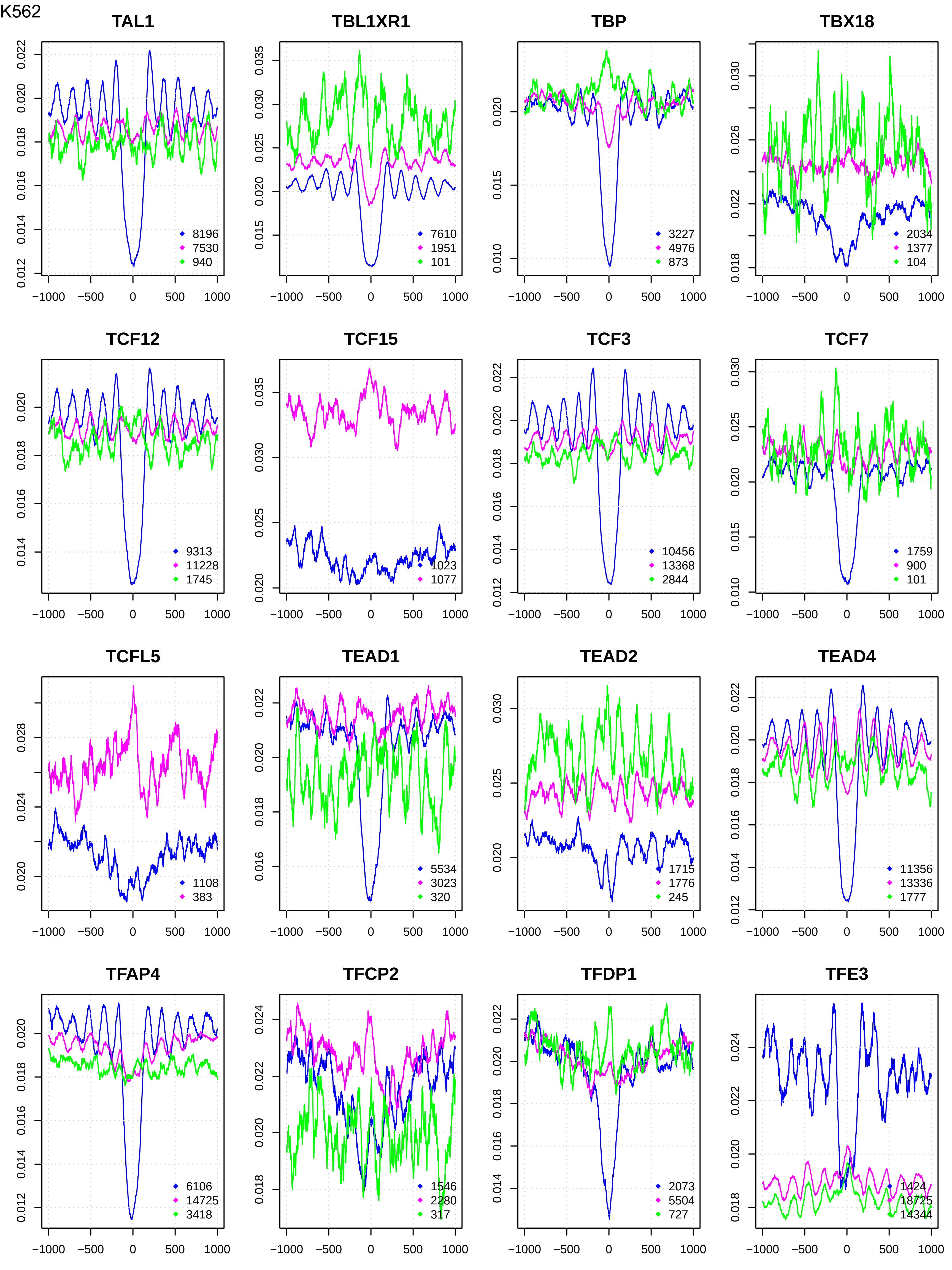
K562
TAL1
8196
7530
940
TBL1XR1
7610
1951
101
TBP
3227
4976
873
TBX18
2034
1377
104
TCF12
9313
11228
1745
TCF15
1023
1077
TCF3
10456
13368
2844
TCF7
1759
900
101
TCFL5
1108
383
TEAD1
5534
3023
320
TEAD2
1715
1776
245
TEAD4
11356
13336
1777
TFAP4
6106
14725
3418
TFCP2
1546
2280
317
TFDP1
2073
5504
727
TFE3
1424
18725
14344

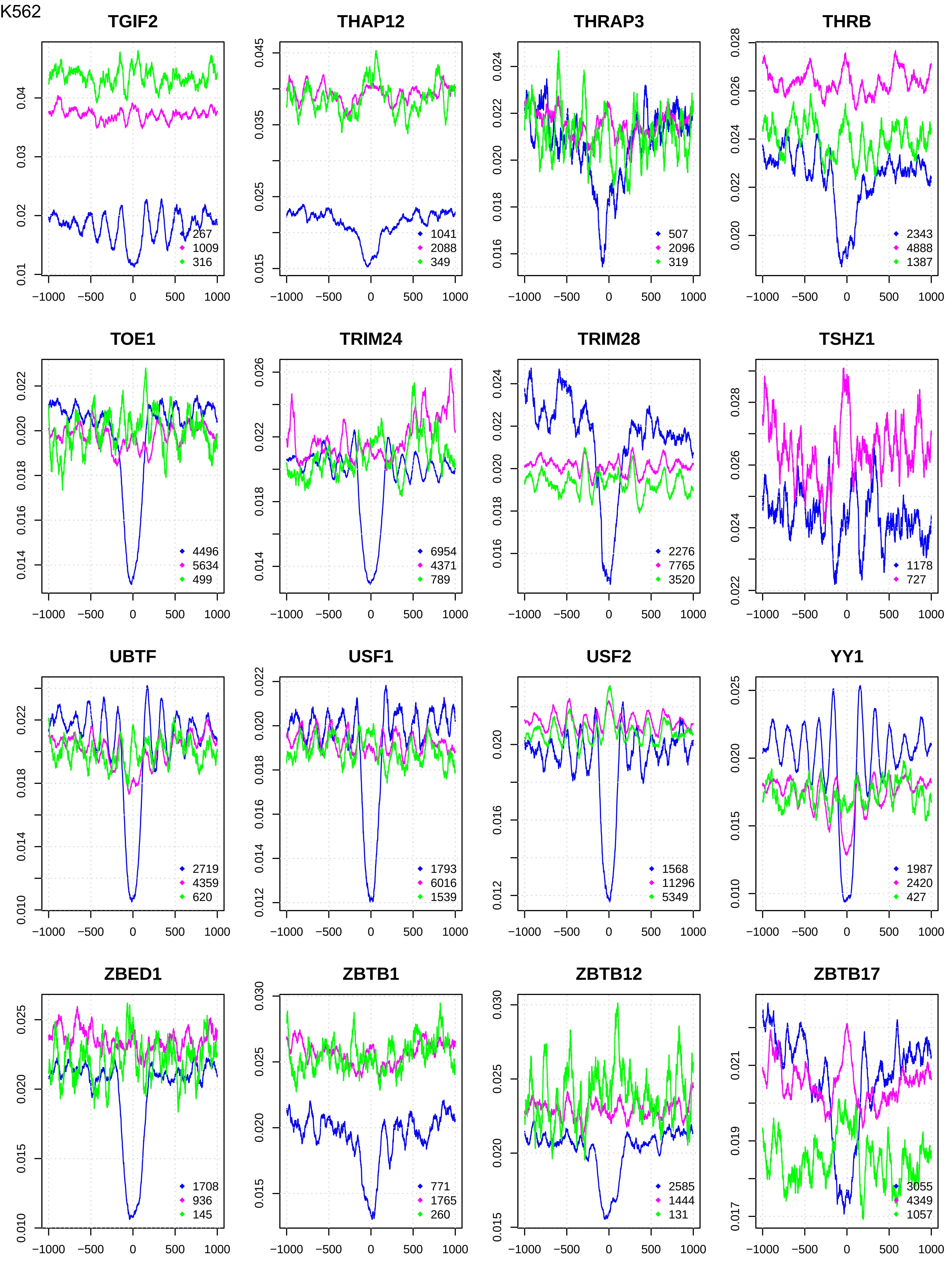
K562
TGIF2
267
1009
316
THAP12
1041
2088
349
THRAP3
507
2096
319
THRB
2343
4888
1387
TOE1
4496
5634
499
TRIM24
6954
4371
789
TRIM28
2276
7765
3520
TSHZ1
1178
727
UBTF
2719
4359
620
USF1
1793
6016
1539
USF2
1568
11296
5349
YY1
1987
2420
427
ZBED1
1708
936
145
ZBTB1
771
1765
260
ZBTB12
2585
1444
131
ZBTB17
3055
4349
1057

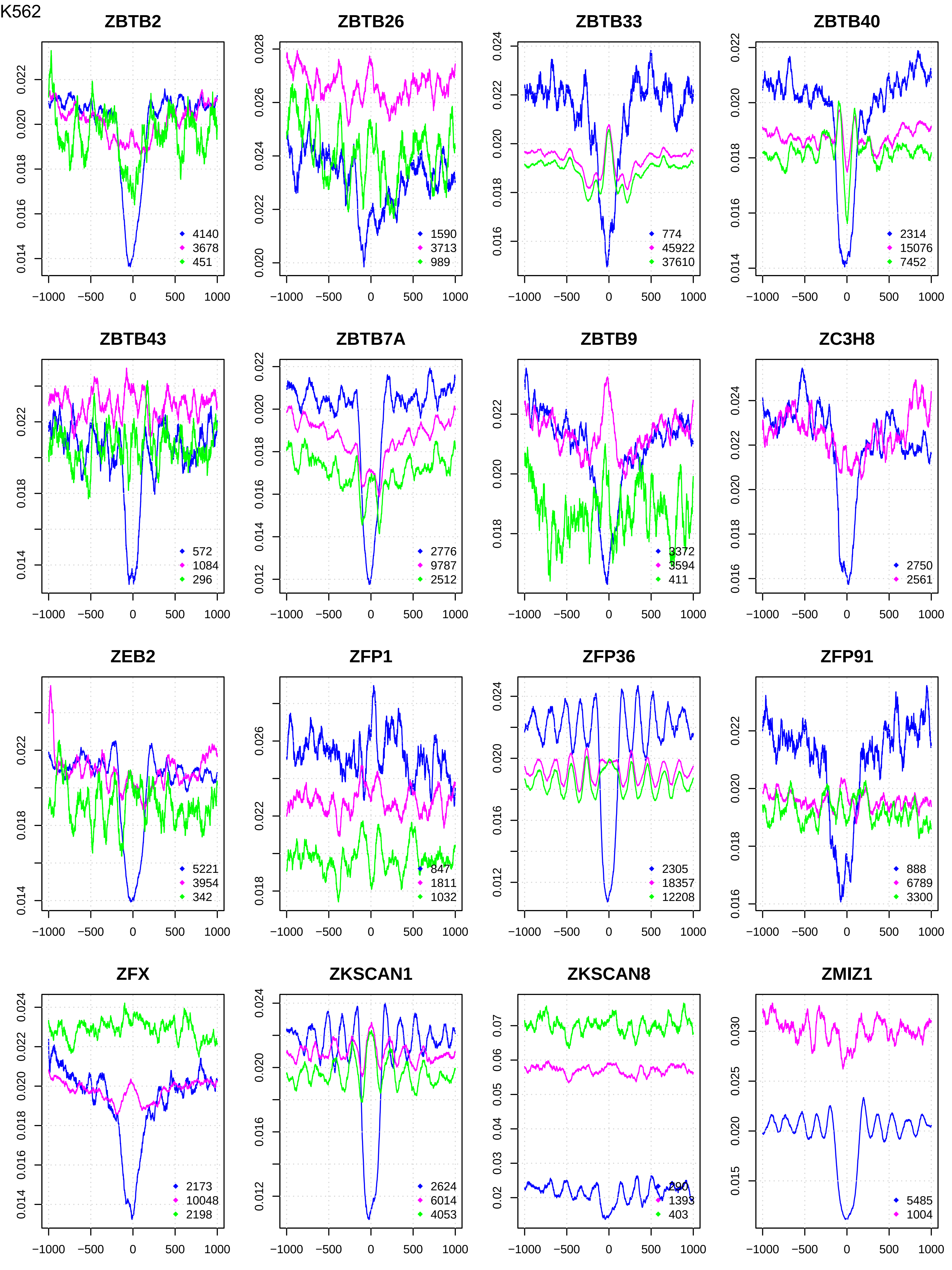
K562
ZBTB2
4140
3678
451
ZBTB26
1590
3713
989
ZBTB33
774
45922
37610
ZBTB40
2314
15076
7452
ZBTB43
572
1084
296
ZBTB7A
2776
9787
2512
ZBTB9
3372
3594
411
ZC3H8
2750
2561
ZEB2
5221
3954
342
ZFP1
847
1811
1032
ZFP36
2305
18357
12208
ZFP91
888
6789
3300
ZFX
2173
10048
2198
ZKSCAN1
2624
6014
4053
ZKSCAN8
290
1393
403
ZMIZ1
5485
1004

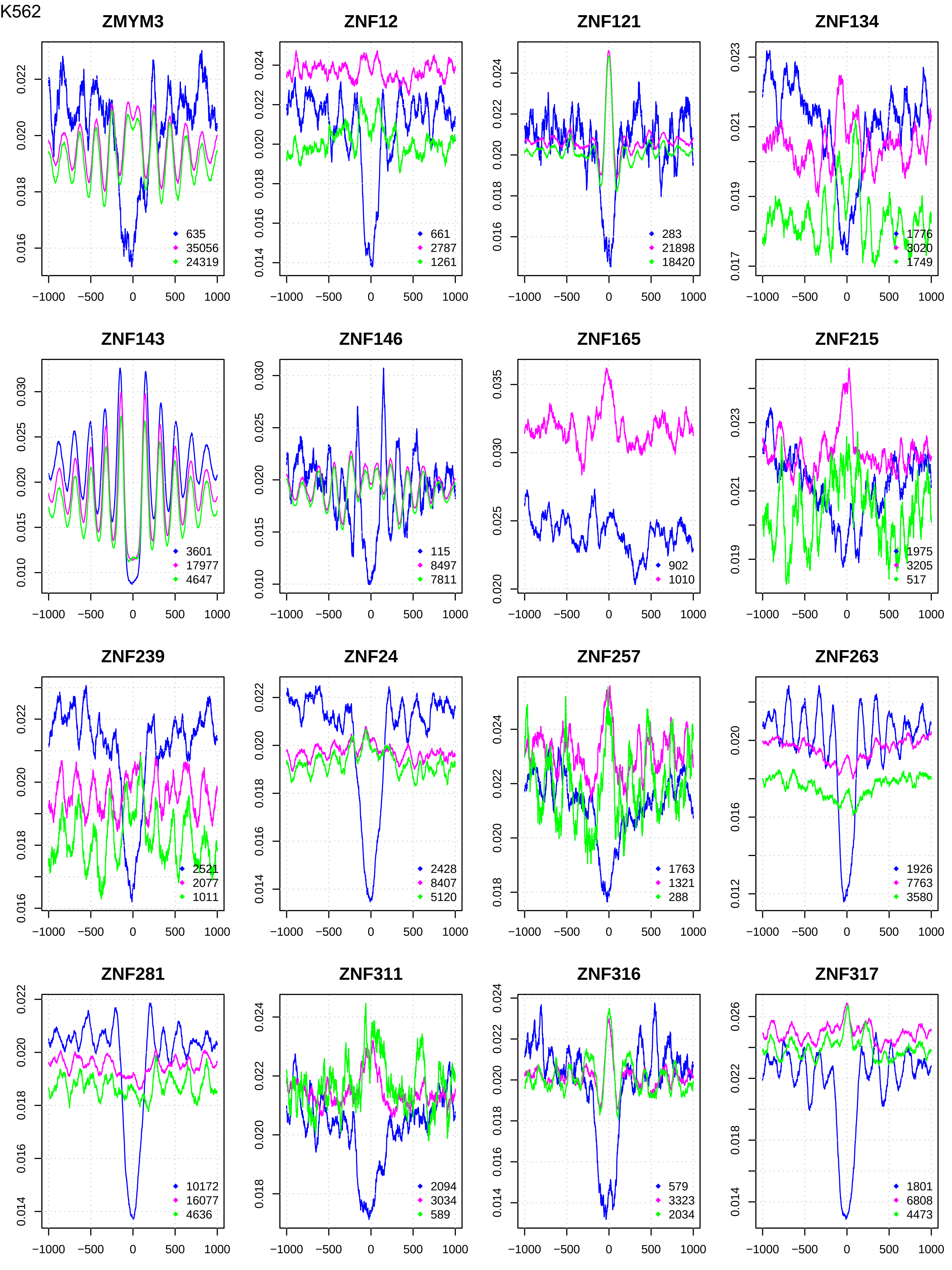
K562
ZMYM3
635
35056
24319
ZNF12
661
2787
1261
ZNF121
283
21898
18420
ZNF134
1776
3020
1749
ZNF143
3601
17977
4647
ZNF146
115
8497
7811
ZNF165
902
1010
ZNF215
1975
3205
517
ZNF239
2521
2077
1011
ZNF24
2428
8407
5120
ZNF257
1763
1321
288
ZNF263
1926
7763
3580
ZNF281
10172
16077
4636
ZNF311
2094
3034
589
ZNF316
579
3323
2034
ZNF317
1801
6808
4473

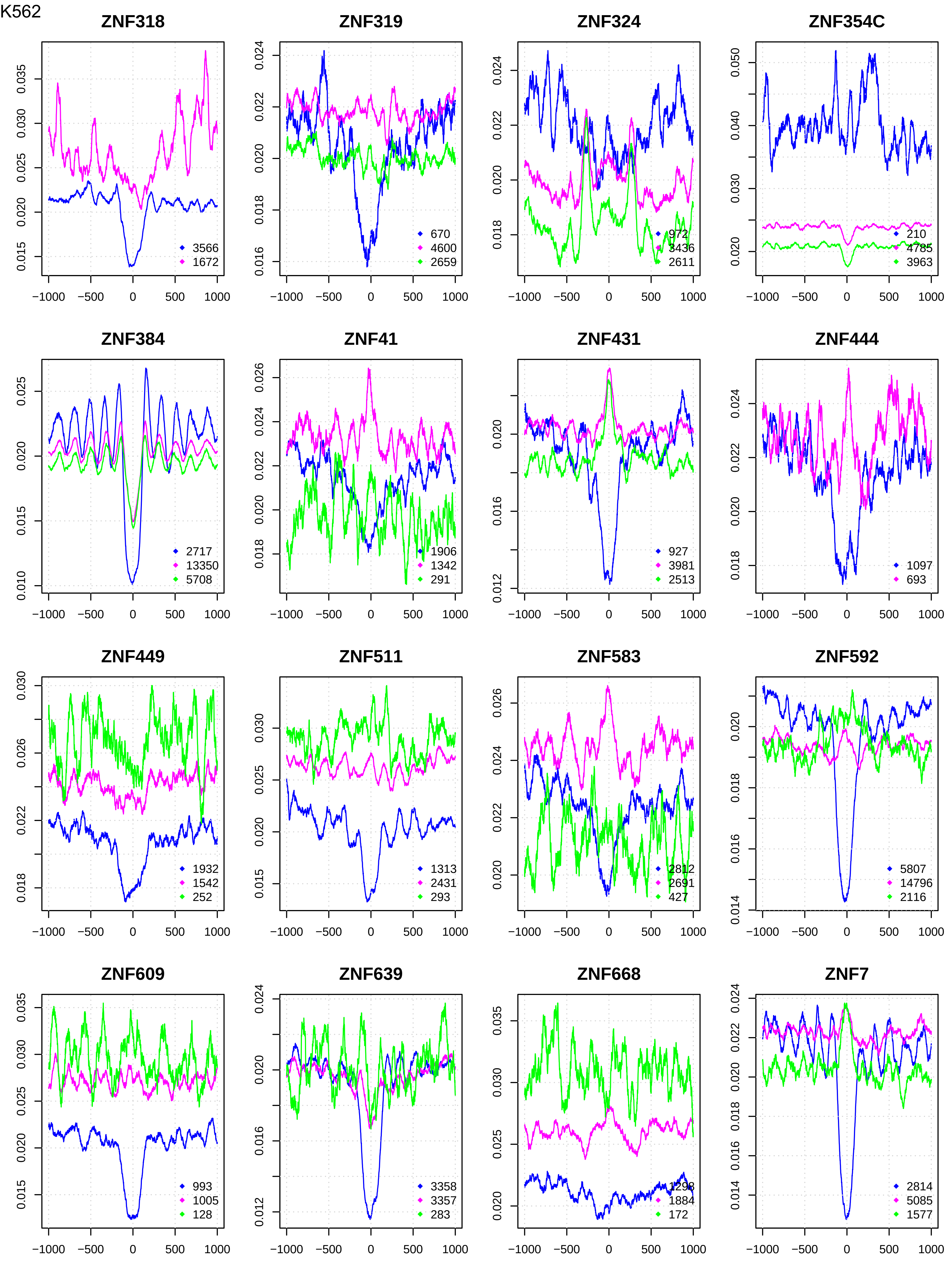
K562
ZNF318
3566
1672
ZNF319
670
4600
2659
ZNF324
972
3436
2611
ZNF354C
210
4785
3963
ZNF384
2717
13350
5708
ZNF41
1906
1342
291
ZNF431
927
3981
2513
ZNF444
1097
693
ZNF449
1932
1542
252
ZNF511
1313
2431
293
ZNF583
2812
2691
427
ZNF592
5807
14796
2116
ZNF609
993
1005
128
ZNF639
3358
3357
283
ZNF668
1298
1884
172
ZNF7
2814
5085
1577

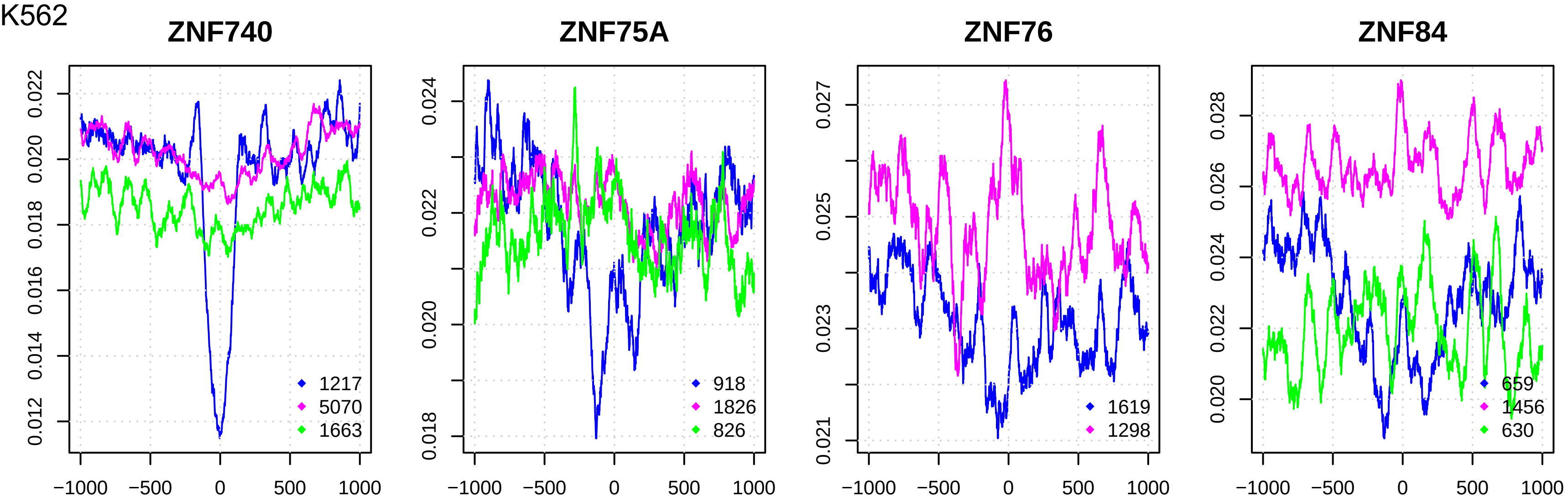

Figure S3 (page 11-61): Average Mnase-seq signal of the binding sites of a TF from three groups—its binding sites outside FBSs (magenta), the lonely sites that have less than 10 other TFs bound together (green) and its binding sites within the strong FBSs, i.e. FBSs_P95 (blue). The legends indicate the numbers of the binding sites of a TF in each group.

For some TFs the number of their lonely sites is less than 100. The plot (green line) is not shown, since we think the results from too few sites are not representative. Plots on page 11-43 are the results of TFs in HepG2 cells and plots on page 44-61 are the results of TFs in K562 cells.
